# Identification of virus-encoded microRNAs in divergent Papillomaviruses

**DOI:** 10.1371/journal.ppat.1007156

**Published:** 2018-07-26

**Authors:** Rachel Chirayil, Rodney P. Kincaid, Christine Dahlke, Chad V. Kuny, Nicole Dälken, Michael Spohn, Becki Lawson, Adam Grundhoff, Christopher S. Sullivan

**Affiliations:** 1 Center for Systems and Synthetic Biology, Center for Infectious Disease and Dept. Molecular Biosciences, The University of Texas at Austin, Austin, TX, United States of America; 2 Heinrich Pette Institute, Leibniz Institute for Experimental Virology, Hamburg, Germany; 3 Institute of Zoology, Zoological Society of London, London, United Kingdom; University of Wisconsin Madison School of Medicine and Public Health, UNITED STATES

## Abstract

MicroRNAs (miRNAs) are small RNAs that regulate diverse biological processes including multiple aspects of the host-pathogen interface. Consequently, miRNAs are commonly encoded by viruses that undergo long-term persistent infection. Papillomaviruses (PVs) are capable of undergoing persistent infection, but as yet, no widely-accepted PV-encoded miRNAs have been described. The incomplete understanding of PV-encoded miRNAs is due in part to lack of tractable laboratory models for most PV types. To overcome this, we have developed miRNA Discovery by forced Genome Expression (miDGE), a new wet bench approach to miRNA identification that screens numerous pathogen genomes in parallel. Using miDGE, we screened over 73 different PV genomes for the ability to code for miRNAs. Our results show that most PVs are unlikely to code for miRNAs and we conclusively demonstrate a lack of PV miRNA expression in cancers associated with infections of several high risk HPVs. However, we identified five different high-confidence or highly probable miRNAs encoded by four different PVs (Human PVs 17, 37, 41 and a *Fringilla coelebs* PV (FcPV1)). Extensive *in vitro* assays confirm the validity of these miRNAs in cell culture and two FcPV1 miRNAs are further confirmed to be expressed *in vivo* in a natural host. We show that miRNAs from two PVs (HPV41 & FcPV1) are able to regulate viral transcripts corresponding to the early region of the PV genome. Combined, these findings identify the first canonical PV miRNAs and support that miRNAs of either host or viral origin are important regulators of the PV life cycle.

## Author summary

Papillomaviruses (PVs) are causative agents of cancer. Currently, there is an incomplete understanding as to why only some infections lead to cancer. Developing a better comparative evolutionary understanding of PV gene products and their regulation is key to comprehending the life cycle of these pathogens. An emerging concept of viral gene regulation is that many persistent viruses will utilize small regulatory RNAs called miRNAs to optimize host and viral gene expression. Yet despite obvious interest, there have been no credible reports identifying canonical PV-encoded miRNAs. Here we develop new broadly applicable methodology to identify miRNAs from organisms lacking a laboratory culture system. We identify the first examples of bona fide canonical PV miRNAs and provide evidence supporting both host and viral miRNA-mediated regulation as relevant to control of PV gene expression. These findings resolve the issue of PV miRNAs and further the notion of miRNA importance to persistent virus infection.

## Introduction

Papillomaviruses (PVs) comprise a large family of circular double-stranded DNA viruses. Numerous PV genomes have been described including over 200 human PV (HPV) types. A minority of these are known as carcinogenic agents [1–3], however only a small fraction of hosts infected with these high risk types will go on to develop high grade lesions. It remains incompletely understood what factors dictate whether or not HPV infection will develop into malignant cancer [1,3]. Further, it is unclear why HPVs that share a high level of sequence similarity can have stark differences in tropism and infect different regions of the body. Developing a better understanding of PV gene products and their regulation throughout diverse PV lineages provides an evolutionary foundation for deciphering the mechanisms resulting in differential outcomes of infection.

MicroRNAs (miRNAs) are small regulatory RNAs that are an emerging class of viral gene products found in select virus families [4–7]. miRNAs are approximately 22 nucleotides long and function by docking to specific target mRNAs to repress translation [8,9]. miRNAs derive from primary transcripts containing a hairpin structure that is processed by a series of endonucleases (Drosha in the nucleus, Dicer in the cytosol) generating the final effector RNA [10–15]. The miRNA then enters a multi-protein complex called the RNA Induced Silencing Complex (RISC) where it scans mRNAs and docks to regions of partial sequence complementarity [8]. The so-called seed region of miRNAs, approximately 6 or more nucleotides towards the 5' end, typically binds transcripts with perfect complementarity and this feature can be used to help identify mRNA targets [8,16,17]. miRNAs of a particular sequence typically function by subtly regulating numerous (10 or more) mRNA transcripts involved in a particular biological outcome. Although individual miRNA regulation of any single transcript can be subtle, the sum of regulation by multiple miRNA-bound-RISC complexes (miRISC) can add up to significant biological activity [8]. miRNAs of host or viral origin have been implicated in various processes relevant to virus infection including the control of the immune response, cell death, transformation and virus gene expression [18–29].

Over 300 viral encoded miRNAs have been described, all from viruses able to undergo long term persistent infection [4,5,7,30]. Most of these viruses have DNA genomes including the herpes, polyoma, and anello virus families [5]. However, some retroviruses including the delta retrovirus bovine leukemia virus (BLV) and foamy retroviruses also encode miRNAs [21,31–33]. One likely role of viral miRNAs is to foster long-term interactions within the host [30]. In this regard, at least some members of the PV family would be expected to encode miRNAs. However, to date, no credible examples of PV canonical miRNAs have been described. Two studies examining fully infectious experimental systems of the high-risk HPV18 & 31 types report that these viruses do not encode miRNAs [34,35]. Further, at least two studies examining transformed cell lines report that HPV16 does not encode miRNAs [36,37]. There have been reports in transformed cells of small RNAs from high risk PVs such as HPVs 16 & 18, however these studies did not demonstrate a connection of PV-derived small RNAs to the miRNA biogenesis (Dicer/Drosha) or effector (RISC) machinery, nor did they confirm a biologically meaningful abundance [38–40]. Consequently, these RNAs are not widely accepted as miRNAs and the signal detected likely represents degradation fragments derived from the turnover of longer transcripts. In contrast, it is well documented that PV infection and individual PV gene products can alter the host miRNA repertoire, likely contributing to the biology of cancer [25,35,41–44]. Furthermore, at least one PV, HPV31, utilizes a host miRNA to directly regulate early viral gene expression [25]. Thus, what emerges is that although host miRNAs are involved in the PV life cycle and pathogenesis, of the few PV types that have been examined, no canonical PV miRNAs are yet established.

One barrier to discovery of PV miRNAs is the dearth of facile fully-infectious laboratory systems. There are experimental systems established for a few PV types (approximately < 5) [45], but technological barriers have limited any comprehensive large-scale study of viral miRNAs in the majority of PV types. Here we describe a new wet bench approach for the discovery of miRNAs that assays numerous viruses in parallel for the ability to express miRNAs. We identify bona fide PV miRNAs encoded by divergent PVs, and demonstrate these miRNAs depend on canonical miRNA biogenesis effector machinery. Our analysis also rules out canonical papillomaviral miRNAs in cancers associated with high-risk PV (HPVs 16, 18, 31, 45, and 58) infection. These results provide further evidence for the relevance of miRNA-mediated regulation of PV transcripts.

## Results

### Proof of concept for miDGE on a herpesviral genome

**Fig 1:**
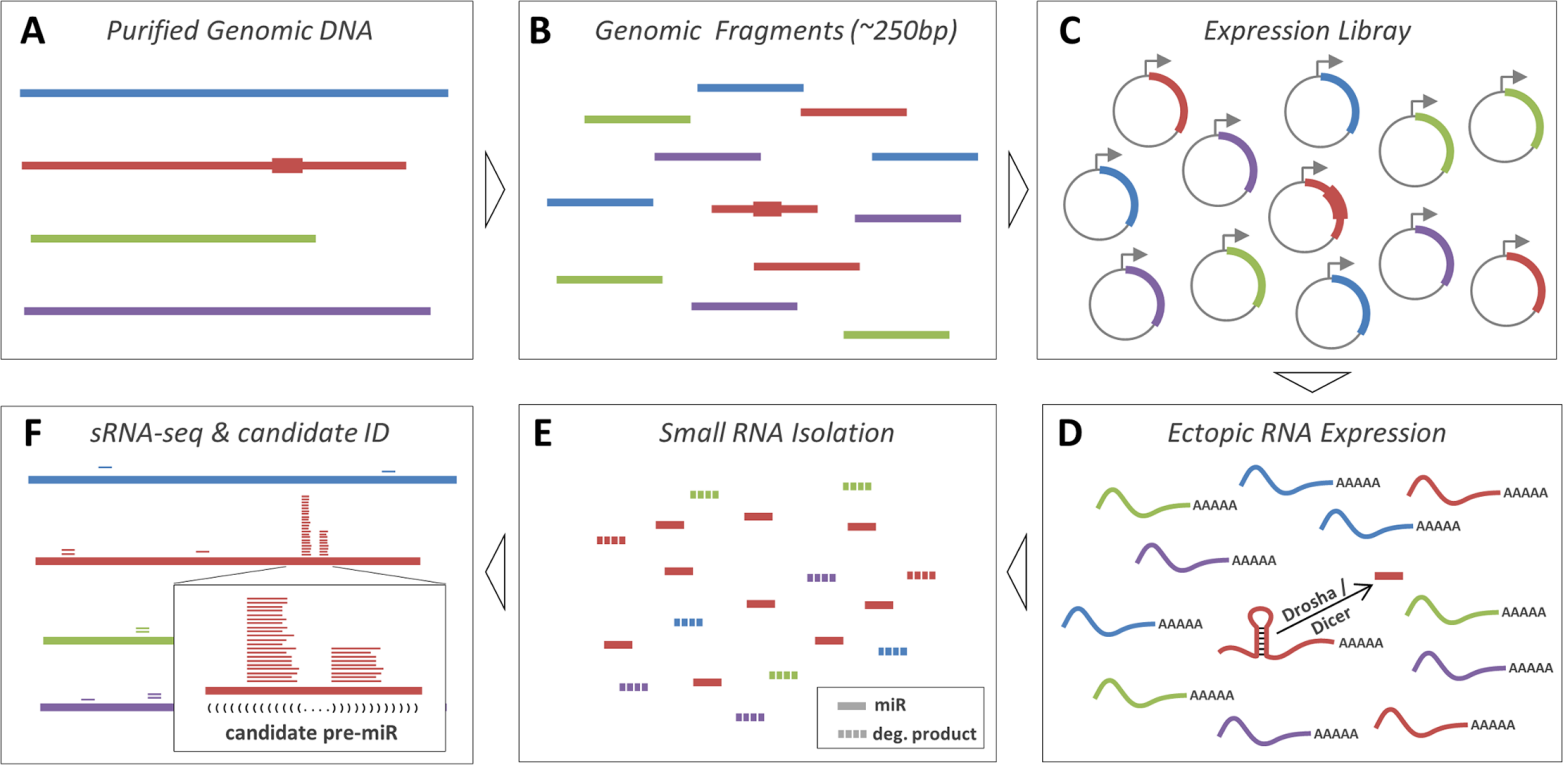
Overview of the miDGE (miRNA discovery by forced genomic expression) methodology. **(A)** Pools of purified viral genomes are subjected to **(B)** fragmentation via sonication or limited digest (average target size of fragments approx. 250 bp), followed by **(C)** cloning of subgenomic fragments to create an expression library. Thickened regions in A-C symbolize an unidentified pre-miRNA coding region present in one of the viral genomes. **(D)** After transient transfection, cloned subgenomic fragments are transcribed in cells, allowing processing of pre-miRNA hairpins to produce mature miRNAs. **(E)** Small RNAs purified from transfected cells will contain mature miRNAs as well as random degradation products produced from library transcripts. **(F)** To identify authentic miRNAs, small RNAs are sequenced and mapped back to viral genome pools. The majority of degradation products exhibit a random distribution, whereas miRNA products produce distinct pileups at pre-miRNA loci. Secondary structure prediction can then be used to identify characteristic pre-miRNA hairpin structures (indicated by bracket notation in F) at such loci. In this study, we used the miRDeep2 package to identify miRNA candidates.

To identify miRNA genes in situations where transcripts are not easily obtainable, we developed the approach of miRNA Discovery by forced Genomic Expression (miDGE). miDGE relies on generating a library of numerous overlapping genomic segments of DNA from a particular organism or locus and subcloning them behind a heterologous RNA polymerase (RNP) II promoter (Fig 1). The concept relies on the principle that miRNA genes are compact and should be readily expressed by heterologous upstream RNP II promoters, or in the rare cases that a primary miRNA transcript is driven by RNP III, that these promoters are small and proximal to the miRNA gene so as to be included in miDGE library constructs. The miDGE library is then transfected into mammalian cells and small RNA is harvested and sequenced. Next, we apply computational methods to identify miRNA candidates whose transcripts display the hallmarks of processing by the miRNA biogenesis machinery. Finally, candidate miRNAs are validated via a series of molecular assays to establish biogenesis via the canonical miRNA processing machinery and activity within RISC, the miRNA silencing machinery.

To test the effectiveness of the miDGE approach before applying it to PVs, we first focused on a single larger genome virus, the herpesvirus Japanese Macaque Rhadinovirus (JMRV). JMRV is a gamma-2 herpesvirus with genomic sequence similar to the highly related Rhesus Rhadinovirus (RRV). When we initiated these studies, it was not yet known if JMRV encoded miRNAs, although this would be expected since numerous precursor miRNAs (pre-miRNAs) had already been identified in RRV, which shares high sequence similarity with JMRV [46]. Indeed, work from Skalsky *et al*. has now identified 15 novel viral miRNA encoded by JMRV [46]. Thus, JMRV serves as a proof-of-principle “test genomic space” to evaluate miDGE.

**Fig 2:**
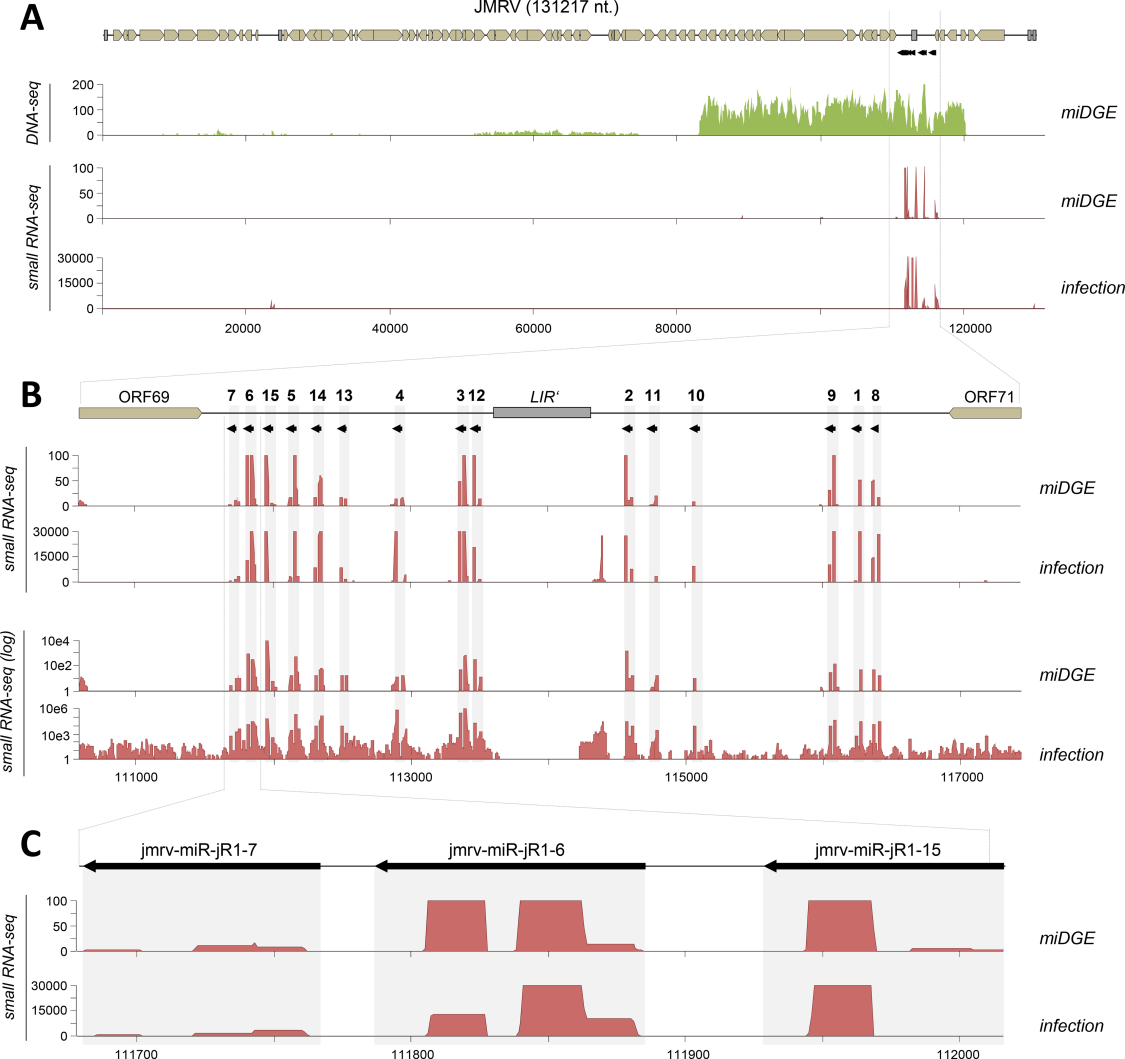
Validation of miDGE via confirmation of miRNAs encoded by Japanese Monkey Herpesvirus (JMHV). **(A)** *Top*: Coverage plot of DNA-seq of expression fragment library containing a cosmid with ~40kb of the Japanese Monkey Herpesvirus (JMRV) genome. *Center and bottom*: small RNA coverage plots mapping of small RNA-seq reads from HEK293T cells transfected with the expression library (*center*) or from JMRV-infected rhesus primary fibroblasts (*bottom*). **(B)** Small RNA-seq coverage plot in linear (top panels) or logarithmic (bottom panels) across the miRNA-coding region. The genomic location of the 15 JMRV pre-miRNAs is indicated by black arrows at the top. **(C)** Detailed depiction of small RNA-seq coverage across the first three pre-miRNAs of the JMRV miRNA cluster.

A cosmid with an approximately 36 kilobase (kB) region of the JMRV genome that encompassed the region with positional homology to the RRV miRNA cluster was fragmented by sonication and used to construct a miDGE expression library. DNA-seq analysis confirmed that the library covered the entire region carried by the original cosmid construct (Fig 2A, top panel). We then transfected our library into HEK293T cells and analyzed small RNA (sRNA) expression by sRNA-seq. In parallel, we also performed small RNA-sequencing from fibroblasts that had been infected with JMRV *in vitro*. As shown in Table 1, approximately 13% of all reads from infected fibroblasts mapped to viral genomes, consistent with the fact that JMRV establishes a productive infection and replicates to high titers in such cultures. As expected, the relative fraction of viral reads was much smaller (< 0.1%) in cultures transfected with the miDGE library. In both cases, the bulk of host reads originated from bona fide small RNA species, indicating no or little contamination of our small RNA preparation by degradation or breakdown products of longer RNA molecules. As shown in Fig 2A and 2B, small RNA-seq reads mapped across the JMRV genome in patterns that were similar between the miDGE and infection experiments, with the great majority of reads (96% and 88% of viral reads, respectively; see Table 1, S1 Table) aligning to the genomic location of the 15 pre-miRNAs previously identified by Skalsky and colleagues. Comparison of coverage profiles across individual pre-miRNAs furthermore suggested that most precursors were processed to produce ratios of mature 5p- and 3p-species that were likewise similar (S1 Fig) between miDGE and infection experiments. In addition to miRNA reads, infected cells produced a profile of low level seemingly randomly scattered reads (see log-scale plots in Fig 2B), likely originating from breakdown products of viral mRNAs in lytically infected cells. Such background was substantially lower in our miDGE experiments. The data were next used to perform a *de-novo* prediction of pre-miRNAs with the miRDeep2 algorithm [47], providing the pipeline only with an annotated reference set of known host (*i*.*e*., human) miRNAs deposited in miRBase v21, but not any miRNAs of viral origin. When processed with the sRNA-seq data from the miDGE library, this analysis readily identified 14 of the 15 known pre-miRNAs in JMRV (Table 2, S2 and S3 Datasets). Another two predictions mapped to the opposite strand of miRs-jR1-2 and -8, consistent with the previous observation that some herpesvirus pre-miRNAs can produce mature products when transcribed in the antisense orientation [48]. As our approach is agnostic of transcriptional directionality in the parental viruses, it is plausible that such miRNAs will register in the analysis.

**Table 1:** Small RNA-seq read & mapping statistics.

	JMRV		PV
	infection ^a^	miDGE ^b^	miDGE ^c^
**mapped reads:**^**d**^	**24,971,581**	**37,708,870**	**308,253,850**
**viral reads:**^**e**^	**3,224,344 (12.91%)**	**14,728 (0.04%)**	**234,490 (0.08%)**
*-viral miRNA*: ^f^	*2*,*826*,*087 (87*.*65%)*	*14*,*074 (95*.*56%)*	181,533 (77.42%)
**host reads:**^**g**^	**21,747,237 (87.09%)**	**37,694,142 (99.96%)**	**308,019,360 (99.92%)**
*-miRNA*	*21*,*147*,*183 (97*.*24%)*	*21*,*817*,*569 (57*.*88%)*	261,297,348 (84.83%)
*-snoRNA*	*92*,*422 (0*.*42%)*	*3*,*249*,*936 (8*.*62%)*	13,086,009 (4.25%)
*-lincRNA*	*228*,*928 (1*.*05%)*	*4*,*982*,*222 (13*.*22%)*	7,198,273 (2.34%)
*-retained intron*	*23*,*569 (0*.*11%)*	*1*,*132*,*491 (3%)*	5,741,648 (1.86%)
*-ns*. *mediated decay*	*43*,*335 (0*.*2%)*	*2*,*279*,*698 (6*.*05%)*	3,905,681 (1.27%)
*rRNA*	*9*,*286 (0*.*04%)*	*411*,*783 (1*.*09%)*	3,037,595 (0.99%)
*-protein coding*	*40*,*240 (0*.*19%)*	*821*,*732 (2*.*18%)*	2,708,946 (0.88%)
*-tRNA*	*3*,*870 (0*.*02%)*	*538*,*650 (1*.*43%)*	2,587,863 (0.84%)
*-misc*. *RNA*	*8*,*335 (0*.*04%)*	*289*,*807 (0*.*77%)*	2,539,257 (0.82%)
*-processed transcript*	*50*,*601 (0*.*23%)*	*282*,*498 (0*.*75%)*	1,249,588 (0.41%)
*-other*	*94*,*589 (0*.*43%)*	*1*,*846*,*576 (4*.*9%)*	4,392,203 (1.43%)

a: reads mapped to viral precursor or mature miRNA regions in JMRV-infected fibroblasts

b,c: reads mapped to viral precursor or mature miRNA regions in 293T cells transfected with the JMRV or miDGE expression libraries, respectively

d: sum of reads mapped to either viral genomes or host transcriptome

e: reads mapped to JMRV or PV genomes present in the miDGE library. Percentages indicates the relative fraction of viral among total reads.

f: reads mapped to viral miRNA regions. Percentages indicates the relative fraction of viral miRNAs among all viral reads.

g: Viral reads mapped to the host transcriptome. Percentages (in bold) indicates the relative fraction of host reads among total reads. Subsequent lines show the number of reads mapped to different host RNA host species and the fraction relative to all host reads.

**Table 2:** JMRV pre-miRNA predictions.

miDGE pre-miRNA predictions				
coordinates^a^	score^b^	confidence^c^	reads^d^	matched pre-mir^e^
111946..112004 (-)	4923.3	98+/-4%	9,654	jmrv-miR-jR1-15
111806..111862 (-)	570.9	98+/-4%	1,117	jmrv-miR-jR1-6
113350..113408 (-)	337.8	98+/-4%	660	jmrv-miR-jR1-3
113455..113515 (-)	154.5	98+/-4%	300	jmrv-miR-jR1-12
116039..116097 (-)	75.5	98+/-4%	152	jmrv-miR-jR1-9
116362..116424 (-)	29.7	98+/-4%	55	jmrv-miR-jR1-8
112484..112543 (-)	17.8	98+/-4%	32	jmrv-miR-jR1-13
112884..112956 (-)	16	98+/-4%	28	jmrv-miR-jR1-4
114744..114800 (-)	10.9	98+/-4%	18	jmrv-miR-jR1-11
114562..114625 (-)	6.1	99+/-4%	9	jmrv-miR-jR1-2 (as) ^f^
116365..116426 (-)	5.6	98+/-4%	7	jmrv-miR-jR1-8 (as)
116234..116288 (-)	2.8	95+/-6%	40	jmrv-miR-jR1-1
114560..114602 (-)	2.5	95+/-6%	1,441	jmrv-miR-jR1-2
112296..112359 (-)	2.4	95+/-6%	76	jmrv-miR-jR1-14
112115..112177 (-)	2.3	95+/-6%	492	jmrv-miR-jR1-5
115052..115109 (-)	1.1	94+/-6%	9	jmrv-miR-jR1-10

a: coordinates of pre-miRNA sequence in the JMRV reference genome

b: log-odds score for the prediction representing a true positive

c: confidence value for the prediction representing a true positive

d: reads aligned to the candidate pre-miRNA sequence by the miRDeep2 mapper

e: known JMRV miRNAs that map to the location of miRDeep2 predictions

f: “as” denotes predictions that were made for the antisense strand of known miRNAs

A single JMRV miRNA, miR-jR1-7, evaded detection by miDGE. Inspection of sequencing and mapping data revealed that this was not due to the principal absence of miRNA products, given that 14 reads had been correctly mapped to mature miR-jR1-7 species (see S1 Table and coverage profiles in Figs 2B, 2C and S1). While this number is relatively low, miRs-jR1-10 and -11 were accurately identified despite read counts that were in a similar range (see Table 2). Indeed, miRDeep2 analysis of small RNAs from JMRV-infected cells also failed to identify miR-jR1-7, even though more than 2000 reads had mapped to mature species from this miRNA (S1 Table and Figs 2B, 2C and S1). Although we do not know the precise reason for the pipeline’s inability to predict miR-jR1-7, we suspect that the close proximity of miRs-jR1-7 and -6 may have interfered with delineation of candidate precursor sequences that are subjected to structure prediction. Given the above, we conclude that, at least in the context of a herpesviral genome and within the detection limits set by the bioinformatic prediction pipeline, miDGE can successfully identify bona fide miRNAs at sensitivity levels that are on par with those observed in an authentic infection system.

### Identification of candidate PV miRNAs in non-high risk PVs

**Fig 3:**
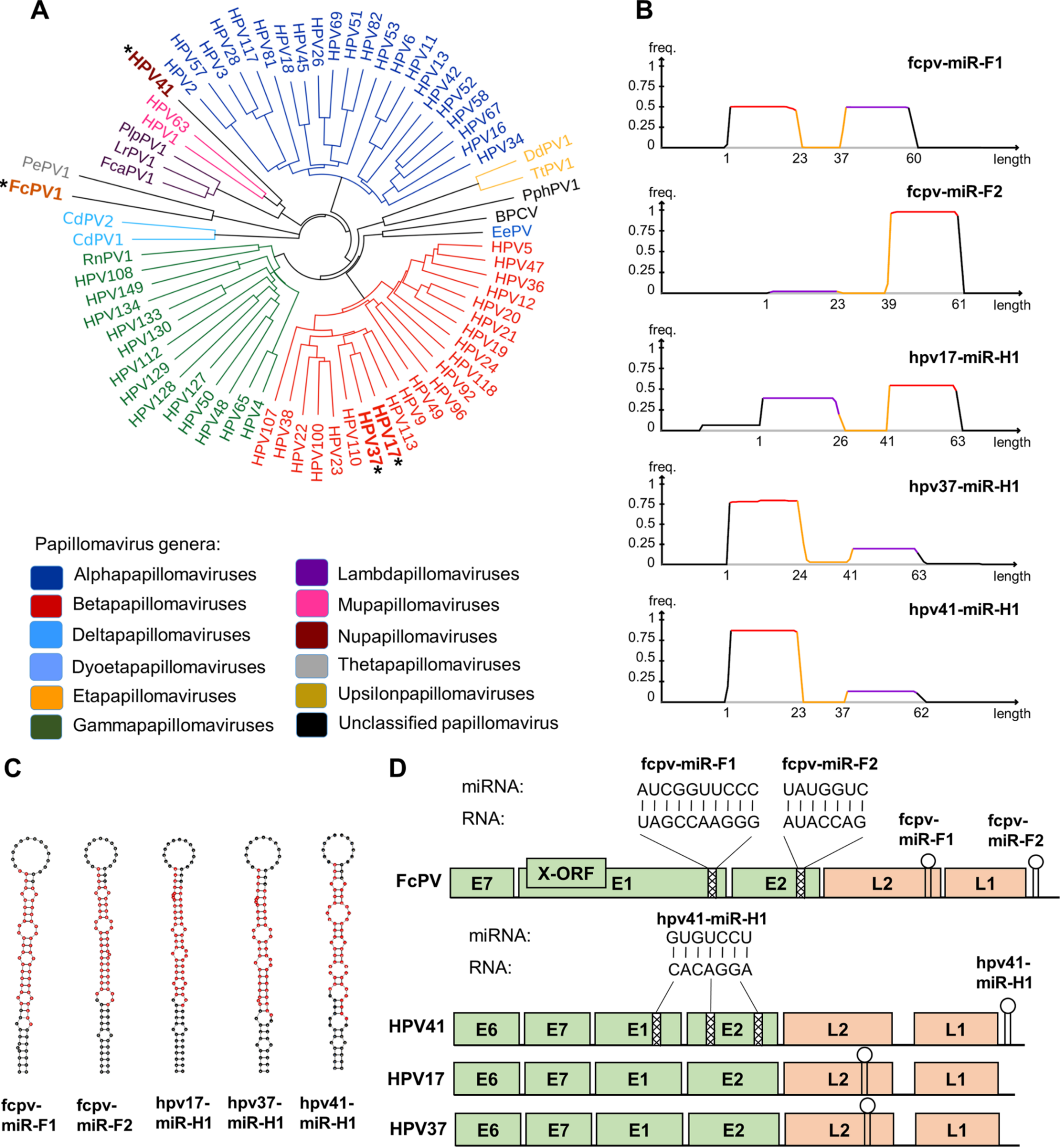
miDGE identifies PV-encoded miRNA candidates. **(A)** Neighbor-joining tree calculated on alignment of L1 nucleotide sequences of the better-covered papillomaviruses (n = 73 with >95% DNA coverage) included in miDGE library. Color indicates genus membership, with miRNA-encoding papillomaviruses in bold and select high risk cancer-associated papillomaviruses in italics. **(B)** Small RNA coverage distribution of top-scoring miDGE miRNA candidates that were predicted by MiRDeep2. **(C)** Structures of PV pre-miRNAs were predicted by minimal free energy folding using the RNAfold algorithm. The positions of mature miRNAs observed in small RNA-seq libraries are indicated in red. **(D)** The position of identified viral pre-miRNAs is denoted by the hairpin shape. The identified seed sequence matches are noted at their respective positions with the sequences of the miRNA and potential targets.

Our goal was to screen numerous PV genomes representing diverse clades in the PV family for viral miRNAs. To accomplish this, we collected 113 cloned PV genomes from both human and non-human animal sources (S2 Table). Additionally we included two polyomaviruses (Simian Virus (SV40) and Merkel cell polyomavirus (MCPyV)) known to express viral miRNAs [23,49], as positive controls in our PV library. Since the PV/PyV constructs were considerably smaller than our JMRV cosmid and thus potentially less sensitive to mechanical shearing, we utilized three different 4-base pair cutter restriction enzymes in addition to the sonication procedure to fractionate the viral DNA (see material and methods for details). High throughput Illumina DNA sequencing revealed that we had high coverage (S2 Table, S1 Dataset) of numerous genomes. A total of 73 PV genomes had greater than 95% coverage (see S2 Table). As shown in Fig 3A, this set contained representatives from the majority of known PV clades. We next conducted high throughput sequencing of small RNAs from cells transfected with the PV libraries. As expected based on our previous analysis of the JMRV library, we again observed that only a relatively small fraction (0.08%) of reads mapped to viral genomes (Table 1, right column). Likewise, the majority of host reads were derived from bona fide small RNA species, indicating that the RNA preparations were largely free of contaminating RNA degradation products.

We next applied our computational methods based on miRDeep2 to identify small RNA reads consistent with bona fide miRNAs. The algorithmic approach prioritizes those small RNAs of appropriate size (between 17–24 nucleotides) that had a read distribution as being plausibly derived from a pre-miRNA hairpin. This analysis identifies signatures of bona fide miRNAs including the presence of a predicted stem loop structure and clearly defined 5’ ends as processed by Dicer/Drosha. Typical read density coverage plots of such signatures exhibit two "plateaus", where each plateau represents a miRNA derivative from either the 5' or 3' arm of a pre-miRNA hairpin (5p or 3p miRNA, respectively) (see, for example, Figs 2C and 3B). In between each plateau is a trough in density coverage where the terminal loop portion of the pre-miRNA is under-represented in our libraries due to terminal loops of processed pre-miRNAs not being stabilized in RISC as miRNA derivatives are. miDGE readily called miRNAs from the positive control polyomavirus genomes included in the libraries.

The vast majority of the greater than 660,000 combined nucleotide PV genomic space covered did not produce miRNA candidates, consistent with a low false positive rate for miDGE. However, miDGE did call five high-scoring miRNA candidates which, like our SV40 and MCPyV positive controls, were awarded confidence levels of >95% by the miRDeep2 prediction algorithm (Table 3 and Fig 3B–3D). These five high-scoring candidates originated from four different PV genomes (one each from HPV41, HPV17, HPV37, and two from FcPV1). MiRDeep2 called an additional 7 candidates with lower scores and/or confidence levels (see S2 and S3 Datasets for all PV predictions made by the pipeline). One of these mapped to the FcPV1 genome, *i*.*e*., the same genome which had also produced two high scoring candidates. We noted that the read sequences for another three of the lower scoring predictions (HPV types 5, 49 and 105; marked ‘l.c.’ in S2 Dataset) were of low complexity. Indeed, applying an entropy-based filter (see Material and Methods) to remove low-complexity reads prior to our bioinformatic analysis eliminated these predictions (but none of the other candidates). We thus deem it likely that the predictions made for HPVs 5, 49 and 105 result from cross-mapping of reads that may originate from repetitive regions. The predicted hairpin structures (S4 Dataset) of the remaining lower-scoring candidates have features that are unusual for canonical miRNAs, for example a very short distance (2 to 4 nucleotides) between 5p and 3p reads (HPV113 and HPV3) or a 5p read that overlaps with much of the predicted terminal loop structure (PePV). We therefore suspect that these candidates are likewise false positives. However, as we have not attempted to experimentally verify these three predictions, we cannot exclude the possibility that one (or more) of them may represent authentic miRNAs.

**Table 3:** High-scoring PV pre-miRNA predictions.

miDGE pre-miRNA predictions					
genome	coordinates^a^	score^b^	confidence^c^	reads^d^	pre-miR^e^
FcPV1	4989..5048 (-)	3,541.5	96+/-8%	6,952	fcpv-miR-F1
FcPV1	7092..7152 (-)	676.9	96+/-8%	1,325	fcpv-miR-F2
HPV37	4558..4620 (-)	106.3	96+/-8%	207	hpv37-miR-H1
HPV41	7110..7171 (-)	50.5	96+/-8%	99	hpv41-miR-H1
HPV17	4560..4622 (-)	22.7	96+/-8%	43	hpv17-miR-H1
SV40	2232..2290 (-)	79,653	96+/-7%	156,242	sv40-mir-S1
MCPyV	3882..3937 (-)	7,811.6	96+/-7%	15,320	mcv-mir-M1

a: coordinates of pre-miRNA sequence in the reference genome

b: log-odds score for the prediction representing a true positive

c: confidence value for the prediction representing a true positive

d: reads aligned to the candidate pre-miRNA sequence by the miRDeep2 mapper

e: designation of novel PV pre-miRNAs (FcPV1, HPV17,HPV41, HPV37), or known PyV pre-miRNAs pre-miRNAs that map to the location of miRDeep2 predictions

**Fig 4:**
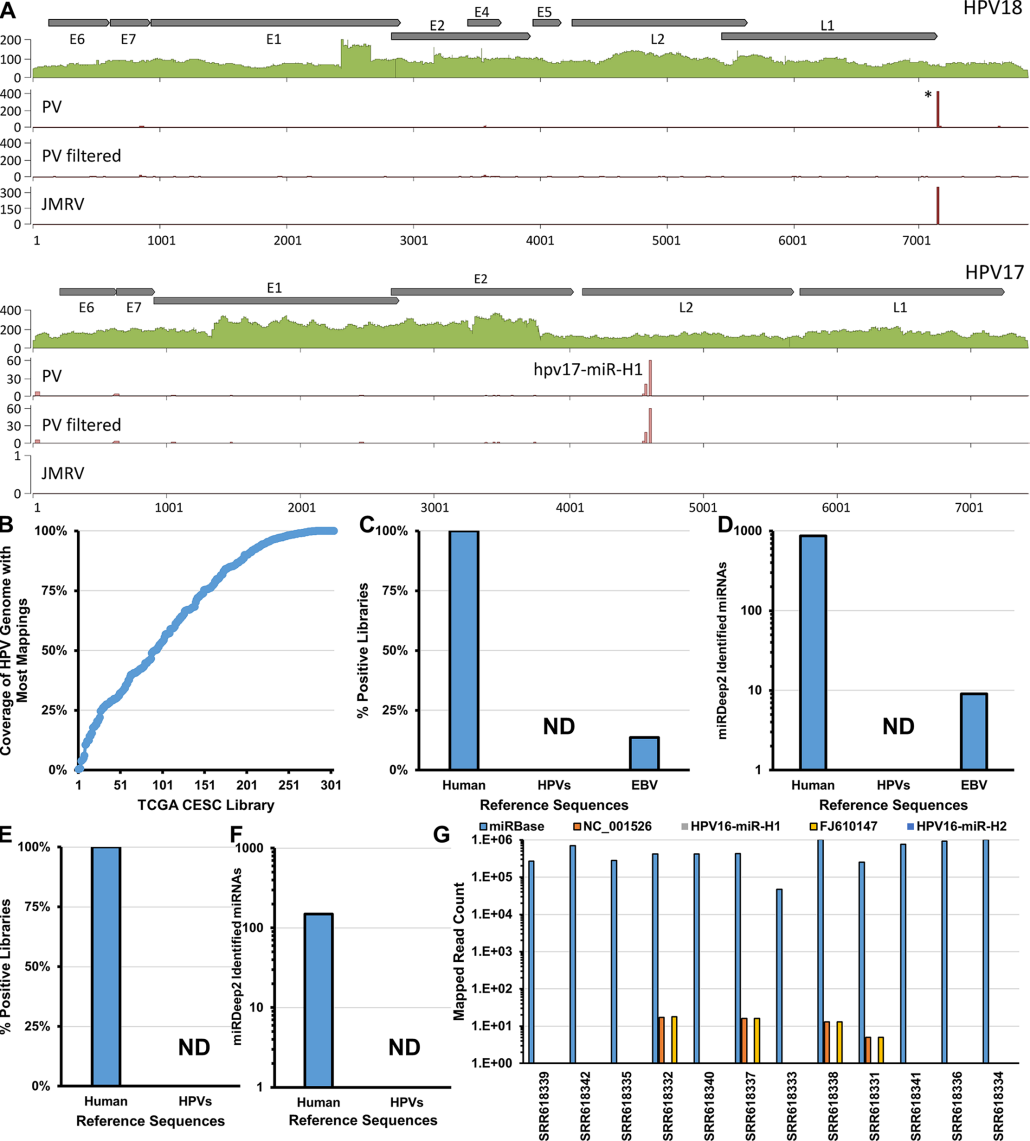
High Risk HPVs do not encode canonical microRNAs. **(A)** Coverage plots of miDGE DNA-seq and small RNA-seq across the genomes of HPV18 and HPV17. Filled green plots at the top of each panel show DNA-seq coverage, the three plots underneath show mapped small RNA-seq from: *PV*: HEK293T-cells transfected with our papillomavirus miDGE library, *PV filtered*: same reads as in PV, but filtered to eliminate low-complexity reads *JMRV*: Serving as a negative control, derived from 293T-cells transfected with our JMRV miDGE library. JMRV read counts were normalized to correct for different sequencing depths between PV and JMRV miDGE experiments (see total read counts in Table 1). Asterisk indicates a previously purported miRNA candidate region suggested in the literature [39], which is nonspecific (detected in the negative control JMRV miDGE analysis, lower plot) and can be eliminated by removing low complexity reads (center plot). **(B)** RNA-seq coverage for the most abundantly mapped HPV in 303 tumors in the TCGA CESC project [50]. Each of the 303 libraries are represented on the X-axis (sorted based on Y-axis value). Y-axis indicates the percentage of the positions in the HPV genome with read mappings. Libraries with > = 50% coverage (213 libraries) were used for subsequent analysis. **(C)** Percentages of TCGA cervical squamous cell carcinoma (CESC) libraries with miRDeep2 miRNA identifications for each set of reference sequences. Number of libraries examined is 213. **(D)** Number of unique miRDeep2 miRNA identifications across TCGA CESC libraries for each set of reference sequences. Number of libraries examined is 213. **(E)** Percentages of Qian *et al*. [40] libraries with miRDeep2 miRNA identifications for each set of reference sequences. Number of libraries examined is 12. **(F)** Number of unique miRDeep2 miRNA identifications across Qian *et al*. libraries for each set of reference sequences. Number of libraries examined is 12. **(G)** Raw read counts of all small RNAs mapping to the indicated reference sequences for each library from Qian *et al*.

Our bioinformatic analysis did not predict any candidates for the negative strand of papillomaviruses. Likewise, although their genomes were fully covered in our library, no predictions were made for any of the plus- or minus-strand miRNAs previously suggested for HPV types 6, 16, 18, 38 or 45 [38–40]. While highly unlikely, we nevertheless considered it formally possible that our bioinformatic prediction pipeline had missed all 9 purported candidates in these genomes. Therefore, we inspected small RNA read coverage across the respective genomic regions. As shown in S2 Fig, our high confidence PV candidates and the PyV positive controls exhibited the typical coverage profiles of bona fide miRNAs. In contrast, only few reads mapped to the previously suggested papillomavirus pre-miRNA regions in HPV types 6, 16, 38 and 45 (S3A Fig), and these reads furthermore did not match the distribution as expected for mature miRNAs being derived from the purported hairpin precursors (S3 Fig). While the absence of read coverage argues against the existence of miRNAs in above HPV species, for one of the purported miRNAs (HPV18- LCR), we indeed observed a strong signal in our miDGE analysis (Fig 4A, top panel; see also S3B Fig for a detailed depiction of the covered region). However, we also noticed that the mature miRNA sequences that supposedly derive from this region of the viral genome [39] are of very low complexity (trinucleotide entropy < 63). In agreement with this observation, when we employed the same complexity filter as used for our miRDeep2-predicted candidates, the peak was completely eliminated (Fig 4A, panel labeled ‘PV filtered’). Given this and the absence of read coverage in adjoining regions of the viral genome, we suspected that the signal originated from cross-mapping host RNAs. To investigate this hypothesis, we mapped the small RNA-reads from our JMRV experiment to the PV genomes. Indeed, as shown in the panel labeled ‘JMRV’ of Fig 4A, this dataset contains a largely identical set of sequences which produced a peak at the exact same location of the purported miRNA (see S4 Fig for an enlarged view of the peak), even though the JMRV miDGE library does not contain any PV sequences. In contrast, reads originating from all of our high scoring miRNA candidates in FcPV1, HPV17, HPV37 and HPV41 were not eliminated by complexity filters, and were furthermore highly specific for the PV libraries (shown exemplary for HPV17 in the lower panels of Fig 4A). Together, these results demonstrate the plausibility of using miDGE to identify miRNAs from complex multi-genome expression libraries and suggest that while some PVs may encode miRNAs, most PVs, including the high-risk types 16, 18, and 45, do not encode canonical miRNAs.

### High risk PV types do not encode canonical miRNAs

To further evaluate whether high risk HPVs code for miRNAs, we analyzed the large and small RNA transcriptomes of 303 cervical carcinomas that are part of The Cancer Genome Atlas (TCGA) [50]. We limited our subsequent analysis to 213 solid tumors that had both large and small RNA-seq datasets and at least 50% RNA coverage of a particular HPV genome in the large RNA-seq (Fig 4B). Importantly, we observed 42 samples that had >99% RNA coverage for one of the following HPVs: 16, 18, 31, 45, or 58. Because these samples displayed essentially complete RNA coverage throughout the entire relevant HPV genome, it would be expected that any putative miRNA encoded in these genomes would have the ability to be processed and detected in these samples. We applied the miRDeep2 pipeline to each small RNA library to identify putative miRNAs. We observed hundreds of miRBase annotated host miRNAs per library (255–454), but no HPV derived candidates (Fig 4C and 4D). However, we were able to identify Epstein-Barr virus (EBV) miRNAs in ~14% of the tumor samples, demonstrating that we are able to detect viral miRNAs that are present in only trace amounts, likely from EBV-infected infiltrating lymphocytes (Fig 4C). Thus, despite having transcripts that span the entire HPV viral genomes, tumors associated with high risk HPV types do not express canonical HPV-derived miRNAs. Qian *et al*. previously reported HPV16-derived viral miRNAs present in small RNA-seq libraries prepared from transformed cell lines as well as cervical tissue and cancer samples [40]. We re-analyzed their deposited small RNA transcriptomic datasets [40] for the expression of HPV derived small RNAs. Similar to the HPV-associated tumors in the TCGA, we observed many miRBase-annotated host miRNAs per library (50–100), but no HPV-derived candidates (Fig 4E and 4F). Also in contrast to Qian *et al*., we observe few reads mapping to either HPV16 reference sequence (< 20 total reads per library). Furthermore, we do not observe any reads for the purported HPV16 mature miRNAs in these datasets (Fig 3F). Inspection of the alignments presented by Qian *et al*. [40] suggests that an excessive allowance for sequencing errors and small nucleotide polymorphisms (SNPs) combined to produce false read mappings to the purported HPV16 miRNAs. Thus, our re-analysis suggests that the small RNAs described in their study may be artifacts of the bioinformatics analysis and do not represent actual small RNAs derived from HPV16. Combining these findings with our miDGE results (Figs 4A and S3), we conclude that it is highly unlikely that high risk HPVs 16, 18, 31, 45 and 58 express canonical viral-encoded miRNAs.

### Validation of PV miRNAs

**Fig 5:**
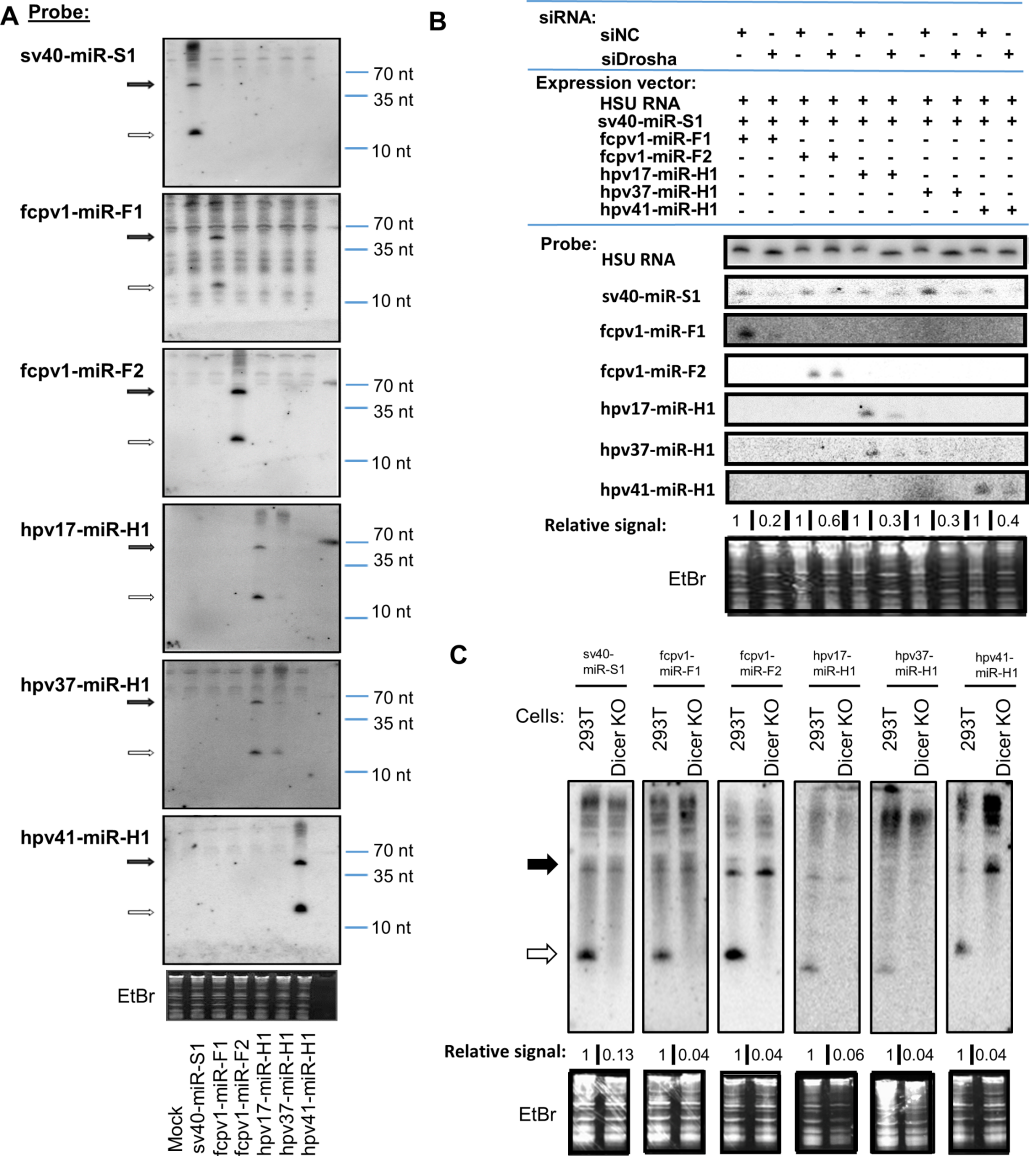
MiRNA candidates identified from miDGE analysis are detected by Northern blot. **(A)** Northern blot analysis of total RNA from HEK293T cells transfected with indicated miRNA or putative miRNA expression vectors, with ethidium bromide stained low molecular weight RNA shown as a load control. This figure represents a single membrane that was probed first for control SV40 miRNA, then stripped and re-probed for each of the indicated miRNAs. Solid and outline arrows on left side correspond to pre-miRNAs and mature miRNAs, respectively, and approximate sizes are noted on right side. **(B)** Northern blot analysis of total RNA from HEK293T cells transfected with anti-Drosha siRNA or negative control (NC) siRNA, then re-transfected after 48 hours with respective siRNAs and indicated putative miRNA expression vectors. Total RNA was harvested after 48 hours. Ethidium bromide stained low molecular weight RNA shown as a load control. This figure represents a single membrane that was probed first for control SV40 miRNA, then stripped and re-probed for each of the indicated miRNAs. Membrane was additionally probed for HSUR (Herpesvirus saimiri U RNA 4) as a transfection control. Normalized to HSUR RNA, all indicated miRNAs are detected at higher intensity in negative-control-treated cells than Drosha knockdown cells, suggesting canonical dependence on Drosha processing. The numbers below each blot correspond to the ratio of mature miRNA signal in control cells compared to mature miRNA signal in Drosha-knockdown cells after each signal has been normalized to load control HSUR signal. Note: due to sequence similarity between putative HPV-derived miRNAs, cross-reactivity is seen in hpv17-miR-H1 lanes and hpv37-miR-H1 lanes. **(C)** Northern blot analysis of RNA from either HEK293T or Dicer KO (NoDice [68]) cells transfected with the indicated miRNA expression vectors. Solid and outline arrows on left side correspond to pre-miRNAs and mature miRNAs, respectively. The numbers below each blot correspond to the ratio of mature miRNA signal to pre-miRNA signal, normalized to the same ratio in HEK293T cells. This figure represents a single membrane that was probed, then stripped and reprobed with the indicated oligonucleotide probes (SV40, FcPV1 F1 and F2, HPV41 miRNA probes) and a second membrane for HPV17 and HPV37 miRNAs. Ethidium bromide stained low molecular weight RNA is shown as a load control.

The burden of proof for establishing bona fide miRNAs includes evidence of specific processing by the miRNA machinery and silencing activity within RISC. To vet the five high-scoring PV candidate miRNAs, we first conducted northern blot analysis. Northern blot analysis can provide information about the size and processing of pre-miRNAs and derivative miRNAs. We cloned the candidate miRNA genes and flanking regions downstream of an RNP II promoter and transfected these plasmids into HEK293T cells. We harvested total RNA and conducted northern blot analysis. For each candidate, bands migrating at positions consistent with the appropriate size typical of miRNAs were observed. Additionally, on most blots, a clear band consistent with a pre-miRNA was also observable. We note that due to the high sequence similarity of the HPV 17 and 37 miRNAs (~91%), even though we confirmed the blots were completely stripped of specific signal, we consistently observe cross-reacting signal from both lanes when probed with either probe (Fig 5A & 5B). Overall, this analysis showed that all five high-scoring candidates gave rise to banding patterns consistent with canonically processed miRNAs (Fig 5A).

**Fig 6:**
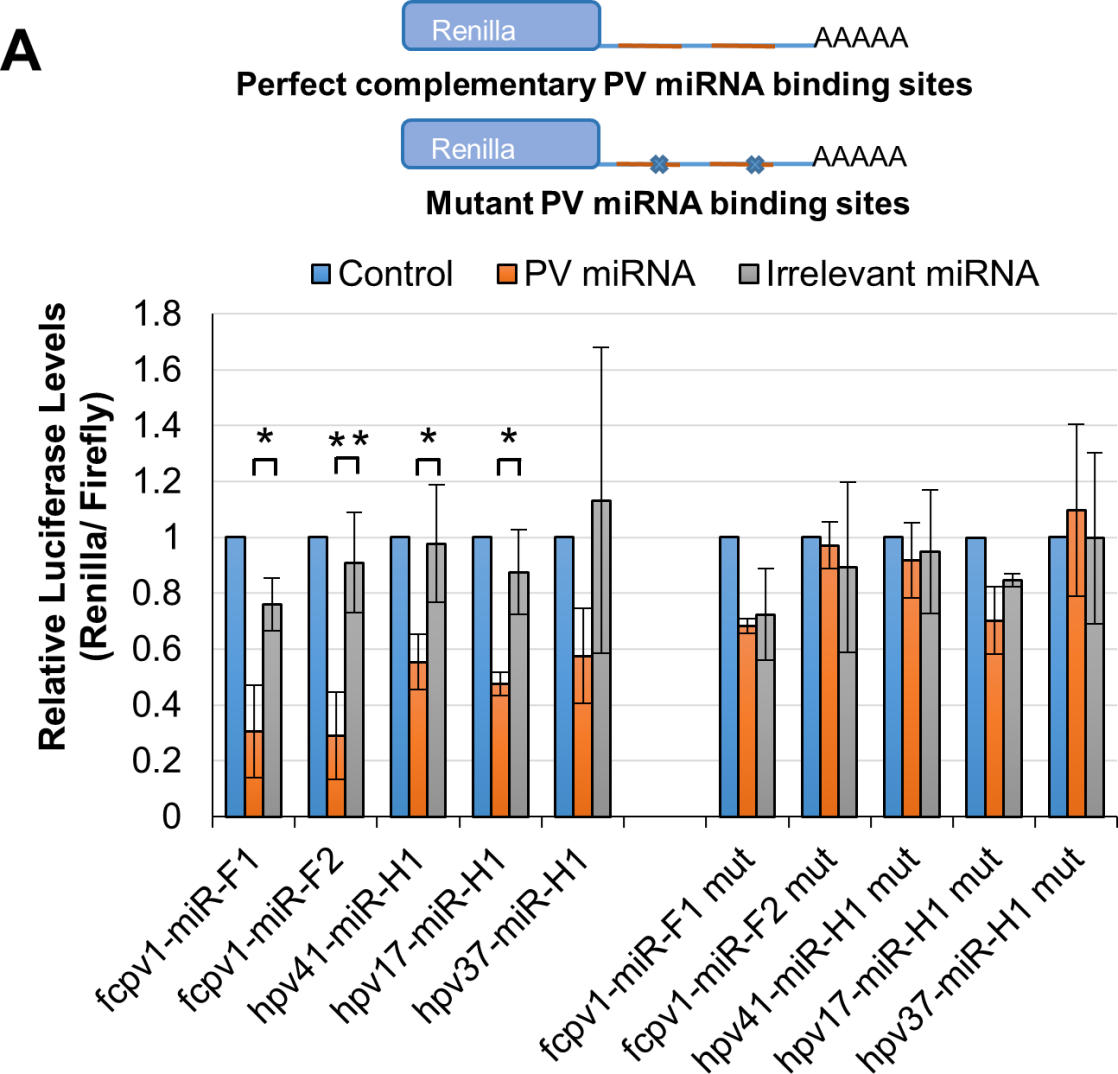
PV-encoded miRNAs are active in RISC. RISC reporter assays for the PV-encoded miRNAs where HEK293 cells were co-transfected with a firefly luciferase transfection control and *Renilla* luciferase reporter with either perfectly complementary sequence matches for each indicated miRNA, or its respective negative control seed complement mutant. Either of these were co-transfected with control empty miRNA expression vector (blue), the relevant PV miRNA-expression vector (orange), or negative control irrelevant miRNA expression vector (SV40) (gray). Average *Renilla* luciferase activity relative to firefly luciferase normalized to empty miRNA expression vector control is shown for fcpv1-miR-F1 (N = 3), fcpv1-miR-F2 (N = 5), hpv41-miR-H1 (N = 5), hpv17-miR-H1 (N = 3), and hpv37-miR-H1 (N = 4). Statistical test performed was a Two-Sample t Test. The average *Renilla* luciferase activity normalized to firefly luciferase activity is shown, error bars indicate Standard Error, and asterisks indicate statistical significance, (*) p≤0.05; (**) p≤0.01.

To further validate these candidate miRNAs, we investigated their biogenesis, activity and expression. To determine if the biogenesis of these candidates required canonical miRNA machinery, we transfected our candidate miRNA constructs into cells with Drosha levels knocked down or that had Dicer gene expression knocked out (Fig 5B and 5C). As expected, this analysis showed that positive control SV40 miRNAs were dependent on both Drosha and Dicer (Fig 5B and 5C, respectively). All five candidate PV miRNAs showed reduced expression upon knockdown of Drosha (Fig 5B) and reduced ratios of miRNA:pre-miRNA in the absence of Dicer (Fig 5C). These results conclusively demonstrate that the five high-scoring candidate PV miRNAs derive from canonical miRNA biogenesis. We next generated luciferase-based RISC reporters for each viral miRNA candidate to determine if they are active in RISC. Reporters contained two perfectly complementary sequences to respective miRNA candidates in the 3’ untranslated region of *Renilla* luciferase. Negative control reporters contained the same sequences, with three nucleotides in the miRNA seed complement site substituted to disrupt binding. Transfection of these reporters resulted in specific knockdown of luciferase in each of the five reporters when co-transfected with respective miRNA expression vectors, but not in negative control reporters (Fig 6). These results suggest that each of the identified miRNA/candidates undergo canonical processing and are active in RISC.

**Fig 7:**
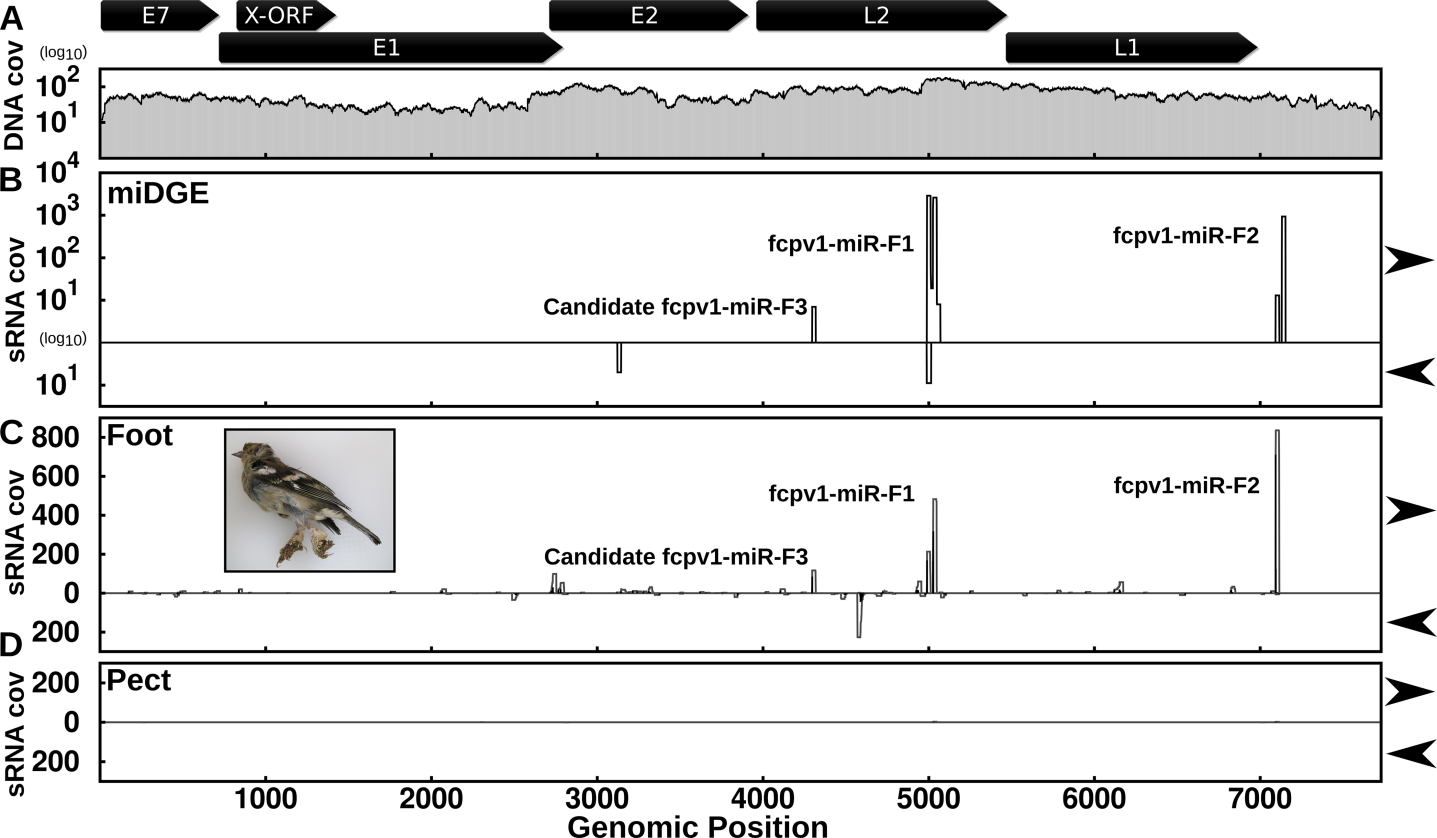
FcPV1 miRNAs are detected *in vivo* during viral infection. **(A)** DNA-seq coverage for FcPV1 genome in miDGE library is plotted on log10 scale on the y-axis. The x-axis corresponds to position in the genomic sequence. A schematic of the FcPV1 gene organization taken from NCBI reference sequence NC_004068 is provided at the top. **(B)** Small RNA-seq coverage for FcPV1 genome in miDGE library is plotted on log10 scale on the y-axis. Values above the x-axis correspond to the forward strand and those below correspond to the reverse strand. The x-axis corresponds to position in the genomic sequence. Peaks corresponding to the newly identified miRNA genes are labeled. **(C)** Plot of read coverage and start sites for reads mapping to the FcPV1 genome in library prepared with RNA from infected chaffinch leg lesion samples. On the y-axis, coverage is plotted with gray lines and read start counts are plotted with black impulses. Values above the x-axis represent the forward strand and those below represent reads mapping to the negative strand. Genomic position is indicated on the X-axis. Peaks corresponding to the newly identified miRNA genes (fcpv1-miRs-F1 & F2) are labeled as well as the lower expressed candidate miRNA “fcpv1-miR-F3”. The inset photograph is of one of the chaffinches with characteristic leg lesions used in preparation of the small RNA-seq libraries. **(D)** Plot of read coverage and start sites for reads mapping to the FcPV1 genome in library prepared with RNA from chaffinch pectoral muscle samples. On the y-axis, coverage is plotted with gray lines and read start counts are plotted with black impulses. Values above the x-axis represent the forward strand and those below represent reads mapping to the negative strand. Genomic position is indicated on the X-axis. Peaks corresponding to the newly identified miRNA genes are labeled.

A final criterion for confirmation of bona fide viral miRNAs includes detectable expression in infected cells or tissues. To date, we have been unable to obtain published or unpublished datasets to uncover samples infected with HPVs 17, 37 or 41. It would be informative in future studies to examine the expression/activity of these miRNAs if relevant human datasets or cell culture models become available. However, FcPV1 infection is recognized as a cause of macroscopic proliferative skin lesions affecting the legs and feet of chaffinches (*Fringilla coelebs*) with deeply fissured papillary growths (*i*.*e*., papilliferous), subsequently referred to here as ‘leg lesions’ [51] (Fig 7C inset). Therefore, we investigated the presence of candidate PV miRNAs in available leg lesion samples from wild chaffinches. We were able to obtain RNA from host tissue which tested positive for FcPV1 DNA by PCR [52]. We sampled both available leg lesion and apparently normal pectoral muscle tissue as a negative control from the same birds. We performed small RNA-seq profiling of the libraries and compared this with the data derived from our miDGE libraries (Fig 7A and 7B). Similar to our miDGE results, in the library prepared from leg lesions, we observed a pattern of reads mapping to the two confirmed pre-miRNAs (Fig 7B and 7C). Further consistent with miDGE, this analysis also identified a lower abundance, low-scoring third candidate miRNA (provisionally “candidate fcpv1-miR-F3”) in the L2 genomic region (Fig 7B and 7C). Other small RNAs mapped to the FcPV1 genome but these did not match a pattern consistent with miRNA biogenesis and likely represent nonspecific degradation of larger FcPV1 transcripts. In contrast, few read mappings were observed in the library prepared from negative control pectoral muscle (Fig 7D). Combined with the above biogenesis and activity data, these results demonstrate that FcPV1 infection gives rise to at least two bona fide PV miRNAs. Additionally, of the remaining PV miRNA candidates (HPVs 17, 37 & 41), we can say with confidence they are highly probable miRNAs, meeting all criteria of bona fide miRNAs with the exception of detection in infected cells. In keeping with the miRNA naming convention set forth by miRBase [53], we name these miRNAs/candidate miRNAs: fcpv1-miR-F1, fcpv1-miR-F2, hpv17-miR-H1, hpv37-miR-H1 and hpv41-miR-H1.

### PV miRNAs and select candidates can directly regulate transcripts corresponding to the early viral genomic region

**Fig 8:**
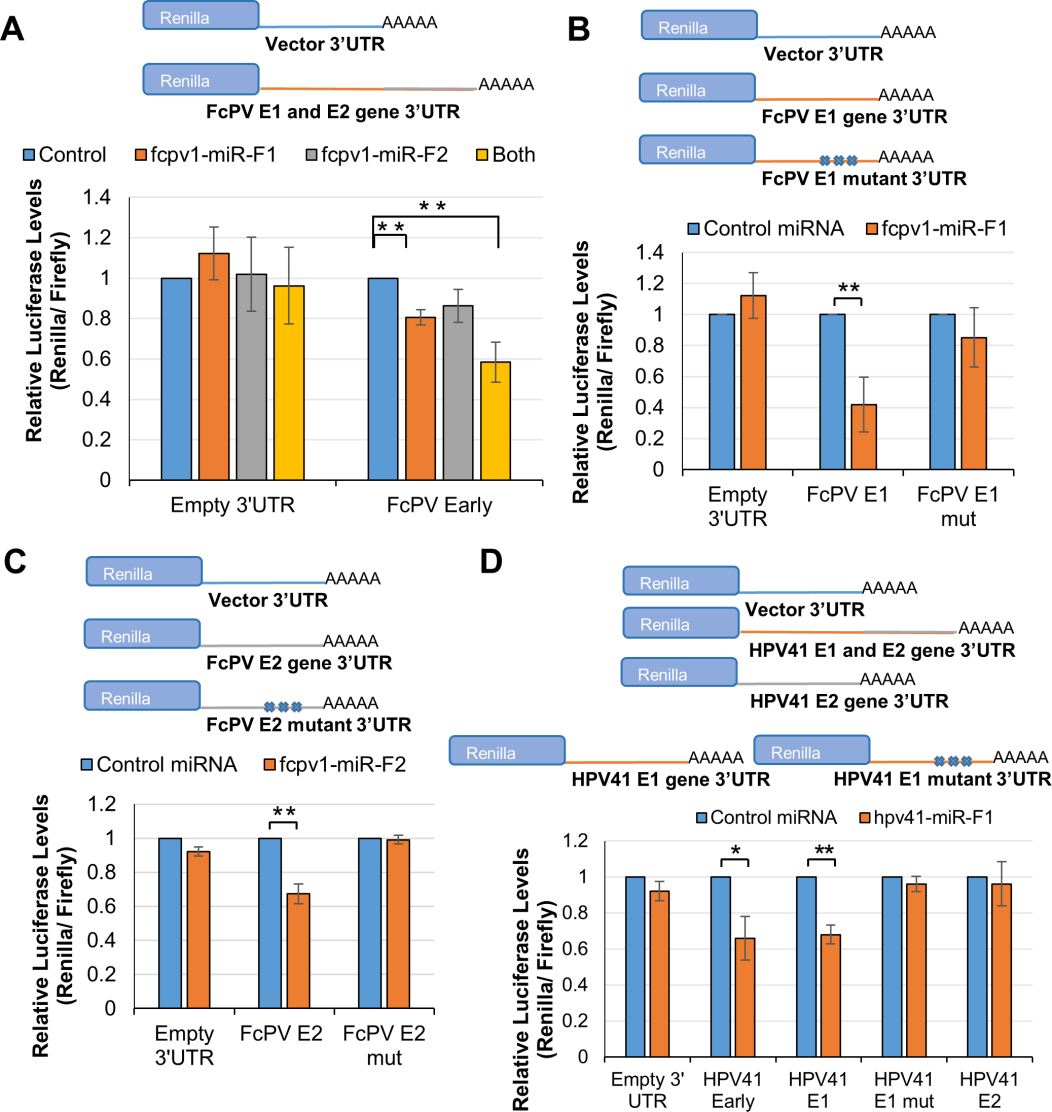
PV-encoded miRNAs can regulate transcript sequences in early genes. RISC reporter assays for the PV-encoded miRNAs. In all panels, the average *Renilla* luciferase activity normalized to firefly luciferase activity is shown, error bars indicate Standard Error, and asterisks indicate statistical significance, (*) p≤0.05; (**) p≤0.01. **(A)** HEK293T cells were co-transfected with either a control empty miRNA expression vector (control) or the indicated PV miRNA expression vector along with both the normalization control firefly luciferase vector and a *Renilla* luciferase-based reporter plasmids with vector UTR (Empty 3’UTR) or FcPV1 genomic DNA containing both putative miRNA docking sites (FcPV1 Early), N = 4. Statistical test performed was a One-Sample t Test. **(B)** HEK293T cells were co-transfected with either the SV40 miRNA expression vector (Control) or the indicated PV miRNA expression vector and both the control firefly luciferase vector and the *Renilla* luciferase-based reporter plasmids with vector UTR (Empty), FcPV1 E1 genomic sequence (E1), or the seed sequence mutant (E1 mut), N = 4. Statistical test performed was a One-Sample t Test. **(C)** HEK293T cells were co-transfected with either the SV40 miRNA expression vector (Control) or the indicated PV miRNA expression vector and both the control firefly luciferase vector and the *Renilla* luciferase-based reporter plasmids with vector UTR (Empty), FcPV1 E2 genomic sequence (E2), or the seed sequence mutant (E2 mut), N = 12. Statistical test performed was a One-Sample t Test. **(D)** HEK293T cells were co-transfected with either the SV40 miRNA expression vector (Control) or the HPV41 miRNA expression vector and both the control firefly luciferase vector and the *Renilla* luciferase-based reporter plasmids with vector UTR (Empty), HPV41 genomic DNA containing both putative miRNA sites (HPV41 Early), the site in E1 (HPV41 E1), the seed sequence mutant (E1 mut) N = 7 or the site in E2 (HPV41 E2), N = 4. Statistical test performed was a One-Sample t Test.

Previous studies in the small DNA virus polyomavirus (PyV) family demonstrated that PyV miRNAs directly regulate early viral transcripts [19,54,55]. Further, bandicoot papillomatosis carcinomatosis viruses (BPCVs), that have hybrid PyV-like early genes and genomic organization but PV-like capsid genes, also regulate early viral transcripts via viral miRNAs [56]. Therefore, we performed bioinformatic analysis to examine the possibility that PV miRNAs could regulate early viral gene expression. To identify putative viral target transcripts, we identified seed complementary sites of 7 or more nucleotides for each PV miRNA in its respective genome. This analysis revealed candidate target sites for derivatives from each of the five high confidence PV pre-miRNAs (S3 Table). Notably, both FcPV1 and HPV41 had candidate viral miRNA docking sites within the E1/E2 regions of their genomes, which has previously been demonstrated to include transcripts regulated by a host miRNA for HPV31 [25] (Fig 3D). We therefore tested these possible PV mRNA docking sites by engineering chimeric luciferase reporters containing either the entire E1/E2 region (termed “Early”) or sub-portions of the E1 or E2 genetic region encompassing a single predicted site of each respective genome (Fig 8). Co-transfection of the “Early” reporter plasmids with individual PV miRNA expression vectors revealed that “Early” reporters for the FCPV1 and HPV41 genomes display significantly less expression in the presence of their respective viral miRNAs (Fig 8). Co-transfection of FcPV1 miR-F1 alone reduced expression of the FcPV1 E1/E2 reporter and this regulation was enhanced by co-transfecting plasmids expressing both FcPV1 miRs-F1 and F2 (Fig 8A). As expected, co-transfection of the respective viral miRNA with the predicted single site reporters demonstrated a significant reduction in luciferase expression. Importantly, negative control reporters containing nucleotide mutations in the seed complement region of each predicted PV miRNA docking site alleviated the repression that we observed. When the same experimental setup was performed with the HPV41 miRNA and reporters, we observed similar results (Fig 8D). Co-transfection of the HPV41 E1/E2 reporter and miRNA expression vectors demonstrated a significant reduction in luciferase expression. Co-transfection of the HPV41 E1 reporter, but not the E2 reporter, resulted in a similar reduction as the full E1/E2 genomic region reporter, suggesting the E1 region contains the most relevant miRNA docking site. In contrast, the negative control HPV41 E1 mutant reporter was not regulated in response to miRNA expression vector transfection (Fig 8B–8D). These results demonstrate that FcPV1 and HPV41 miRNAs/candidates are able to directly regulate transcripts corresponding to the PV early genomic region.

## Discussion

Members of diverse virus families express miRNAs [4,5,7,30]. These include the herpesviruses, polyomaviruses, anelloviruses, and retroviruses [6,21,31,32]. Notably, all of these viruses undergo persistent infection, have access to the nucleus where key miRNA processing machinery resides and are exclusively DNA viruses or have a DNA component to their lifecycle. Viruses with a persistent component to their life cycle may especially benefit from the typically subtle regulation afforded by miRNAs. Based on these characteristics, at least some members of the PV family would be expected to encode miRNAs. Yet, until now, no widely accepted PV miRNAs are known. Here we report the first high confidence papillomavirus-encoded miRNAs from a minor subset of PVs.

We identify PV miRNAs from chaffinch leg lesions and highly probable miRNAs from the human PVs 17, 37, and 41. We designate the latter as “highly probable” because although they passed stringent cell culture-based criteria, due to a lack of relevant samples, we have not yet verified their existence *in vivo*. All five display the hallmarks of canonical miRNAs (Fig 5), including being processed by Dicer and Drosha and being highly active in RISC [8]. These RNAs derive from three divergent clades of PVs. HPVs 17 and 37 are in the beta clade, some members of which have been proposed to have a role in skin cancers [2]. The pre-miRNA hairpin region of the L2 locus for these viruses appears to have evolved in a common ancestor of these viruses, and may be shared with closely related HPVs 15 & 80. Sequence-structure alignments suggest the existence of a highly conserved hairpin in these viruses. However, since HPVs 80 and 15 were either not included (HPV80) or only very poorly covered (HPV15) in our library, additional experiments will be required to determine whether they indeed produce conserved miRNAs. HPV41 is notable in that it is the sole member of the Nu clade and is one of the few PVs that have starkly different locations in a PV family phylogenetic tree, depending if the tree is built upon the late (L1) or early proteins (E1). This implies HPV41 is likely a hybrid virus that arose from recombination [57] and may help to explain its atypical ability to encode a miRNA. FcPV1 is only distantly related to human PVs but its association with highly keratinized hyperplastic lesions allowed us to obtain RNA from PV-associated diseased chaffinch tissue. This confirmed that FcPV1 miRNAs are expressed *in vivo* (Fig 7) and further confirmed their status as *bona fide* viral miRNAs.

The HPV41 miRNA and one of the FcPV1 pre-miRNAs are found in non-protein-coding genomic locations, a common feature of most miRNAs. Notably, one of the FcPV1 miRNA loci, fcpv1-miR-F2, and the HPV41 miRNA locus are both found in similar locations downstream of the late genes past the likely late polyadenylation signal sequence. In contrast, the HPV17 and 37 miRNAs, as well as the other abundant FcPV1 miRNA (as well as the low abundance FcPV1 candidate), are found in a similar genomic region overlapping and in the same transcriptional orientation as the L2 locus (Fig 3D). Although these miRNAs could derive from an intronic primary transcript, it is nonetheless atypical for a pre-miRNA gene to overlap a protein-coding gene. Similar genomic arrangements are observed for miR-BHRF1 and the EBNA-LP gene in Epstein Barr Virus [58] and miR-K12 and the Kaposin gene in KSHV [48,59]. For KSHV, Drosha can suppress Kaposin expression in latent infection, but its steady state levels and consequent ability to regulate Kaposin levels decrease during stress and at late times of lytic infection [60,61]. Therefore, it is conceivable that FcPV1 and HPVs 17 & 37 similarly utilize Drosha to aid in controlling the expression of L2. HPV17 and 37 are the only PV miRNAs/candidates that share a high degree of sequence identity (91% 3P, 91% 5P), consistent with them being derived from virus types that are closely related. Except for HPVs17 and 37, there is no obvious relationship between the miRNA-positive PVs that might account for why they would preferentially encode miRNAs.

miDGE covered at least 698,000 nucleotides of sequence space (the sum of viral genome sequences covered by our JMRV and PV libraries) and called only few candidates other than the positive controls and the five high-confidence PV miRNAs that we validated. Therefore, we conclude that miDGE has an intrinsic low false positive rate. These findings are consistent with the current understanding that pre-miRNA hairpins have specific structural features that are required for efficient processing [62–64]. It should be noted, though, that since miDGE forces ectopic expression of genomic sequences, its predictions require independent validation to ensure that potential candidates are also expressed by authentic viral transcriptional units.

Compared to false positive rates, it is more difficult to estimate the frequency of false negatives in our approach. Generally, our analysis of JMRV suggests that the majority of bona-fide miRNAs are readily identified by miDGE. The sole JMRV miRNA that was missed by miDGE also evaded detection when we analyzed material from infected fibroblasts, demonstrating failure to identify jmrv-miR-jR1-7 was due to limitations of the miRDeep2 analysis pipeline rather than the miDGE protocol itself. The miRDeep2 algorithm identifies pre-miRNA candidates based on expected read coverage profiles produced by mature 5p- and 3p-miRNA species. These profiles are used to “excise” sequences for prediction of potential pre-miRNA hairpin structures. Based on the inspection of candidate sequences analyzed by the pipeline, we suspect that the close proximity of clustered miRNAs in the JMRV genome had led to an inaccurate excision of the jmrv-miR-jR1-7 precursor. As closely clustered miRNAs are a typical feature of herpesvirus genomes, such limitations should not severely impede our ability to identify PV miRNAs. Instead, we consider it more likely that some bona fide miRNAs in the 113 PV types that we strived to analyze may have evaded detection due to incomplete coverage of their genomes. In fact, only a subset of PV genomes (n = 63) had near-complete (>99%) coverage in our DNA libraries. Therefore, we can only make negative conclusions on this limited set of PVs. Our results show that 59/63 PV genomes with near-complete DNA coverage lack the ability to efficiently give rise to canonical miRNAs. This list includes high-risk HPVs such as HPV16 which our further transcriptomic analysis demonstrated does not encode miRNAs in tumors or cancer cell line settings (Fig 4A–4G). Although we acknowledge that our findings are skewed toward the alpha clade human PVs, we conclude that numerous and diverse PVs lack the ability to encode their own miRNAs.

What are the functions of the PV miRNAs? Our bioinformatic analysis and reporter assays (Figs 4A–4G and 8A–8D) suggest that one function of FcPV and HPV41 miRNAs is to regulate viral gene expression. This notion is consistent with the known function of other viral miRNAs, especially those derived from the PyVs and BPCVs [23,56]. Although we did not predict high confidence docking sites in the E1/E2 region for the HPVs17/37 miRNAs, until experimentally tested, we cannot rule out regulation of viral gene expression by these miRNAs. Since our results suggest that many PVs do not encode miRNAs, this raises the question of if/how such viruses fine-tune their own gene expression. For at least one high risk PV, HPV31, the answer seems to be by co-opting host miRNAs [25]. Gunasekharan *et al*. demonstrated that miR-145 is expressed at higher levels in undifferentiated versus differentiated keratinocytes, displaying an inverse pattern to HPV31 replication levels. miR-145 negatively regulates HPV31 genome replication and gene expression. Interestingly, miR-145 directly docks to and regulates the E1/E2 transcripts in a genomic region similar to that we have uncovered for FcPV1 and HPV41 miRNAs. Our bioinformatic analysis of all human miRNAs and fully sequenced PV genomes (S9 Dataset), similar to the published work of Gunasekharan *et al*., suggests many other PVs could utilize a similar host miRNA strategy to regulate the E1/E2 transcripts. Further, we observed a near perfect complementary putative target site for Let7 miRNA in the FcPV1 genome in the late 3' UTR, implying regulation by small interfering (siRNA)-like RISC-mediated mRNA cleavage (S4 Fig). These results further support the likelihood that diverse PVs utilize host miRNAs to regulate viral gene expression. Combined, these findings suggest that miRNAs of host and/or viral origin are utilized by PVs to optimize viral gene expression.

Our results demonstrate that miDGE can be a fruitful approach when applied to numerous viruses. However, while miDGE allowed us to make reasonable conclusions for ~63 PV genomes, some genomes that we intended to include in our miDGE procedure were under-represented in the final libraries. Although we believe one major reason for this discrepancy is due to incorrect PV genomic plasmids included in our original library pools, library coverage should be optimized in future applications of miDGE. Since miRNAs are generally stable, miDGE could be used to identify biomarkers for gene expression of unculturable pathogens. In addition to pathogen genomes that cannot be grown in culture, miDGE may have utility for identifying miRNAs expressed in only a few rare cells of an organism. For example, miR-Lys6 is only expressed in fewer than 10 cells in *Caenorhabditis elegans* and had been missed by most standard miRNA biochemical identification procedures [65]. It is likely that similar miRNAs exist in complex multicellular organisms and these could be identified using miDGE.

In summary, we have developed wet bench technology that can identify miRNAs from genomes for which complete transcriptomes are not readily available, whether viral or otherwise. This approach opens the possibilities for miRNA discovery to the enormous range of pathogens for which genomic data is available, but are unculturable in a laboratory setting. In this initial study, our approach uncovered five new PV highly probable/bona fide miRNAs. As viral miRNAs often alter host targets conducive to infection, it will be interesting to determine any relevant host targets of these miRNAs. Moreover, as our work lends additional support to the role of miRNAs in control of the PV life cycle, it will be important to determine if variability in miRNA expression or activity can contribute to the differences in tropism and pathogenesis associated with the various PV types.

## Materials and methods

### miDGE

For the construction of our JMRV miDGE library, a cosmid containing ~38kb of the viral genome (nts 83,148 to 119,569) was sonicated to produce fragments with an average size of 300-400bp. Sub-genomic fragments in the appropriate size range were purified from agarose gels, blunted and phosphorylated using the Epicentre End-It End-Repair kit and cloned into an pDNA3 vector to produce a miDGE expression library. For the PVs, plasmids containing cloned PV genomes (S2 Table) were collected from various labs. We reasoned that, due to their substantially lower size, PV genomes might shear less efficiently compared to the JMRV and therefore utilized restriction enzyme digestion in addition to the sonication protocol to produce the PV miDGE library. For this purpose, the collection of PV plasmids and the positive controls SV40 and MCPyV was divided into four sub-groups containing between 27 and 46 genomes, followed by digestion with three different 4-base pair cutter restriction enzymes (BsrFI, BstyI and EaeI). The resulting fragments were then cloned into compatible sites of the pcDNA3.1 vector (AgeI, BamHI and NotI, respectively). Coverage of individual genomes in the JMRV sonication library, or the PV fragment libraries produced by restriction-digestion or sonication was determined via high-throughput sequencing on Illumina HiSeq 2500 and MiSeq systems using the TruSeq DNA Library Prep Kit. Dataset S1 provides coverage profiles of PV genomes in bedgraph format, the percentage of individual genomes covered in our miDGE libraries is listed in S2 Table.

We transfected the JMRV and PV expression libraries into HEK293T via lipofection, harvested total RNA using PIG-B [66], and size fractionated the isolated RNA via excision from a 15% denaturing polyacrylamide gel to enrich for RNA in the size class between approximately 10–35 nucleotides. This RNA was then used to produce small-RNA libraries for Illumina sequencing. In parallel, as a positive control we generated small-RNA-seq libraries with RNA from JMRV-infected fibroblasts, kindly provided by Scott Wong (Vaccine and Gene Therapy Institute, Oregon Health & Science University, Beaverton, Oregon, USA). Since different library preparation methods can potentially result in a bias that can result in underrepresentation of individual miRNA species, we used the TruSeq Small RNA Sample Preparation Kit (Illumina) as well as the NEBNext Small RNA Library Kit (New England Biolabs) to generate Illumina-compatible sequencing libraries. The libraries were then sequenced on a HiSeq 2500 System (50 cycles, single end reads) producing a total of 30 million raw reads for JMRV-infected fibroblast, or 112 and 712 million reads for HEK293T cells transfected with JMRV or PV miDGE libraries, respectively. After trimming, reads were first mapped with bowtie v1.2.1.1 (options -n 0 -e 80 -l 18 -a -m 5—best—strata) to viral genomes (see S2 Table for PV accession numbers) to investigate viral read coverage. Non-aligned reads were subsequently aligned to the human transcriptome (ENSEMBL release-91 GRCh38 cDNA and non-coding RNA sequences) to elucidate mapping to different RNA species as shown in Table 1. Viral and host reads were then extracted from the bam alignment files, merged and collapsed using the mapper.pl script of the miRDeep2 v2.0.0.8 package [47]. The collapsed reads were then used to perform a miRDeep2 prediction of novel miRNAs, using default options and, in addition to the collapsed reads and viral genomes, providing the pipeline with the set of human precursor and mature miRNA sequences from miRBase v21 [53] as a reference. To eliminate potential low-complexity reads, prior to prediction of novel miRNAs or mapping to viral genomes, collapsed FASTA reads were optionally filtered using the prinseq-lite package v0.20.4 [67], using an entropy cutoff value of 70. Bam files containing all primary reads aligned to viral genomes or the host transcriptome are available via the European Nucleotide Archive (ENA, https://www.ebi.ac.uk/ena) under accession number PRJEB25054.

### Plasmids and cells

HEK293 or HEK293T cells were originally obtained from ATCC and maintained in DMEM supplemented with 10% (vol/vol) FBS and Pen/Strep (Cellgro). HEK293T Dicer KO cells were obtained from Dr. Bryan Cullen, Duke University (NoDice, [68]). All cells were grown at 37C in the presence of 5% CO2.

Plasmids containing the genomes of the papillomaviruses used in this study were obtained from labs indicated in S5 Dataset. A cosmid containing nts 83,148 to 119,569 of the JMRV genome (NC_007016) was kindly provided by Scott Wong (Vaccine and Gene Therapy Institute, Oregon Health & Science University, Beaverton, Oregon, USA).

The miRNA expression vectors were cloned using PCR amplification of the relevant portions (generally speaking, this contains the putative miRNA site as well as ~100–150 bp flanking said site) of the viral genome (from genomic plasmids), followed by restriction enzyme digestion and ligation. Specifically, the indicated regions of each viral genome listed in S6 Dataset were inserted into the XhoI/XbaI sites of pcDNA3.1 (Invitrogen). The SV40 miRNA expression construct is as previously described [32].

Luciferase reporter plasmids were constructed using PCR amplification of the relevant portions of the viral genome (from genomic plasmids), followed by restriction enzyme digestion and ligation. In this case, these sequences were cloned into the XhoI/XbaI sites of pcDNA3.1dsRluc which places the desired sequences into the 3’UTR of the *Renilla* Luciferase gene. Seed site mutant constructs were altered via PCR-based site directed mutagenesis at the indicated positions 2, 3, and 5 of the seed complement site (FcPV1 mutant constructs), or positions 2, 3 (HPV41 mutant construct) listed in S6 Dataset. To make the perfectly complementary RISC reporters for each miRNA, sequences with two perfectly complementary binding sites for each miRNA with a 12 nucleotide spacer region were synthesized (Integrated Data Technologies) and cloned into the Xho1/Xba1 sites of pcDNA3.1dsRLuc plasmid as listed in S6 Dataset. Respective mutants were made by mutating three nucleotides in the seed complement sequence of each binding site, also listed in S6 Dataset.

### Phylogenic analysis

Species tree was constructed using the nucleotide sequences of L1 gene region of papillomavirus genomes for all genomes with greater than 95% coverage in miDGE analysis (73 viral genomes in total). Sequences were aligned using CLUSTALW [69] in Geneious [70]. Initial tree(s) for the heuristic search were obtained automatically by applying a Neighbor-Joining algorithm [71] to a matrix of pairwise distances estimated using the Maximum Composite Likelihood (MCL) approach [72], and then selecting the topology with superior log likelihood value. The tree with the highest log likelihood is shown.

### RISC activity assay

HEK293 or HEK293T cells (as designated in figure legends) were split and plated onto twelve well dishes so that they were approximately 70% confluent the following day. These plates were then co-transfected with five ng of the indicated *Renilla* Luciferase-based reporter constructs (pcDNA3.1dsRluc [73]), five ng of Firefly Luciferase reporter (pcDNA3.1dsLuc2CP [73]) and one ug of either a control miRNA expression construct (the SV40 miRNA expression construct) or the indicated miRNA expression vector using Lipofectamine 2000 according to the manufacturer’s instructions. Transfections were carried out in triplicate for each transfection, which were considered technical replicates. Forty-eight hours later, cells were harvested with 100 uL of 1X Passive Lysis buffer from the Dual-Glo Luciferase Assay System (Promega). 5 uL of lysate from each well was then analyzed in duplicate for *Renilla* and Firefly luciferase activity with a Luminoskan Ascent luminometer (Thermo Electronic). These experiments were then performed for at least 3 biological replicates (new transfections on separate days), with the exact number noted in the individual figure legend. Data was analyzed by dividing the *Renilla* luciferase activity value by the Firefly luciferase activity value to obtain a *Renilla*/Firefly luciferase activity ratio. These ratios were then averaged between the two measures for each well of the twelve well dish. Then the averaged *Renilla*/Firefly ratio was averaged for each of the technical triplicate wells, with the resulting average used to normalize the final values such that the no miRNA control *Renilla*/Firefly ratio was set to 1. The one-sided Student’s t-test was used to assess the statistical significance of observed differences, with a P value of < 0.05 considered statistically significant. Primary data and analysis are in S7 Dataset.

### miRNA detection assay

HEK293T cells were plated into 12-well dishes so that they were approximately 70% confluent the next day. At that point, they were transfected with 1ug of the indicated miRNA expression constructs per well of cells with Lipofectamine 2000 (Invitrogen) in accordance with the manufacturer’s instructions. 48 hours post-transfection, total RNA was harvested with PIG-B and Northern blot analysis was performed as described previously [66]. Briefly, following harvest, total RNA was quantitated by NanoDrop. Equal amounts of RNA were then run on a denaturing PAGE gel and transferred to Amersham Hybond–N+ membrane (GE Healthcare). This membrane was then probed with indicated DNA oligonucleotide probe that was radioactively labeled with P32 and visualized through exposure to a phosphor screen and images were captured on the Typhoon (probe sequences indicated S6 Dataset, uncropped scans in S8 Dataset). The blots were stripped of the DNA probe through incubation with boiling water and SDS, and reprobed with different DNA oligonucleotide probes.

### Drosha dependence assay

HEK293T cells were plated into 6-well dishes so that they were approximately 70% confluent the next day. At that point, they were transfected with 20 nM Drosha siRNA (Sigma Aldrich) or negative control siRNA (Sigma Aldrich Mission siRNA SIC001) per well of cells using Lipofectamine RNAiMAX (Invitrogen) according to the manufacturer’s instructions. Forty-eight hours later these cells were trypsinized and, replated to new wells of a 6 well dish. The following day, these cells were approximately 70% confluent, and were transfected with the respective siRNAs and the indicated miRNA expression constructs or HSUR4 transfection/load control expression vector [21,74] (2ug/well as in the miRNA Detection assay) with Lipofectamine 2000 (Invitrogen) according to the manufacturer’s instructions. Forty-eight hours later, total RNA was harvested with PIG-B and Northern blot analysis was performed as previously described [66] and in miRNA Detection Assay. The membrane for Northern blot analysis was probed with DNA oligonucleotides as indicated in S6 Dataset. Signal was quantitated using Image Studio Lite software, and ratios of mature miRNA signal in negative control cells to Drosha-knockdown cells (relative to transfection/load control HSUR4 signal) were calculated for each PV miRNA (S7 and S8 Datasets).

### Dicer dependence assay

HEK293T and HEK293T Dicer KO cells (NoDice, [75]) were plated into 6 well dishes so that they were approximately 70% confluent the next day. At that point, they were transfected with 2ug/well of the indicated miRNA expression vectors using Lipofectamine 2000 (Invitrogen) in accordance with the manufacturer’s instructions. Forty-eight hours post-transfection, total RNA was harvested with PIG-B and Northern blot analysis was performed as in the miRNA Detection Assay section. The DNA oligonucleotide probes are listed in S6 Dataset. Signal was quantitated using Image Studio Lite software, and ratios of mature miRNA signal in negative control cells to Dicer KO cells (relative to the same ratio in HEK293T cells) were calculated for each PV miRNA (S7 and S8 Datasets).

### Detection of PV miRNA from *In Vivo* chaffinch leg lesions

Total RNA was extracted from both leg lesions and pectoral muscle (control) tissue from two chaffinches with proliferative leg skin lesions that were PCR-positive for FcPV1 DNA. The affected chaffinches were found dead by members of the public and submitted for post-mortem examination to a national scanning surveillance scheme for wild bird disease in Great Britain (Lawson et al. *in prep*.). Samples of leg lesions and apparently normal pectoral muscle were collected at post-mortem examination and archived at -80 ^o^C and -20 ^o^C respectively. Samples were finely minced using sterile scalpel blades, and then ~20 mg of tissue was mixed with 350 μl of RTL:β-ME solution (1 ml buffer RTL [Qiagen] with 10 μl β-mercaptoethanol) and homogenized. The lysate was centrifuged at maximum speed in a microcentrifuge for 3 min and transferred to a fresh microcentrifuge tube. Ethanol (100%) was added to the cleared lysate to bring the final concentration up to 60% ethanol. Next, the samples were applied to an RNeasy (Qiagen) mini-spin column to purify the total RNA according to the manufacturer's instructions, except that after the final wash step, the samples were stored at approximately 4° C for several days while still on the column. The final elution steps were conducted with one volume of nuclease-free water and then repeated with one volume of nuclease-free Tris-EDTA (TE), pH 7. Pooled small RNA-seq libraries were prepared from RNA harvested from either foot lesions or pectoral muscle from animals CF180/09 and CF229/12. Equal volumes of total RNA were combined and ethanol precipitated. Recovered RNA was dissolved in nuclease free water (Ambion) and quantitated with a NanoDrop spectrophotometer (ThermoFisher). Libraries were prepared using 180 ng of pooled RNA with the NEBNext Multiplex Small RNA Library Prep Set for Illumina (E7300, New England Biolabs) according to the manufacturer’s instructions with the addition of an additional round of indexing PCR to compensate for low input RNA. Libraries were quantitated with the Qubit dsDNA BR assay (ThermoFisher), QC checked with the Bioanalyzer High Sensitivity assay (Agilent Technologies), combined with other barcoded libraries, and sequenced on a single lane of a SR50 run on a HiSeq 4000 (Illumina) by the Genomic Sequencing and Analysis Facility at UT Austin. 106,251,321 and 122,129,107 reads were obtained for the foot and pectoral samples respectively (SRA accession: SRP133175). Small RNA reads were pre-processed by trimming the adaptor sequences and removing trimmed sequences shorter than 18 nucleotides with Cutadapt (version 1.4.2) [76]. Reads were mapped with SHRiMP2 (version 2.2.3) [77] to the reference sequences consisting of miRBase release 21 annotated zebra finch miRNAs [53] and FcPV1 reference sequence (NC_004068).

### RNA-seq analysis of high risk HPVs in cervical cancer

The Cancer Genome Atlas cervical cancer RNA-seq datasets were retrieved from the NCI Genomic Data Commons. From the large RNA-seq data sets, sequenced coverage was calculated for the HPV reference sequence with the greatest number of alignments. Tumors with > = 50% coverage for an HPV were used for subsequent analysis (213 small RNA data sets). Aligned BAM files were converted to miRDeep2 format and the miRDeep2 pipeline was run with default parameters without miRNA annotations. BEDTools was used to assign the de novo miRDeep2 identified miRNAs to miRBase release 21 annotations.

### Re-analysis of small RNA-seq data from Qian *et al*. [40]

Small RNA data sets associated with NCBI GEO project GSE42380 were retrieved from the NCBI SRA. The SRA files were converted to colorspace FASTQ format using the SRA Toolkit. Adapter sequences were trimmed from reads using Cutadapt (version 1.4.2) [76]. The trimmed libraries were mapped to miRBase release 21 annotated human miRNA sequences [53] and viral reference sequences using SHRiMP2 (version 2.2.3) [77] (reference sequences are listed in S7 Dataset). Resulting SAM files were converted to miRDeep2 format and the miRDeep2 pipeline was run with default parameters without miRNA annotations [47].

## Supporting information

S1 Fig Small RNA-seq read coverage across JMRV miRNAs. Each panel depicts small RNA-seq coverage in material from JMRV-infected fibroblasts (upper plots) or 293T cells transfected with our miDGE fragment library (lower plot in each panel). Gray bars under the plots indicate the location of pre-miRNA hairpins. Thicker sections denote the location of previously annotated mature miRNA sequences. Coverage is shown in a strand-specific manner for each miRNA and precursor (*i*.*e*., only reads matching the proper miRNA sequences are shown).
(TIF)

S2 Fig Small RNA-seq read coverage across PV miRNAs. Plots show small RNA-seq read coverage from HEK293T cells transfected with PV miDGE libraries for the 5 novel papillomavirus miRNAs identified in this study **(A)** and the two positive control polyomaviruses miRNAs (SV40, MCPyV) contained in our library **(B)**. Grey bars underneath the plots indicate the location of pre-miRNA hairpin sequences, with thicker boxes denoting the location of mature miRNA products. Coverage is shown in a strand-specific manner for each miRNA and precursor (*i*.*e*., only reads matching the proper miRNA sequences are shown).
(TIF)

S3 Fig Small RNA-seq read coverage across previously purported PV miRNA candidates. Plots show small RNA-seq read coverage from HEK293T cells transfected with PV miDGE libraries for 8 previously purported miRNAs in HPV types 6, 16, 38, and 45 **(A)** or 18 **(B)**. Panel **C** shows coverage of the suggest HPV type 18 precursor by reads from our JMRV miDGE library transfection, demonstrating that coverage of these sequences is nonspecific. Open bars and boxes underneath the plots in (A) indicate the location of suggested precursor and mature miRNAs, respectively. Coverage in (A) is shown in a strand-specific manner for each miRNA and precursor (*i*.*e*., only reads matching the proposed miRNA sequence are shown). For the HPV type 18 candidate shown in (B) and (C), mature miRNAs (indicated by thick block arrows) were proposed to derive from both strands of the precursor (thinner block arrow). Therefore, plots in (B) and (C) show coverage across both strands of the viral genome.
(TIF)

S4 Fig Diagram of putative let-7 microRNA target site. A diagram of the FcPV1 genomic organization is provided at the top showing the positions of the known open reading frames (ORFs). Below is a detailed view of the 3' untranslated region following the L1 ORF and the predicted base pairing with the let-7a-5p microRNA. Vertical bars "|" indicate predicted base pairing and ":" indicates predicted wobble pairing. The let-7a sequence is from the closest relative with available genomic sequence, the zebra finch.
(TIF)

S1 Table Counts of small RNA-seq reads mapped to JMRV miRNAs. The table shows counts of small RNA reads from JMRV-infected fibroblasts (“infection”) or HEK293T cells transfected with our miDGE library (“miDGE”) mapped to the indicated precursor or mature JMRV miRNA regions.
(DOCX)

S2 Table PV accession numbers and DNA-seq coverage in miDGE libraries. Column coverage provides the percentage of the viral genome that was covered by our miDGE libraries, according to high-throughput sequencing of the expression library. The column labeled acronym denotes our internal label for individual genomes (also used as the identifier in all bam files provided via the ENA archive).
(DOCX)

S3 Table PV miRNA genomic seed matches. Reference PV genomic sequences were downloaded from PAVE [78]. Each sequence was searched for the perfect complement of the indicated microRNA seed sequence (starting at position 2 of the microRNA and extending for the length of a perfect match). Matches of length 7 or greater are reported. Positions of the E1 and E2 ORFs are taken from the PAVE reference annotations.
(DOCX)

S1 Dataset DNA-seq coverage across PV genomes. This dataset contains DNA-seq coverage data across viral genomes contained in our miDGE PV libraries in bedgraph format.
(BG) Click here for additional data file.

S2 Dataset Summary of miRDeep2 predictions made for miDGE libraries. This dataset shows an overview of all predictions made by miRDeep2 for our JMRV and PV libraries. Columns C to N provide miRDeep2 prediction data in the pipeline’s standard tabular output format. Columns A and B indicate the miDGE library for which the predictions were made **(A)** and the designation of known or experimentally confirmed novel pre-miRNAs matching the miRDeep2 predictions **(B)**. ‘l.c.’ in column B indicates likely false positive predictions which can be eliminated by filtering reads for low complexity filters prior to performing the miRDeep2 analysis.
(XLSX)

S3 Dataset miRDeep2 predictions made for JMRV. This dataset shows miRDeep2’s primary output (in PDF format) of read coverage along hairpin structures for all predictions made by the pipeline for our JMRV miDGE data. Provisional IDs for the individual pre-miRNAs are given as assigned by the pipeline, see S2 Dataset for matching these IDs to known or novel pre-miRNAs.
(PDF)

S4 Dataset miRDeep2 predictions made for PVs. This dataset shows miRDeep2’s primary output (in PDF format) of read coverage along hairpin structures for all predictions made by the pipeline for our PV miDGE data (including the two PyV positive controls). Provisional IDs for the individual pre-miRNAs are given as assigned by the pipeline, see S2 Dataset for matching these IDs to known or novel pre-miRNAs.
(PDF)

S5 Dataset PV genome origin list. This dataset contains information on where each PV genomic plasmid used in this study was obtained.
(XLSX)

S6 Dataset Reporter sequences and probes. This dataset contains information about the sequences of reporters and probes used in this study.
(XLSX)

S7 Dataset Primary data associated with Figs 4–6. This dataset contains primary data used in re-analysis of Qian et al data (Fig 4B–4G), quantification of northern-blot-based experiments (Fig 5B and 5C), and luciferase-based assays (Figs 6 and 8).
(XLSX)

S8 Dataset Uncropped images of northern blots. This dataset includes the full uncropped scans of northern blots used in Fig 5.
(PDF)

S9 Dataset Positions of miRBase annotated microRNA seeds in papillomavirus genomes. This dataset includes the coordinates of human miRBase release 21 microRNA seed matches (positions 2–8 inclusive) in all PAVE annotated papillomavirus genomes.
(XLSX)
