# Supplementary figures and images for: Identification of virus-encoded microRNAs in divergent Papillomaviruses
Source: PLoS Pathog. 2018 Jul 26;14(7):e1007156. doi: 10.1371/journal.ppat.1007156 (PMC6062147; doi:10.1371/journal.ppat.1007156)

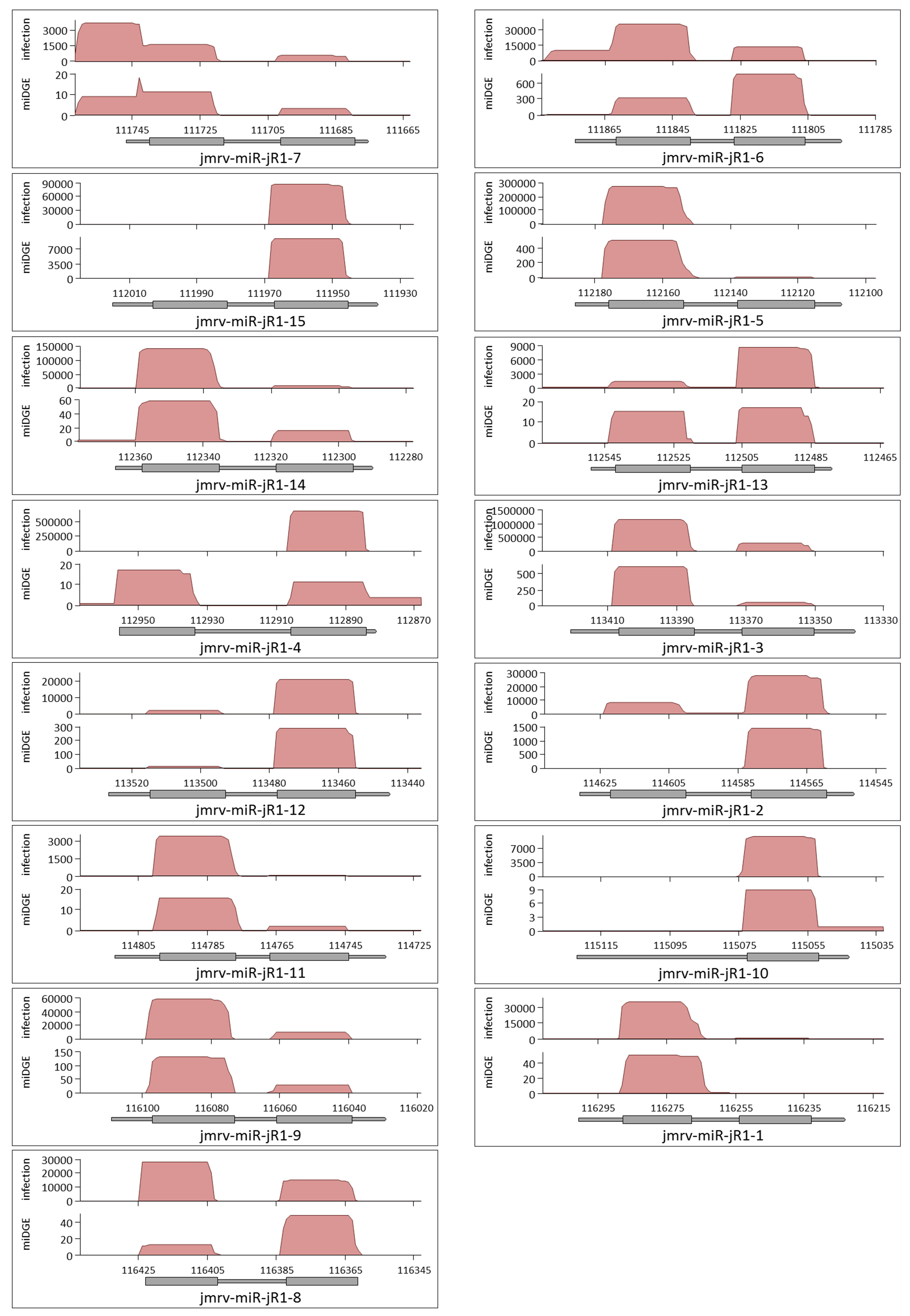

Supplement: S1 Fig — Each panel depicts small RNA-seq coverage in material from JMRV-infected fibroblasts (upper plots) or 293T cells transfected with our miDGE fragment library (lower plot in each panel). Gray bars under the plots indicate the location of pre-miRNA hairpins. Thicker sections denote the location of previously annotated mature miRNA sequences. Coverage is shown in a strand-specific manner for each miRNA and precursor (i.e., only reads matching the proper miRNA sequences are shown). (TIF) [file ppat.1007156.s001.TIF]

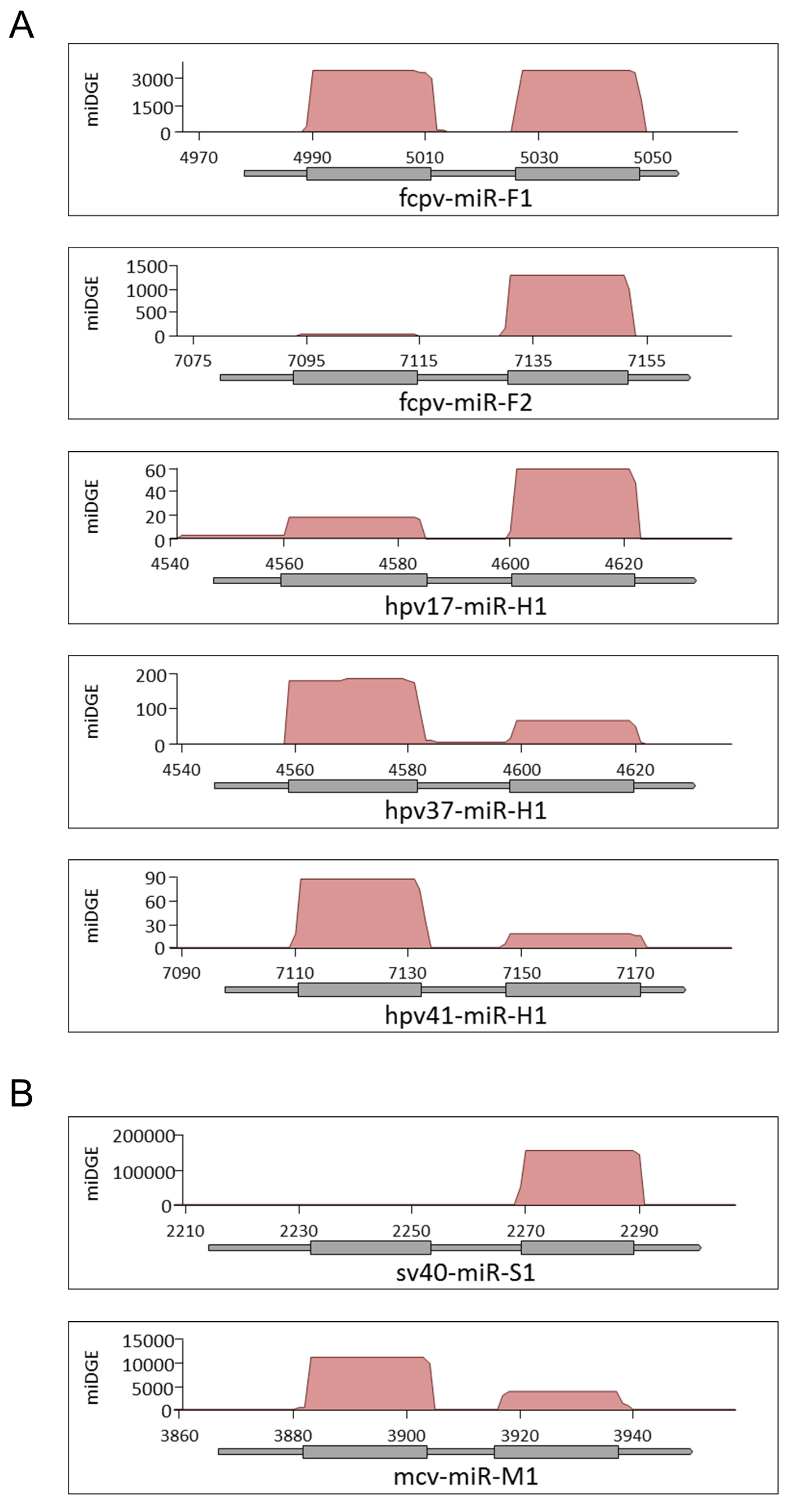

Supplement: S2 Fig — Plots show small RNA-seq read coverage from HEK293T cells transfected with PV miDGE libraries for the 5 novel papillomavirus miRNAs identified in this study (A) and the two positive control polyomaviruses miRNAs (SV40, MCPyV) contained in our library (B). Grey bars underneath the plots indicate the location of pre-miRNA hairpin sequences, with thicker boxes denoting the location of mature miRNA products. Coverage is shown in a strand-specific manner for each miRNA and precursor (i.e., only reads matching the proper miRNA sequences are shown). (TIF) [file ppat.1007156.s002.TIF]

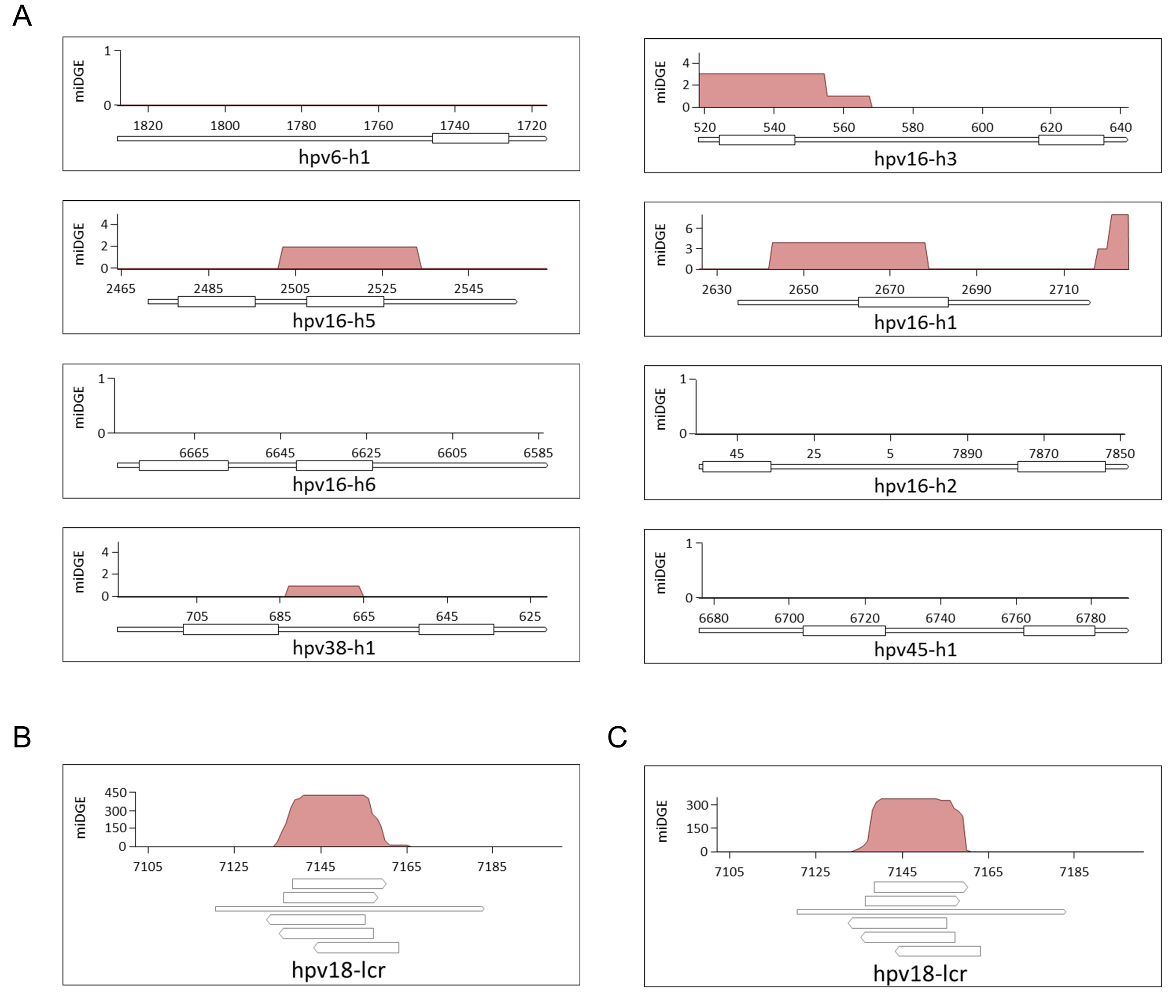

Supplement: S3 Fig — Plots show small RNA-seq read coverage from HEK293T cells transfected with PV miDGE libraries for 8 previously purported miRNAs in HPV types 6, 16, 38, and 45 (A) or 18 (B). Panel C shows coverage of the suggest HPV type 18 precursor by reads from our JMRV miDGE library transfection, demonstrating that coverage of these sequences is nonspecific. Open bars and boxes underneath the plots in (A) indicate the location of suggested precursor and mature miRNAs, respectively. Coverage in (A) is shown in a strand-specific manner for each miRNA and precursor (i.e., only reads matching the proposed miRNA sequence are shown). For the HPV type 18 candidate shown in (B) and (C), mature miRNAs (indicated by thick block arrows) were proposed to derive from both strands of the precursor (thinner block arrow). Therefore, plots in (B) and (C) show coverage across both strands of the viral genome. (TIF) [file ppat.1007156.s003.TIF]

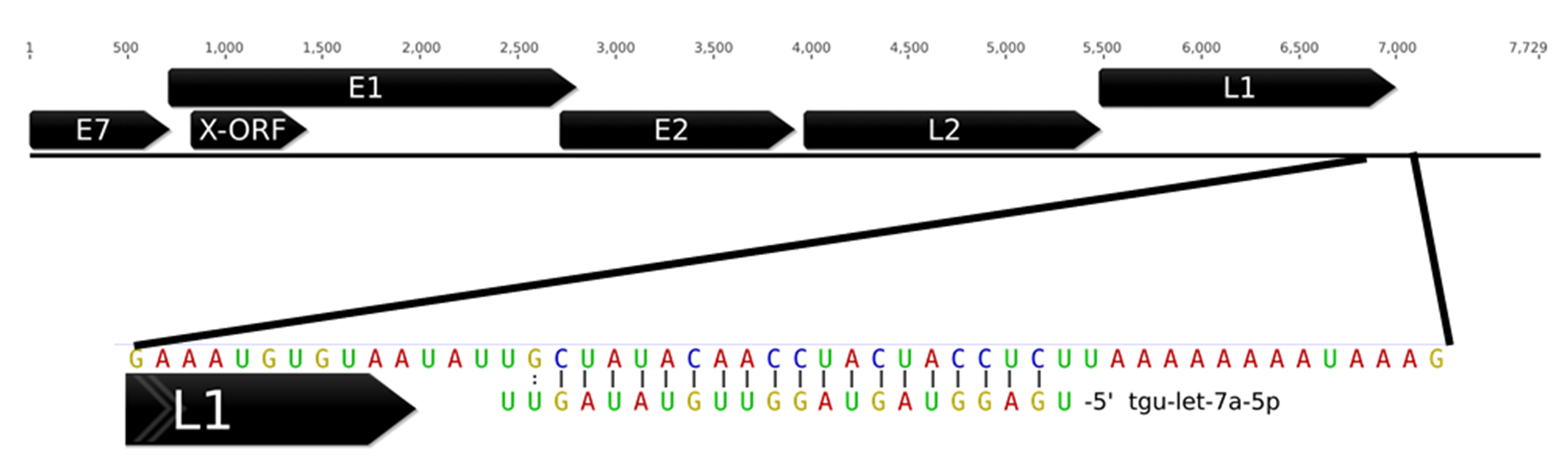

Supplement: S4 Fig — A diagram of the FcPV1 genomic organization is provided at the top showing the positions of the known open reading frames (ORFs). Below is a detailed view of the 3' untranslated region following the L1 ORF and the predicted base pairing with the let-7a-5p microRNA. Vertical bars "|" indicate predicted base pairing and ":" indicates predicted wobble pairing. The let-7a sequence is from the closest relative with available genomic sequence, the zebra finch. (TIF) [file ppat.1007156.s004.TIF]
