# Supplementary material for: Identification of virus-encoded microRNAs in divergent Papillomaviruses
Source: PLoS Pathog. 2018 Jul 26;14(7):e1007156. doi: 10.1371/journal.ppat.1007156 (PMC6062147; doi:10.1371/journal.ppat.1007156)
Supplement: S1 Table — The table shows counts of small RNA reads from JMRV-infected fibroblasts (“infection”) or HEK293T cells transfected with our miDGE library (“miDGE”) mapped to the indicated precursor or mature JMRV miRNA regions. (DOCX) [file ppat.1007156.s005.docx]

**Table S1:** **Counts of small RNA-seq reads mapped to JMRV miRNAs**

|  |  | **genomic** |  | **infection**^b^ | |  | **miDGE**^c^ | |
| --- | --- | --- | --- | --- | --- | --- | --- | --- |
|  |  | **coordinates**^a^ |  | **precursor** | **mature** |  | **precursor** | **mature** |
| jmrv-miR-jR1-7 |  | 111678..111749 (-) |  | 2,192 | 2,175 |  | 14 | 14 |
| jmrv-miR-jR1-6 |  | 111796..111875 (-) |  | 48,410 | 48,380 |  | 1,125 | 1,124 |
| jmrv-miR-jR1-15 |  | 111938..112016 (-) |  | 86,890 | 86,778 |  | 9,764 | 9,764 |
| jmrv-miR-jR1-5 |  | 112108..112187 (-) |  | 274,518 | 274,359 |  | 528 | 522 |
| jmrv-miR-jR1-14 |  | 112291..112367 (-) |  | 149,799 | 149,776 |  | 76 | 76 |
| jmrv-miR-jR1-13 |  | 112480..112550 (-) |  | 10,454 | 10,389 |  | 32 | 32 |
| jmrv-miR-jR1-4 |  | 112882..112956 (-) |  | 683,090 | 682,974 |  | 28 | 28 |
| jmrv-miR-jR1-3 |  | 113340..113422 (-) |  | 1,481,479 | 1,481,265 |  | 663 | 663 |
| jmrv-miR-jR1-12 |  | 113446..113527 (-) |  | 23,289 | 22,923 |  | 302 | 301 |
| jmrv-miR-jR1-2 |  | 114553..114632 (-) |  | 36,549 | 36,012 |  | 1,460 | 1,449 |
| jmrv-miR-jR1-11 |  | 114734..114813 (-) |  | 3,506 | 3,502 |  | 18 | 18 |
| jmrv-miR-jR1-10 |  | 115044..115123 (-) |  | 9,615 | 9,605 |  | 9 | 9 |
| jmrv-miR-jR1-9 |  | 116030..116110 (-) |  | 67,871 | 67,867 |  | 160 | 160 |
| jmrv-miR-jR1-1 |  | 116224..116301 (-) |  | 36,932 | 36,214 |  | 51 | 40 |
| jmrv-miR-jR1-8 |  | 116363..116424 (-) |  | 43,082 | 43,071 |  | 60 | 60 |

a: genomic coordinates of known JMRV miRNAs

b: reads mapped to viral precursor or mature miRNA regions in JMRV-infected fibroblasts

c: reads mapped to viral precursor or mature miRNA regions in 293T cells transfected with the JMRV miDGE expression library
