# Supplementary material for: Identification of virus-encoded microRNAs in divergent Papillomaviruses
Source: PLoS Pathog. 2018 Jul 26;14(7):e1007156. doi: 10.1371/journal.ppat.1007156 (PMC6062147; doi:10.1371/journal.ppat.1007156)
Supplement: S2 Table — Column coverage provides the percentage of the viral genome that was covered by our miDGE libraries, according to high-throughput sequencing of the expression library. The column labeled acronym denotes our internal label for individual genomes (also used as the identifier in all bam files provided via the ENA archive). (DOCX) [file ppat.1007156.s006.docx]

**Table S2:** **Accession numbers and DNA-seq coverage of Papillomavirus genomes investigated by miDGE**

| **accession** | **title** | **latin name** | **acronym** | **cove-rage** |
| --- | --- | --- | --- | --- |
| NC_015267.1 | Camelus dromedarius papillomavirus type 1, complete genome | Camelus dromedarius papillomavirus type 1 | CdPV1 | 100.0% |
| NC_015268.1 | Camelus dromedarius papillomavirus type 2, complete genome | Camelus dromedarius papillomavirus type 2 | CdPV2 | 100.0% |
| GU117620.1 | Delphinus delphis papillomavirus, complete genome | Delphinus delphis papillomavirus | DdPV | 97.0% |
| FJ379293.1 | European hedgehog papillomavirus, complete genome | Erinaceus europaeus papillomavirus 1 | EHPV1 | 100.0% |
| NC_004068.1 | Fringilla coelebs papillomavirus, complete genome | Etapapillomavirus 1 | FcPV1 | 100.0% |
| NC_004765.1 | Felis domesticus papillomavirus type 1, complete genome | Felis domesticus papillomavirus type 1 | FdPV1 | 100.0% |
| NC_001356.1 | Human papillomavirus - 1, complete genome | Mupapillomavirus 1 | HPV1 | 100.0% |
| NC_001352.1 | Human papillomavirus - 2, complete genome | Alphapapillomavirus 4 | HPV2 | 100.0% |
| X74462.1 | Human papillomavirus type 3 genomic DNA | Human papillomavirus type 3 | HPV3 | 100.0% |
| NC_001457.1 | Human papillomavirus type 4, complete genome | Human papillomavirus type 4 | HPV4 | 100.0% |
| NC_001531.1 | Human papillomavirus - 5, complete genome | Human papillomavirus type 5 | HPV5 | 100.0% |
| NC_001355.1 | Human papillomavirus type 6b, complete genome | Human papillomavirus type 6b | HPV6b | 100.0% |
| NC_001595.1 | Human papillomavirus type 7, complete genome | Human papillomavirus type 7 | HPV7 | 90.4% |
| M12737.1 | Human papillomavirus type 8, complete genome | Human papillomavirus type 8 | HPV8 | 92.5% |
| NC_001596.1 | Human papillomavirus type 9, complete genome | Human papillomavirus type 9 | HPV9 | 100.0% |
| NC_001576.1 | Human papillomavirus type 10, complete genome | Human papillomavirus type 10 | HPV10 | 18.4% |
| M14119.1 | Human papillomavirus type 11 (HPV-11) complete genome | Human papillomavirus type 11 | HPV11 | 100.0% |
| X74466.1 | Human papillomavirus type 12 genomic DNA | Human papillomavirus type 12 | HPV12 | 100.0% |
| X62843.1 | Human papilloma virus type 13 DNA | Human papillomavirus type 13 | HPV13 | 100.0% |
| X74468.1 | Human papillomavirus type 15 genomic DNA | Human papillomavirus type 15 | HPV15 | 3.7% |
| NC_001526.2 | Human papillomavirus type 16, complete genome | Human papillomavirus type 16 | HPV16 | 100.0% |
| X74469.1 | Human papillomavirus type 17 genomic DNA | Human papillomavirus type 17 | HPV17 | 100.0% |
| NC_001357.1 | Human papillomavirus - 18, complete genome | Alphapapillomavirus 7 | HPV18 | 100.0% |
| X74470.1 | Human papillomavirus type 19 genomic DNA | Human papillomavirus type 19 | HPV19 | 100.0% |
| U31778.1 | Human papillomavirus type 20, complete genome | Human papillomavirus type 20 | HPV20 | 99.9% |
| U31779.1 | Human papillomavirus type 21, complete genome | Human papillomavirus type 21 | HPV21 | 100.0% |
| U31780.1 | Human papillomavirus type 22, complete genome | Human papillomavirus type 22 | HPV22 | 100.0% |
| U31781.1 | Human papillomavirus type 23, complete genome | Human papillomavirus type 23 | HPV23 | 100.0% |
| U31782.1 | Human papillomavirus type 24, complete genome | Human papillomavirus type 24 | HPV24 | 100.0% |
| NC_001583.1 | Human papillomavirus type 26, complete genome | Human papillomavirus type 26 | HPV26 | 99.7% |
| X74473.1 | Human papillomavirus type 27 genomic DNA | Human papillomavirus type 27 | HPV27 | 47.4% |
| U31783.1 | Human papillomavirus type 28, complete genome | Human papillomavirus type 28 | HPV28 | 100.0% |
| U31784.1 | Human papillomavirus type 29, complete genome | Human papillomavirus type 29 | HPV29 | 2.9% |
| X74474.1 | Human papillomavirus type 30 genomic DNA | Human papillomavirus type 30 | HPV30 | 88.9% |
| J04353.1 | Human papillomavirus type 31 (HPV-31) complete genome | Human papillomavirus type 31 | HPV31 | 31.7% |
| NC_001586.1 | Human papillomavirus type 32, complete genome | Human papillomavirus type 32 | HPV32 | 66.1% |
| M12732.1 | Human papillomavirus type 33, complete genome | Human papillomavirus type 33 | HPV33 | 65.8% |
| NC_001587.1 | Human papillomavirus type 34, complete genome | Human papillomavirus type 34 | HPV34 | 99.7% |
| U31785.1 | Human papillomavirus type 36, complete genome | Human papillomavirus type 36 | HPV36 | 100.0% |
| U31786.1 | Human papillomavirus type 37, complete genome | Human papillomavirus type 37 | HPV37 | 100.0% |
| U31787.1 | Human papillomavirus type 38, complete genome | Human papillomavirus type 38 | HPV38 | 100.0% |
| M62849.1 | Human papillomavirus ORFs | Human papillomavirus type 39 | HPV39 | 94.5% |
| X74478.1 | Human papillomavirus type 40 genomic DNA | Human papillomavirus type 40 | HPV40 | 93.2% |
| NC_001354.1 | Human papillomavirus type 41, complete genome | Human papillomavirus type 41 | HPV41 | 99.6% |
| GQ472847.1 | Human papillomavirus type 42 isolate TJ43-42, complete genome | Human papillomavirus type 42 | HPV42 | 100.0% |
| X74479.1 | Human papillomavirus type 45 genomic DNA | Human papillomavirus type 45 | HPV45 | 100.0% |
| M32305.1 | Human papillomavirus type 47 (HPV-47) +-sense strand | Human papillomavirus type 47 | HPV47 | 99.6% |
| NC_001690.1 | Human papillomavirus type 48, complete genome | Human papillomavirus type 48 | HPV48 | 100.0% |
| NC_001591.1 | Human papillomavirus type 49, complete genome | Human papillomavirus type 49 | HPV49 | 100.0% |
| NC_001691.1 | Human papillomavirus type 50, complete genome | Human papillomavirus type 50 | HPV50 | 100.0% |
| M62877.1 | Human papilloma virus type 51 genomic DNA, partial sequence | Human papillomavirus type 51 | HPV51 | 97.1% |
| X74481.1 | Human papillomavirus type 52 genomic DNA | Human papillomavirus type 52 | HPV52 | 100.0% |
| NC_001593.1 | Human papillomavirus type 53, complete genome | Human papillomavirus type 53 | HPV53 | 95.3% |
| NC_001676.1 | Human papillomavirus 54, complete genome | Alphapapillomavirus 13 | HPV54 | 17.1% |
| X55965.1 | Human papillomavirus type 57 complete DNA | Human papillomavirus type 57 | HPV57 | 99.8% |
| D90400.1 | Human papillomavirus type 58 complete genome | Human papillomavirus type 58 | HPV58 | 100.0% |
| X77858.1 | Human papilloma virus type 59, complete viral genome | Human papillomavirus type 59 | HPV59 | 73.0% |
| NC_001458.1 | Human papillomavirus type 63, complete genome | Human papillomavirus type 63 | HPV63 | 100.0% |
| X70829.1 | Human papillomavirus type 65 complete genome | Human papillomavirus type 65 | HPV65 | 100.0% |
| U31794.1 | Human papillomavirus type 66, complete genome | Human papillomavirus type 66 | HPV66 | 0.9% |
| D21208.1 | Human papillomavirus type 67 complete genome | Human papillomavirus type 67 | HPV67 | 100.0% |
| DQ080079.1 | Human papillomavirus type 68a, complete genome | Human papillomavirus type 68a | HPV68a | 2.9% |
| AB027020.1 | Human papillomavirus type 69 DNA, complete genome | Human papillomavirus type 69 | HPV69 | 98.6% |
| U21941.1 | Human papillomavirus type 70, complete genome | Human papillomavirus type 70 | HPV70 | 14.5% |
| AB040456.1 | Human papillomavirus type 71 DNA, complete genome | Human papillomavirus type 71 | HPV71 | 2.1% |
| X94164.1 | Human papillomavirus type 72 E6, E7, E1A, E1B, E2, E4, L2, and L1 genes | Human papillomavirus type 72 | HPV72 | 3.5% |
| AF436130.1 | Human papillomavirus type 74 subtype AE10, complete genome | Human papillomavirus type 74 | HPV74 | 2.5% |
| Y15173.1 | Human papillomavirus type 75 E6, E7, E1, E2, E4, L2, and L1 genes | Human papillomavirus type 75 | HPV75 | 3.9% |
| Y15174.1 | Human papillomavirus type 76 E6, E7, E1, E2, E4, L2, and L1 genes | Human papillomavirus type 76 | HPV76 | 91.2% |
| AJ620209.1 | Human papillomavirus type 81 complete genome | Human papillomavirus type 81 | HPV81 | 95.7% |
| AB027021.1 | Human papillomavirus type 82 DNA, complete genome | Human papillomavirus type 82 | HPV82 | 98.4% |
| NC_004500.1 | Human papillomavirus type 92, complete genome | Human papillomavirus type 92 | HPV92 | 100.0% |
| AY382778.1 | Human papillomavirus type 93, complete genome | Human papillomavirus type 93 | HPV93 | 29.7% |
| AJ620211.1 | Human papillomavirus type 94 complete genome | Human papillomavirus type 94 | HPV94 | 12.8% |
| NC_005134.2 | Human papillomavirus type 96, complete genome | Human papillomavirus type 96 | HPV96 | 100.0% |
| FM955837.2 | Human papillomavirus type 98, complete genome | Human papillomavirus type 98 | HPV98 | 38.4% |
| FM955838.1 | Human papillomavirus type 99, complete genome | Human papillomavirus type 99 | HPV99 | 94.2% |
| FM955839.1 | Human papillomavirus type 100, complete genome | Human papillomavirus type 100 | HPV100 | 99.9% |
| FM955840.1 | Human papillomavirus type 104, complete genome | Human papillomavirus type 104 | HPV104 | 79.5% |
| FM955841.1 | Human papillomavirus type 105, complete genome | Human papillomavirus type 105 | HPV105 | 20.6% |
| EF422221.1 | Human papillomavirus type 107, complete genome | Human papillomavirus type 107 | HPV107 | 99.9% |
| NC_012213.1 | Human papillomavirus type 108, complete genome | Human papillomavirus type 108 | HPV108 | 100.0% |
| NC_012485.1 | Human papillomavirus type 109, complete genome | Human papillomavirus type 109 | HPV109 | 0.7% |
| EU410348.1 | Human papillomavirus type 110, complete genome | Human papillomavirus type 110 | HPV110 | 100.0% |
| EU410349.1 | Human papillomavirus type 111, complete genome | Human papillomavirus type 111 | HPV111 | 92.7% |
| NC_012486.1 | Human papillomavirus type 112, complete genome | Human papillomavirus type 112 | HPV112 | 100.0% |
| FM955842.1 | Human papillomavirus type 113, complete genome | Human papillomavirus type 113 | HPV113 | 99.4% |
| GQ246950.1 | Human papillomavirus type 117 clone K6, complete genome | Human papillomavirus type 117 | HPV117 | 100.0% |
| GQ246951.1 | Human papillomavirus type 118 clone K2, complete genome | Human papillomavirus type 118 | HPV118 | 97.7% |
| FN547152.2 | Human papillomavirus type 125 complete genome, strain SIBX9 | Human papillomavirus type 125 | HPV125 | 12.4% |
| HM011570.1 | Gammapapillomavirus HPV127 isolate R3a, complete genome | Human papillomavirus type 127 | HPV127 | 100.0% |
| NC_014952.1 | Human papillomavirus type 128, complete genome | Human papillomavirus type 128 | HPV128 | 100.0% |
| NC_014953.1 | Human papillomavirus type 129, complete genome | Human papillomavirus type 129 | HPV129 | 100.0% |
| GU117630.1 | Human papillomavirus type 130, complete genome | Human papillomavirus type 130 | HPV130 | 98.3% |
| NC_014954.1 | Human papillomavirus type 131, complete genome | Human papillomavirus type 131 | HPV131 | 3.6% |
| NC_014955.1 | Human papillomavirus type 132, complete genome | Human papillomavirus type 132 | HPV132 | 83.3% |
| GU117633.1 | Human papillomavirus type 133, complete genome | Human papillomavirus type 133 | HPV133 | 98.4% |
| NC_014956.1 | Human papillomavirus type 134, complete genome | Human papillomavirus type 134 | HPV134 | 97.7% |
| GU129016.1 | Human papillomavirus type 148, complete genome | Human papillomavirus type 148 | HPV148 | 0.5% |
| GU117629.1 | Human papillomavirus type 149, complete genome | Human papillomavirus type 149 | HPV149 | 98.3% |
| X74467.1 | Human papillomavirus type 14D genomic DNA | Human papillomavirus type 14D | HPV14D | 99.4% |
| FN677755.1 | Human papillomavirus type 150, complete genome, isolate SIBX1 | Human papillomavirus type 150 | HPV150 | 4.4% |
| FN677756.1 | Human papillomavirus type 151, complete genome, isolate SIBX2 | Human papillomavirus type 151 | HPV151 | 17.6% |
| AY904722.1 | Lynx rufus papillomavirus type 1, complete genome | Lynx rufus papillomavirus type 1 | LrPV1 | 100.0% |
| AF420235.1 | Psittacus erithacus timneh papillomavirus, complete genome | Thetapapillomavirus 1 | PePV | 100.0% |
| AY904724.1 | Panthera leo persica papillomavirus type 1, complete genome | Panthera leo persica papillomavirus type 1 | PlpPV1 | 100.0% |
| NC_018074.1 | Phocoena phocoena papillomavirus 1, complete genome | Phocoena phocoena papillomavirus 1 | PpPV1 | 100.0% |
| NC_001678.1 | Rhesus monkey papillomavirus, complete genome | Alphapapillomavirus 12 | RhPV1 | 30.0% |
| GQ180114.1 | Rattus norvegicus papillomavirus 1 EES-2009, complete genome | Rattus norvegicus papillomavirus 1 EES-2009 | RnPV1 | 100.0% |
| EF395819.1 | Sus scrofa papillomavirus type 1 isolate variant b, complete genome | Sus scrofa papillomavirus 1 | SsPV1 | 51.2% |
| NC_011109.1 | Tursiops truncatus papillomavirus type 1, complete genome | Tursiops truncatus papillomavirus 1 | TtPV1 | 100.0% |
| NC_010739.1 | Ursus maritimus papillomavirus 1, complete genome | Ursus maritimus papillomavirus 1 | UmPV1 | 70.4% |
| DQ180494.1 | Uncia uncia papillomavirus type 1, complete genome | Uncia uncia papillomavirus type 1 | UuPV1 | 5.7% |
| NC_010277.1 | Merkel cell polyomavirus, complete genome | Merkel cell polyomavirus | MCPyV | 68.3% |
| NC_001669.1 | Simian virus 40, complete genome | Simian virus 40 | SV40 | 35.7% |
