# Supplementary material for: Identification of virus-encoded microRNAs in divergent Papillomaviruses
Source: PLoS Pathog. 2018 Jul 26;14(7):e1007156. doi: 10.1371/journal.ppat.1007156 (PMC6062147; doi:10.1371/journal.ppat.1007156)
Supplement: S3 Table — Reference PV genomic sequences were downloaded from PAVE [78]. Each sequence was searched for the perfect complement of the indicated microRNA seed sequence (starting at position 2 of the microRNA and extending for the length of a perfect match). Matches of length 7 or greater are reported. Positions of the E1 and E2 ORFs are taken from the PAVE reference annotations. (DOCX) [file ppat.1007156.s007.docx]

**Table S3: Complementary genomic regions for PV miRNAs**

| **Virus** | **miRNA** | **Seed Length** | **Genomic Position** | **Genomic Sequence** | **E1 CDS Position** | **E2 CDS Position** |
| --- | --- | --- | --- | --- | --- | --- |
| FcPV1 | FcPV1-F1-3p | 10mer | 2552 | GGGAACCGAT | 902 - 2731 | 2673 - 4031 |
| FcPV1 | FcPV1-F2-5p | 7mer | 1714 | TGTAGAT | 902 - 2731 | 2673 - 4031 |
| FcPV1 | FcPV1-F2-3p | 7mer | 3713 | GACCATA | 902 - 2731 | 2673 - 4031 |
| HPV41 | HPV41-H1-5p | 7mer | 1775;2882;3632 | AGGACAC | 951 - 2795 | 2728 - 3891 |
| HPV41 | HPV41-H1-3p | 8mer | 5965;6093 | ACTGACCA | 951 - 2795 | 2728 - 3891 |
| HPV17 | HPV17-H1-5p | 7mer | 6000 | TCCTGAA | 902 - 2731 | 2673 - 4031 |
| HPV37 | HPV37-H1-5p | 8mer | 4549 | CTCCTGTA | 899 - 2728 | 2670 - 4034 |
