# Supplementary material for: Identification of virus-encoded microRNAs in divergent Papillomaviruses
Source: PLoS Pathog. 2018 Jul 26;14(7):e1007156. doi: 10.1371/journal.ppat.1007156 (PMC6062147; doi:10.1371/journal.ppat.1007156)
Supplement: S3 Dataset — This dataset shows miRDeep2’s primary output (in PDF format) of read coverage along hairpin structures for all predictions made by the pipeline for our JMRV miDGE data. Provisional IDs for the individual pre-miRNAs are given as assigned by the pipeline, see S2 Dataset for matching these IDs to known or novel pre-miRNAs. (PDF) [file ppat.1007156.s010.pdf]

Provisional ID	:	JMRV_212
Score total	:	4923.3
Score for star read(s)	:	3.9
Score for read counts	:	4915.9
Score for mfe	:	2
Score for randfold	:	1.6
Score for cons. seed	:	
Total read count	:	9654
Mature read count	:	9648
Loop read count	:	0
Star read count	:	6

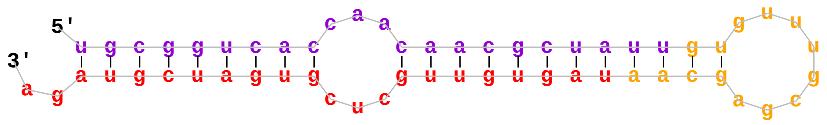

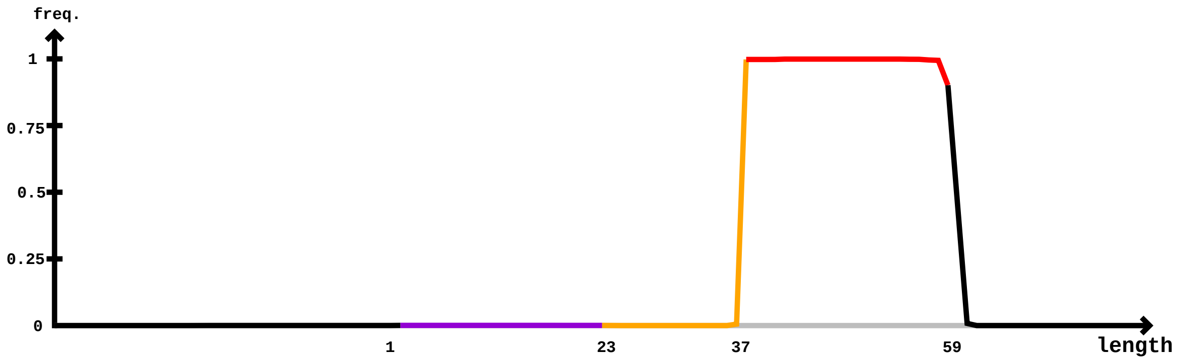

Star Mature

```
5'- ggaccaugucccugcauaucaggauuuggcaguuugcggucaccaacaacgcuauuguguuugcgagcaauaguguugcucgugaucguagaacgccagucacuuuuguacg   -3'   obs
    ggaccaugucccugcauaucaggauuuggcaguuugcggucaccaacaacgcuauuguguuugcgagcaauaguguugcucgugaucguagaacgccagucacuuuuguacg         exp
    ((((...)))).((((....((((((.(((..(((((((((((...((((((((((((........))))))))))))...)))))))))))..))))))).))..))))..   reads   mm   sample
    .............gcauaucaggauuuggcaguu..............................................................................   1       0    seq
    ..............cauaucaggauuuggcaguu..............................................................................   1       0    seq
    ..................................ugcggucaccaacaacg.............................................................   1       0    seq
    ..................................ugcggucaccaacaacgcuauu........................................................   5       0    seq
    .....................................................................auaguguugcucgugaucgua......................   2       0    seq
    .....................................................................auaguguugcucgugaucguaU.....................   1       1    seq
    .....................................................................auaguguugcucgugaucguaA.....................   1       1    seq
    .....................................................................auaguguugcucgugaucguag.....................   3       0    seq
    .....................................................................auaguguugcucgugaucguagU....................   5       1    seq
    .....................................................................auaguguugcucgugaucguaga....................   35      0    seq
    .....................................................................auaguguugcucgugaucguagC....................   1       1    seq
    .....................................................................auaguguugcucgugaucguagaa...................   3       0    seq
    .....................................................................auaguguugcucgugaucguagaaA..................   1       1    seq
    ......................................................................uaguguugcucgugauc.........................   6       0    seq
    ......................................................................uaguguugcucgugaucg........................   1       0    seq
    ......................................................................uaguguugcucgugaucgu.......................   22      0    seq
    ......................................................................uaguguugcucgugaucgC.......................   2       1    seq
    ......................................................................uaguguugcucgugaucgG.......................   1       1    seq
    ......................................................................uaguguugcucgugaucgua......................   12      0    seq
    ......................................................................uaguguugcucgugaucguaC.....................   2       1    seq
    ......................................................................uaguguugcucgugaucguag.....................   876     0    seq
    ......................................................................uaguguugcucgugaucguaA.....................   15      1    seq
    ......................................................................uaguguugcucgugaucguaU.....................   1       1    seq
    ......................................................................uaguguugcucgugaucgCag.....................   1       1    seq
    ......................................................................uaguguugcucgugaucguagU....................   511     1    seq
    ......................................................................uaguguugcucgugaucguGga....................   14      1    seq
    ......................................................................uaguguugcucgugaucguaAa....................   3       1    seq
    ......................................................................uaguguugcucgugaucguagC....................   23      1    seq
    ......................................................................uaguguugcucgugaucguaCa....................   1       1    seq
    ......................................................................uaguguugcucgugaucguCga....................   1       1    seq
    ......................................................................uaguguugcucgugaucguagG....................   68      1    seq
    ......................................................................uaguguugcucgugaucguaga....................   7325    0    seq
    ......................................................................uaguguugcucgugaucgCaga....................   9       1    seq
```

```
                                  Star                                Mature
ggaccaugucccugcauaucaggauuuggcaguuugcggucaccaacaacgcuauuguguuugcgagcaauaguguugcucgugaucguagaacgccagucacuuuuguacg
......................................................................uaguguugcucgugaucguUgaa...................   1     1     seq
......................................................................uaguguugcucgugaucguagaU...................   161   1     seq
......................................................................uaguguugcucgugaucguagaa...................   414   0     seq
......................................................................uaguguugcucgugaucguagGa...................   2     1     seq
......................................................................uaguguugcucgugaucguagUa...................   3     1     seq
......................................................................uaguguugcucgugaucguagaC...................   24    1     seq
......................................................................uaguguugcucgugaucgCagaa...................   1     1     seq
......................................................................uaguguugcucgugaucguagaG...................   6     1     seq
......................................................................uaguguugcucgugaucguagCa...................   1     1     seq
......................................................................uaguguugcucgugaucguagaaA..................   57    1     seq
......................................................................uaguguugcucgugaucguagaUc..................   2     1     seq
......................................................................uaguguugcucgugaucguagaaU..................   17    1     seq
.........................................................................uguugcucgugaucguaga....................   1     0     seq
..........................................................................guugcucgugaucguaga....................   11    0     seq
..........................................................................guugcucgugaucguagaaU..................   1     1     seq
```

Provisional ID	:	JMRV_210
Score total	:	570.9
Score for star read(s)	:	3.9
Score for read counts	:	563.5
Score for mfe	:	1.9
Score for randfold	:	1.6
Score for cons. seed	:	
Total read count	:	1117
Mature read count	:	788
Loop read count	:	0
Star read count	:	329

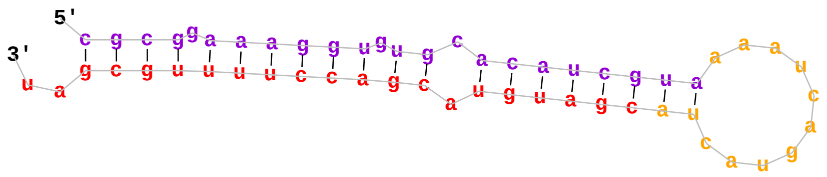

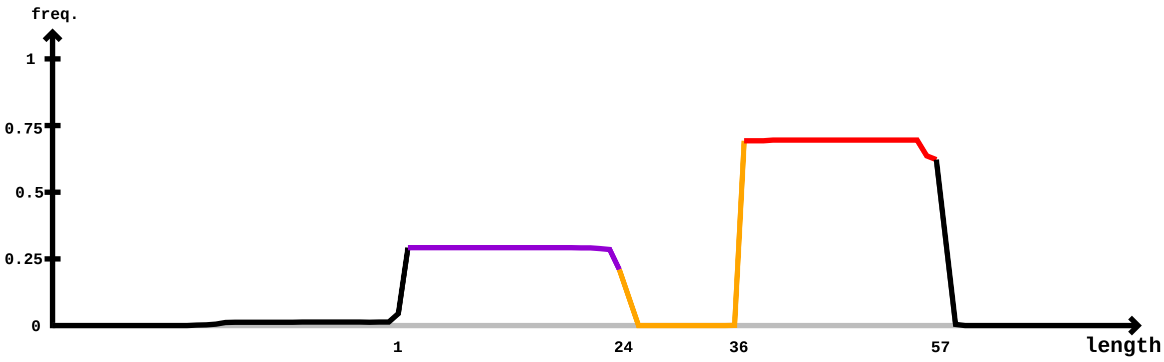

Star Mature

```
5'- gcccaggugaacugcuuuuguuguuggcggugaaccgcggaaaggugugcacaucguaaaaucaguacuacgauguacgaccuuuugcgauuuaccgccaaucuuuaacgg  -3'   obs
    gcccaggugaacugcuuuuguuguuggcggugaaccgcggaaaggugugcacaucguaaaaucaguacuacgauguacgaccuuuugcgauuuaccgccaaucuuuaacgg         exp
    ...(((.....)))....((((((((((((((((.((((.((((((.((.((((((((..........)))))))).)))))))))))).))))))))))))....)))).   reads   mm   sample
    .............gcuuuuguuguuggcggu................................................................................   1       0    seq
    .............gcuuuuguuguuggcggugaac............................................................................   1       0    seq
    ..............cuuuuguuguuggcggugaa.............................................................................   1       0    seq
    ...............uuuuguuguuggcggugaac............................................................................   3       0    seq
    ................uuuguuguuggcggugaac............................................................................   7       0    seq
    .................uuguuguuggcggugaac............................................................................   1       0    seq
    ........................uggcggugaaccgcggaaaggugugcacaucgC......................................................   1       1    seq
    ................................aaccgcggaaaggugugcacaucgA......................................................   1       1    seq
    ..................................ccgcggaaaggugugcacaucg.......................................................   2       0    seq
    ..................................ccgcggaaaggugugcacaucgC......................................................   2       1    seq
    ..................................ccgcggaaaggugugcacaucgu......................................................   19      0    seq
    ..................................ccgcggaaaggugugcacaucgua.....................................................   10      0    seq
    ..................................ccgcggaaaggugugcacaucguaC....................................................   1       1    seq
    ..................................ccgcggaaaggugugcacaucguaU....................................................   3       1    seq
    ...................................cgcggaaaggugugcaca..........................................................   1       0    seq
    ...................................cgcggaaaggugugcacauc........................................................   3       0    seq
    ...................................cgcggaaaggugugcacaucg.......................................................   2       0    seq
    ...................................cgcggaaaggugugcacaucgu......................................................   62      0    seq
    ...................................cgcggaaaggugugcacaucgua.....................................................   155     0    seq
    ...................................cgcggaaaggugugcacauAgua.....................................................   1       1    seq
    ...................................cgcggaaaggugugcacaucgCa.....................................................   2       1    seq
    ...................................cgcggaaaggugugcacaucguU.....................................................   6       1    seq
    ...................................cgcggaaaggugugcacaucguaC....................................................   2       1    seq
    ...................................cgcggaaaggugugcacaucguaa....................................................   10      0    seq
    ...................................cgcggaaaggugugcacaucguaU....................................................   48      1    seq
    .....................................................................acgauguacgaccuuuugcgauuu..................   1       0    seq
    ......................................................................cgauguacgaccuuuugcU......................   1       1    seq
    ......................................................................cgauguacgaccuuuugcC......................   1       1    seq
    ......................................................................cgauguacgaccuuuugcg......................   63      0    seq
    ......................................................................cgauguacgaccuuuugcA......................   2       1    seq
    ......................................................................cgauguacgaccuuuugcgG.....................   1       1    seq
    ......................................................................cgauguacgaccuuuugcga.....................   15      0    seq
    ......................................................................cgauguacgaccuuuugcgaC....................   15      1    seq
```

```
                                   Star                              Mature
gcccaggugaacugcuuuuguuguuggcggugaaccgcggaaaggugugcacaucguaaaaucaguacuacgauguacgaccuuuugcgauuuaccgccaaucuuuaacgg
......................................................................cgauguacgaccuuuugcgCu....................   1     1    seq
......................................................................cgauguacgaccuuuugcgaA....................   2     1    seq
......................................................................cgauguacgaccuuuugcgau....................   475   0    seq
......................................................................cgauguacgaccuuuugcgaG....................   1     1    seq
......................................................................cgauguacgaccuuuugcgaCu...................   2     1    seq
......................................................................cgauguacgaccuuuugcgCuu...................   1     1    seq
......................................................................cgauguacgaccuuuugcgauu...................   147   0    seq
......................................................................cgauguacgaccuuuugcgaGu...................   1     1    seq
......................................................................cgauguacgaccuuuugcgauC...................   6     1    seq
......................................................................cgauguacgaccuuuugcgauA...................   40    1    seq
......................................................................cgauguacgaccuuuugcgauG...................   6     1    seq
......................................................................cgauguacgaccuuuugcgauuu..................   3     0    seq
......................................................................cgauguacgaccuuuugcgauuA..................   1     1    seq
.........................................................................uguacgaccuuuugcgau....................   3     0    seq
```

Provisional ID	:	JMRV_221
Score total	:	337.8
Score for star read(s)	:	3.9
Score for read counts	:	330.5
Score for mfe	:	1.8
Score for randfold	:	1.6
Score for cons. seed	:	
Total read count	:	660
Mature read count	:	612
Loop read count	:	0
Star read count	:	48

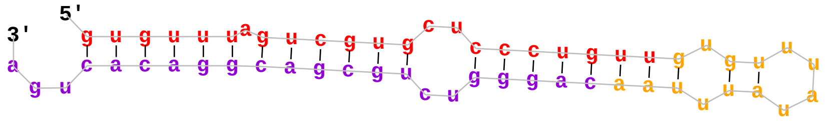

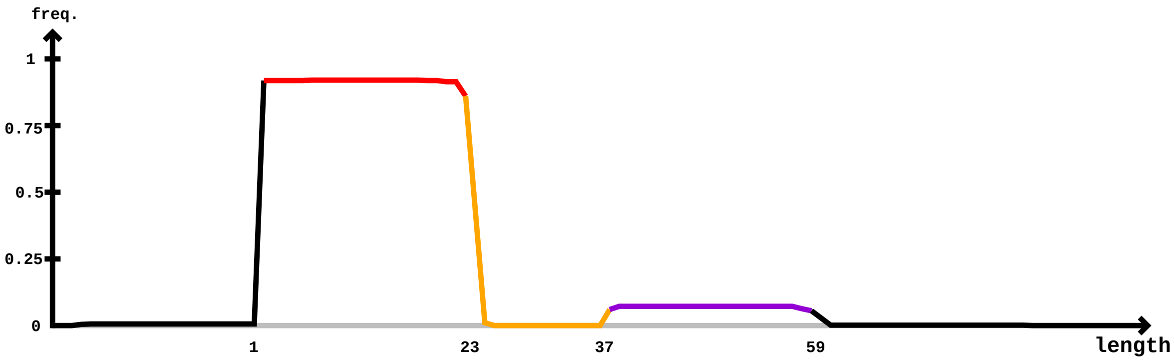

Mature   Star

```
5'- gcucaaugagcaguuagucuguguuuagucgugcucccuguuguguuuauauuuaacagggucugcgacggacacugauucgcugcuuacagucgccguuuaacauuugcua   -3'   obs
    gcucaaugagcaguuagucuguguuuagucgugcucccuguuguguuuauauuuaacagggucugcgacggacacugauucgcugcuuacagucgccguuuaacauuugcua         exp
    ((.(..((((((((.((((.((((((.((((((..((((((((.((....)).))))))))..))))))))))))).)))).))))))))..)..))................   reads   mm   sample
    .cucaaugagcaguuagucu............................................................................................   3       0    seq
    ..ucaaugagcaguuagucu............................................................................................   1       0    seq
    ....................guguuuagucgugcucc...........................................................................   1       0    seq
    ....................guguuuagucgugcucccu.........................................................................   3       0    seq
    ....................guguuuagucgugcucccCgu.......................................................................   1       1    seq
    ....................guguuuagucgugcucccugC.......................................................................   4       1    seq
    ....................guguuuagucgugcucccugu.......................................................................   29      0    seq
    ....................guguuuagucgugcucccugA.......................................................................   1       1    seq
    ....................guguuuagucgugcucccugG.......................................................................   1       1    seq
    ....................guguuuagucgugcucccuguG......................................................................   1       1    seq
    ....................guguuuagucgugcucccuguC......................................................................   13      1    seq
    ....................guguuuagucgugcucccuguu......................................................................   480     0    seq
    ....................guguuuagucgugcucccCguu......................................................................   2       1    seq
    ....................guguuuagucgugcucccugCu......................................................................   4       1    seq
    ....................guguuuagucgugcucccuguA......................................................................   5       1    seq
    ....................guguuuagucgugcucccuguuU.....................................................................   39      1    seq
    ....................guguuuagucgugcucccuguuC.....................................................................   1       1    seq
    ....................guguuuagucgugcucccuguug.....................................................................   2       0    seq
    ....................guguuuagucgugcucccuguuA.....................................................................   17      1    seq
    ....................guguuuagucgugcucccuguuUu....................................................................   4       1    seq
    ....................guguuuagucgugcucccuguugA....................................................................   1       1    seq
    ....................guguuuagucgugcucccuguuAu....................................................................   1       1    seq
    ....................guguuuagucgugcucccuguugu....................................................................   1       0    seq
    ........................uagucgugcucccuguu.......................................................................   1       0    seq
    ........................................................cagggucugcgacggacacC....................................   2       1    seq
    ........................................................cagggucugcgacggacacu....................................   4       0    seq
    ........................................................cagggucugcgacggacacug...................................   6       0    seq
    ........................................................cagggucugcgacggacacugC..................................   3       1    seq
    ........................................................cagggucugcgacggacacuga..................................   1       0    seq
    ........................................................cagggucugcgacggacacugG..................................   1       1    seq
    ........................................................cagggucugcgacggacacugU..................................   23      1    seq
    .........................................................agggucugcgacggacacugG..................................   1       1    seq
    .........................................................agggucugcgacggacacugU..................................   6       1    seq
```

```
                    Mature                    Star
gcucaaugagcaguuagucuguguuuagucgugcucccuguuguguuuauauuuaacagggucugcgacggacacugauucgcugcuuacagucgccguuuaacauuugcua
.........................................................agggucugcgacggacacugUu.................................   1   1   seq
.............................................................................auucgcugcuuacagucgccguu............   1   0   seq
```

Provisional ID	:	JMRV_223
Score total	:	154.5
Score for star read(s)	:	3.9
Score for read counts	:	147
Score for mfe	:	2
Score for randfold	:	1.6
Score for cons. seed	:	
Total read count	:	300
Mature read count	:	288
Loop read count	:	0
Star read count	:	12

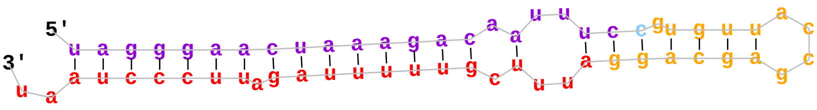

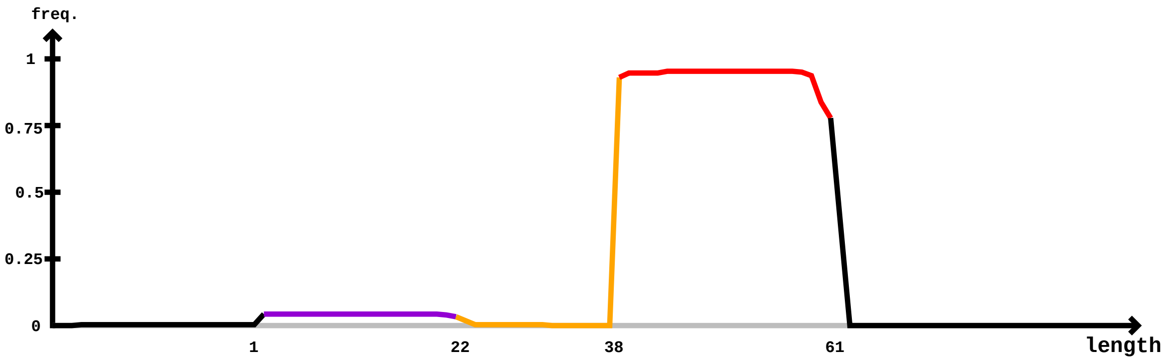


```
                    Star                                      Mature
5'- uugaaauuguuguaaugcuguagggaacuaaagacaauuuccguguuaccgagcaggauuucguuuuuagauucccuaaugcauugcaacgccaguucuucaacagggcuc   -3'    obs
    uugaaauuguuguaaugcuguagggaacuaaagacaauuuccguguuaccgagcaggauuucguuuuuagauucccuaaugcauugcaacgccaguucuucaacagggcuc          exp
    ........((((((((((..(((((((((((((((.(..(((.((((....)))))))..).)))))))).)))))))..))))))))))(((.(((....)))..)))..    reads     mm     sample
    .ugaaauuguuguaaugcug...........................................................................................    1         0      seq
    ....................uagggaacuaaagacaauu........................................................................    1         0      seq
    ....................uagggaacuaaagacaauuu.......................................................................    2         0      seq
    ....................uagggaacuaaagacaauuuc......................................................................    5         0      seq
    ....................uagggaacuaaagacaauuucc.....................................................................    4         0      seq
    ....................uagggaacuaaagacaauuuccguguuacc.............................................................    1         0      seq
    .........................................................auuucguuuuuagauuccc...................................    1         0      seq
    .........................................................auuucguuuuuagauucccu..................................    4         0      seq
    .........................................................auuucguuuuuagauucccua.................................    30        0      seq
    .........................................................auuucguuuuuagauucccuCa.................................    1         1      seq
    .........................................................auuucguuuuuagauucccuaU................................    2         1      seq
    .........................................................auuucguuuuuagauucccuaa................................    15        0      seq
    .........................................................auuucguuuuuagauucccuaau...............................    212       0      seq
    .........................................................auuucguuuuuagauucccuaaC...............................    10        1      seq
    .........................................................auuucguuuuuagauucccuCau...............................    1         1      seq
    .........................................................auuucguuuuuagauucccuaaA...............................    1         1      seq
    .........................................................auuucguuuuuagauucccuaUu...............................    1         1      seq
    .........................................................auuucguuuuuagauucccuaauU..............................    3         1      seq
    ..........................................................uuucguuuuuagauucccuaau...............................    5         0      seq
    ..............................................................guuuuuagauucccuaau...............................    2         0      seq
```

Provisional ID	:	JMRV_231
Score total	:	79.3
Score for star read(s)	:	3.9
Score for read counts	:	71.5
Score for mfe	:	2.3
Score for randfold	:	1.6
Score for cons. seed	:	
Total read count	:	152
Mature read count	:	128
Loop read count	:	0
Star read count	:	24

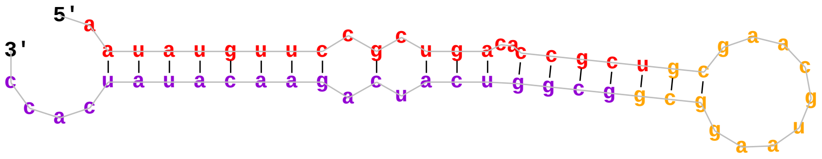

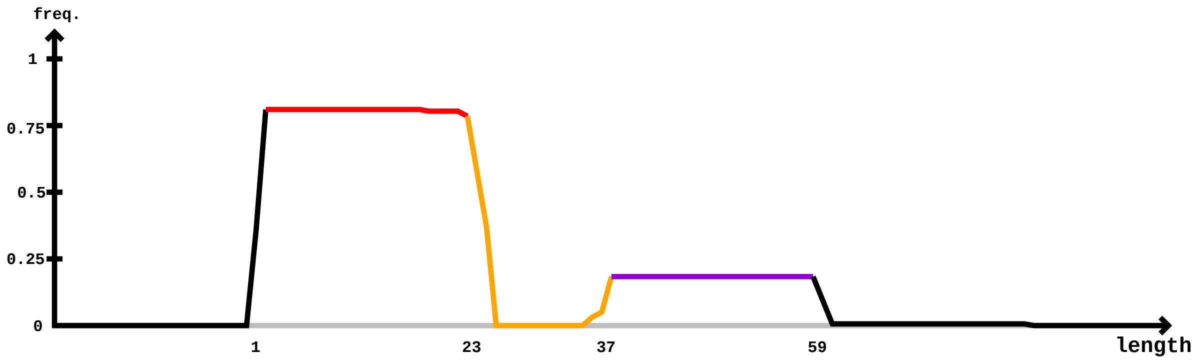

Mature **Star**

```
5'- cgagcccgggagcaggggagaauauguuccgcugacaccgcugcgaacguaaggcggcggucaucagaacauaucaccccccgcgcccgcggugccaucgccgggucacccgcg   -3'    obs
    cgagcccgggagcaggggagaauauguuccgcugacaccgcugcgaacguaaggcggcggucaucagaacauaucacccccgcgcccgcggugccaucgccgggucacccgcg          exp
    .(.(((((((.((.((((...((((((((.(.(((..(((((((.........)))))))))).).))))))))...)))).)).)))).))).)...(((.(((...))))))   reads   mm   sample
    ...................gaauauguuccgcugaca.............................................................................   1       0    seq
    ...................gaauauguuccgcugacaccgc.........................................................................   3       0    seq
    ...................gaauauguuccgcugacaccgcu........................................................................   13      0    seq
    ...................gaauauguuccgcugacaUcgcu........................................................................   1       1    seq
    ...................gaauauguuccgcugacaccgcC.......................................................................   2       1    seq
    ...................gaauauguuccgcugacaccgcuU.......................................................................   3       1    seq
    ...................gaauauguuccgcugacaccgcug.......................................................................   9       0    seq
    ...................gaauauguuccgcugacaccgcugU......................................................................   24      1    seq
    ...................gaauauguuccgcugacaccgcugc......................................................................   1       0    seq
    ....................aauauguuccgcugacaccgcu........................................................................   27      0    seq
    ....................aauauguuccgcugacaccgcC........................................................................   1       1    seq
    ....................aauauguuccgcugacaccgcG........................................................................   1       1    seq
    ....................aauauguuccgcugacaccgcuA.......................................................................   1       1    seq
    ....................aauauguuccgcugacaccgcuU.......................................................................   3       1    seq
    ....................aauauguuccgcugacaccgcug.......................................................................   5       0    seq
    ....................aauauguuccgcugacaccgcugA......................................................................   2       1    seq
    ....................aauauguuccgcugacaccAcugc......................................................................   1       1    seq
    ....................aauauguuccgcugacaccgcugU......................................................................   3       1    seq
    ....................aauauguuccgcugacaccgcugc......................................................................   27      0    seq
    ......................................................cggcggucaucagaacauaucacc....................................   5       0    seq
    .......................................................ggcggucaucagaacauaucacU....................................   1       1    seq
    .......................................................ggcggucaucagaacauaucacc....................................   2       0    seq
    ........................................................gcggucaucagaacauaucacc....................................   20      0    seq
    ........................................................gcggucaucagaacauaucacU....................................   1       1    seq
    ..............................................................................ccccgcgcccgcggugccaucg..............   1       0    seq
```

Provisional ID	:	JMRV_236
Score total	:	29.7
Score for star read(s)	:	3.9
Score for read counts	:	22
Score for mfe	:	2.2
Score for randfold	:	1.6
Score for cons. seed	:	
Total read count	:	55
Mature read count	:	44
Loop read count	:	0
Star read count	:	11

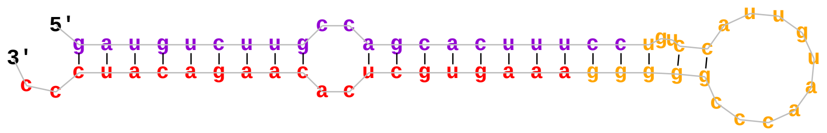

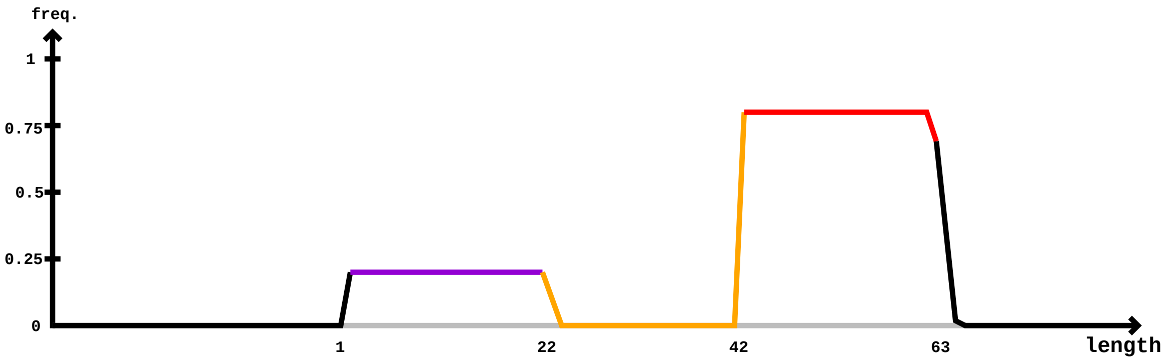

Star Mature

```
5'- ccucccguuccccgaccggcaaccccgucgaugucuugccagcacuuuccuguccauuguaacccgggggaaagugcucacaagacauccccggugagccagggggucagg   -3'    obs
    ccucccguuccccgaccggcaaccccgucgaugucuugccagcacuuuccuguccauuguaacccgggggaaagugcucacaagacauccccggugagccagggggucagg          exp
    ....((...((((....(((....(((..(((((((((..(((((((((((..((..........)))))))))))))..)))))))))..)))...)))..))))...))   reads     mm      sample
    .............................gaugucuugccagcacuuucc.............................................................   9         0       seq
    .............................gaugucuugccagcacuuuccu............................................................   2         0       seq
    ......................................................................aaagugcucacaagacaucc.....................   6         0       seq
    ......................................................................aaagugcucacaagacauccU....................   1         1       seq
    ......................................................................aaagugcucacaagacauccA....................   2         1       seq
    ......................................................................aaagugcucacaagacauccc....................   26        0       seq
    ......................................................................aaagugcucacaagacaucccU...................   5         1       seq
    ......................................................................aaagugcucacaagacaucccA...................   2         1       seq
    ......................................................................aaagugcucacaagacaucccc...................   1         0       seq
    ......................................................................aaagugcucacaagacauccccU..................   1         1       seq
```

Provisional ID	:	JMRV_217
Score total	:	17.8
Score for star read(s)	:	3.9
Score for read counts	:	10.3
Score for mfe	:	1.9
Score for randfold	:	1.6
Score for cons. seed	:	
Total read count	:	32
Mature read count	:	15
Loop read count	:	0
Star read count	:	17

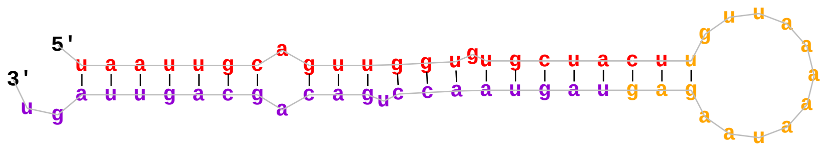

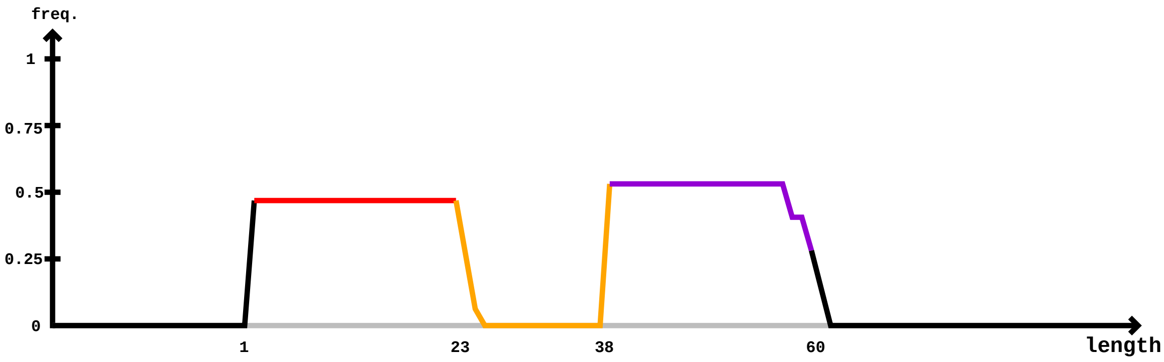

Mature Star

```
5'- gaaccggguuuagugcaauuaauugcaguuggugugcuacuuguuaaaaauaagaguaguaaccugacagcaguuaguugcacagaugcgccuaagcgcggugcgcguguu   -3'   obs
    gaaccggguuuagugcaauuaauugcaguuggugugcuacuuguuaaaaauaagaguaguaaccugacagcaguuaguugcacagaugcgccuaagcgcggugcgcguguu         exp
    ....((.((((.((((((((((((((.((((((.((((((((...........))))))))))).))).))))))))))))))))))).))....((((((.....))))))   reads   mm   sample
    ...................uaauugcaguuggugugcuacu......................................................................   12      0    seq
    ...................uaauugcaguuggugugcuacC......................................................................   1       1    seq
    ...................uaauugcaguuggugugcuacuuU....................................................................   2       1    seq
    ........................................................uaguaaccugacagcaguu....................................   4       0    seq
    ........................................................uaguaaccugacagcaguuag..................................   4       0    seq
    ........................................................uaguaaccugacagcaguuagC.................................   1       1    seq
    ........................................................uaguaaccugacagcaguuagu.................................   8       0    seq
```

Provisional ID	:	JMRV_219
Score total	:	16
Score for star read(s)	:	3.9
Score for read counts	:	8.3
Score for mfe	:	2.2
Score for randfold	:	1.6
Score for cons. seed	:	
Total read count	:	28
Mature read count	:	17
Loop read count	:	0
Star read count	:	11

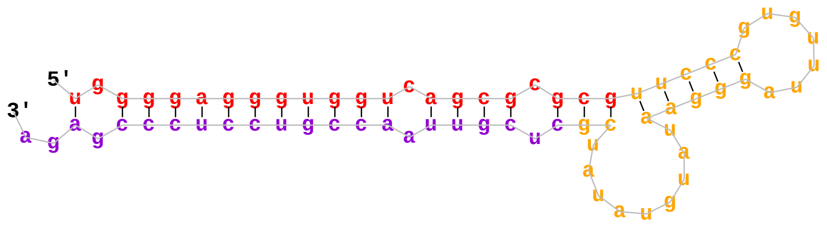

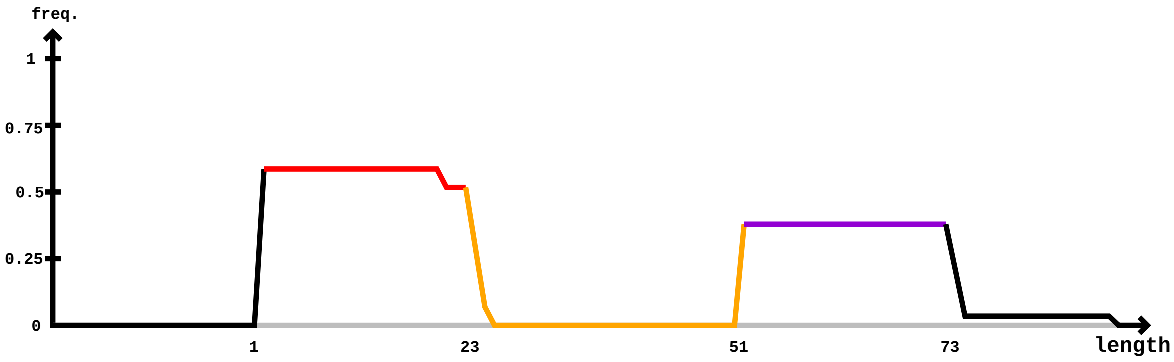


```
                         Mature                                                    Star
5'- cacaugcccguguugugauuuggggaggguggucagcgcgcguucccguguuuagggaauauguauaucgcucguuaaccguccucccgagauuacugcucgguguauuaaa   -3'    obs
    cacaugcccguguugugauuuggggaggguggucagcgcgcguucccguguuuagggaauauguauaucgcucguuaaccguccucccgagauuacugcucgguguauuaaa          exp
    ...((((((.((.(((((((.(((((((((((.((((.((((((((.......)))))).........))).)))).))))))))))).))))))).)).))).))))....   reads      mm         sample
    ....................uggggaggguggucagcgc.........................................................................   2          0          seq
    ....................uggggaggguggucagcgcgcg......................................................................   9          0          seq
    ....................uggggaggguggucagcgcgcgu.....................................................................   4          0          seq
    ....................uggggaggguggucagcgcgcguu....................................................................   2          0          seq
    ......................................................................cucguuaaccguccucccgagU....................   4          1          seq
    ......................................................................cucguuaaccguccucccgagG....................   1          1          seq
    ......................................................................cucguuaaccguccucccgaga....................   3          0          seq
    ......................................................................cucguuaaccguccucccgagau...................   1          0          seq
    ......................................................................cucguuaaccguccucccgagaA...................   2          1          seq
    ............................................................................................uuacugcucgguguauu...   1          0          seq
```

Provisional ID	:	JMRV_227
Score total	:	10.9
Score for star read(s)	:	3.9
Score for read counts	:	3.2
Score for mfe	:	2.2
Score for randfold	:	1.6
Score for cons. seed	:	
Total read count	:	18
Mature read count	:	16
Loop read count	:	0
Star read count	:	2

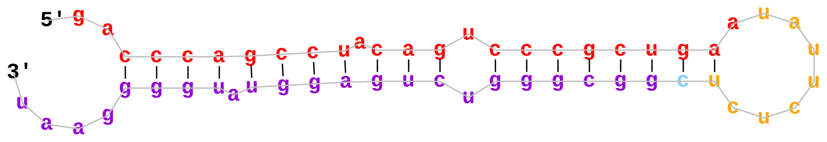

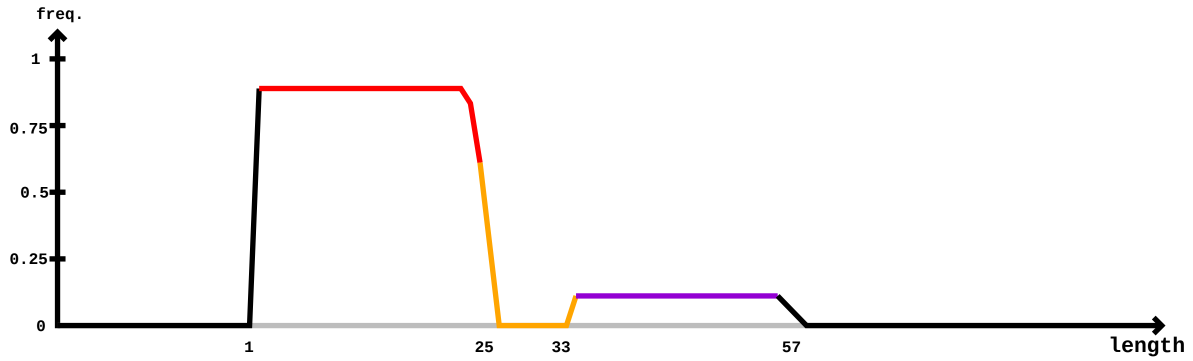

Mature **Star**

```
5'- ccuaccucuauccugggaggacccagccuacagucccgcugaauauucucucggcgggucugagguauggggaauccccgaauagaccguucacauagcaagcaugugggcuu   -3'   obs
    ccuaccucuauccugggaggacccagccuacagucccgcugaauauucucucggcgggucugagguauggggaauccccgaauagaccguucacauagcaagcaugugggcuu         exp
    ......(((((.(.((((...((((((((.(((.((((((((........)))))))).))))))).))))...)))).).)))))..((((((((.......))))))))..   reads   mm   sample
    ...................gacccagccuacagucccgcug........................................................................   1       0    seq
    ...................gacccagccuacagucccgcugU.......................................................................   1       1    seq
    ...................gacccagccuacagucccgcuga.......................................................................   3       0    seq
    ...................gacccagccuacagucccgcugaC......................................................................   1       1    seq
    ...................gacccagccuacagucccgcugaU......................................................................   6       1    seq
    ...................gacccagccuacagucccgcugaUu.....................................................................   2       1    seq
    ...................gacccagccuacagucccgcugaau.....................................................................   1       0    seq
    ...................gacccagccuacagucccgcugaCu.....................................................................   1       1    seq
    ....................................................ggcgggucugagguauggggaUu......................................   2       1    seq
```

Provisional ID	:	JMRV_105
Score total	:	6.1
Score for star read(s)	:	3.9
Score for read counts	:	-1.3
Score for mfe	:	2
Score for randfold	:	1.6
Score for cons. seed	:	
Total read count	:	9
Mature read count	:	8
Loop read count	:	0
Star read count	:	1

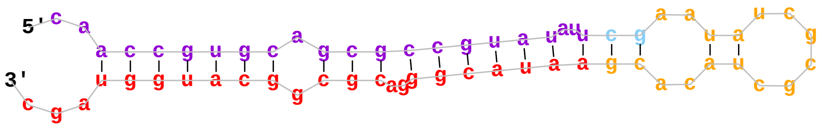

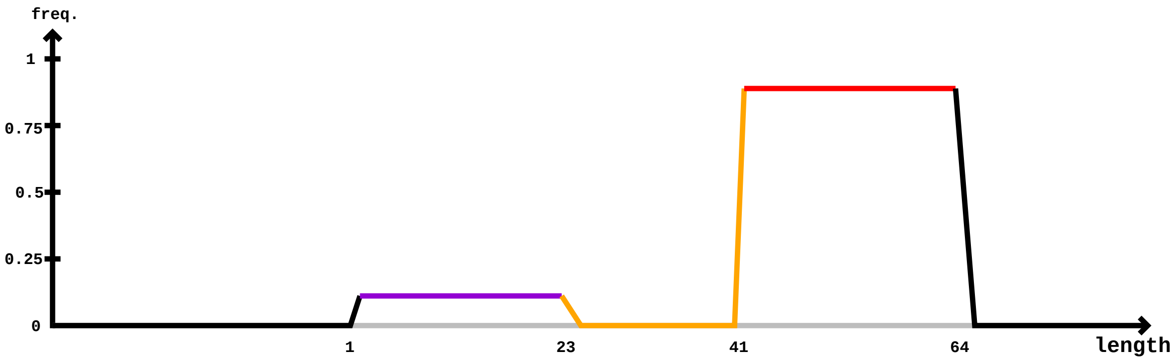

Star Mature

```
5'- ugguuagcgcaccagcguccaaccgcccgccaaccgugcagcgccguauauucgaauaucgcgcuacacgaauacgggacgcggcaugguagcgggagcuuggcauugcgca   -3'    obs
    ugguuagcgcaccagcguccaaccgcccgccaaccgugcagcgccguauauucgaauaucgcgcuacacgaauacgggacgcggcaugguagcgggagcuuggcauugcgca          exp
    ((((......))))((((((((.(.(((((..(((((((.(((((((((..(((..((......))..)))))))))..))).))))))).))))).).))))....)))).   reads    mm    sample
    ..............................caaccgugcagcgccguauauu............................................................   1        0     seq
    ......................................................................aauacgggacgcggcaugguagU...................   1        1     seq
    ......................................................................aauacgggacgcggcaugguagc...................   6        0     seq
    ......................................................................aauacgggacgcggcaugguagcU..................   1        1     seq
```

Provisional ID	:	JMRV_113
Score total	:	5.6
Score for star read(s)	:	3.9
Score for read counts	:	-2.3
Score for mfe	:	2.5
Score for randfold	:	1.6
Score for cons. seed	:	
Total read count	:	7
Mature read count	:	5
Loop read count	:	0
Star read count	:	2

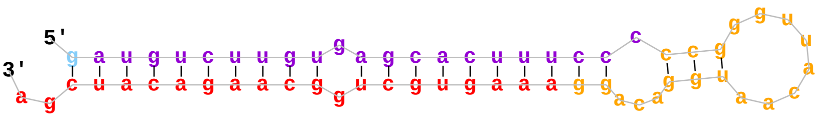

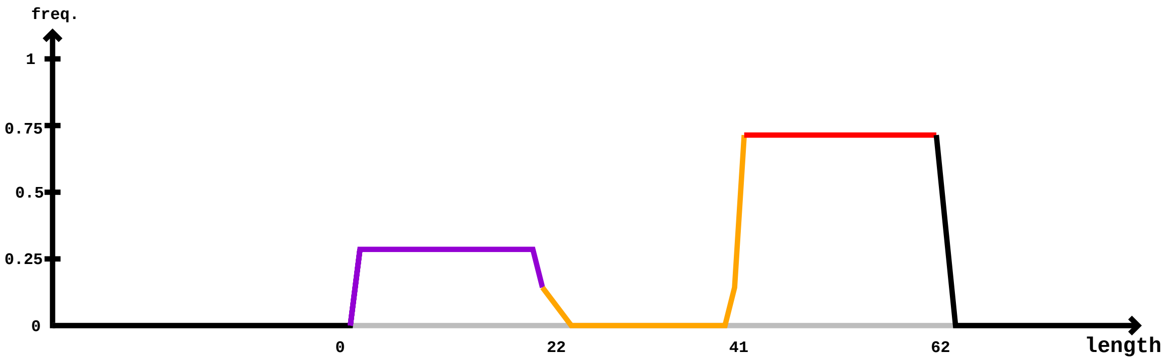

Star Mature

```
5'- cggcguuccugacccccuggcucaccggggaugucuugugagcacuuucccccggguuacaauggacaggaaagugcuggcaagacaucgacgggguugccggucggggaa   -3'    obs
    cggcguuccugacccccuggcucaccggggaugucuugugagcacuuucccccggguuacaauggacaggaaagugcuggcaagacaucgacgggguugccggucggggaa          exp
    .....((((((((....(((...(((..((((((((((.((((((((((.(((........)))...)))))))))).))))))))))..)))....))))))))))).   reads    mm     sample
    ..............................augucuugugagcacuuuc..............................................................   1        0      seq
    ..............................augucuugugagcacuuuccU............................................................   1        1      seq
    .....................................................................gaaagugcuggcaagacaucga....................   1        0      seq
    ......................................................................aaagugcuggcaagacaucga....................   3        0      seq
    ......................................................................aaagugcuggcaagacaucgaU...................   1        1      seq
```

Provisional ID	:	JMRV_233
Score total	:	2.8
Score for star read(s)	:	-1.3
Score for read counts	:	0
Score for mfe	:	2.5
Score for randfold	:	1.6
Score for cons. seed	:	
Total read count	:	40
Mature read count	:	40
Loop read count	:	0
Star read count	:	0

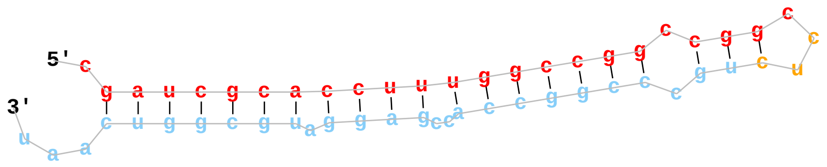

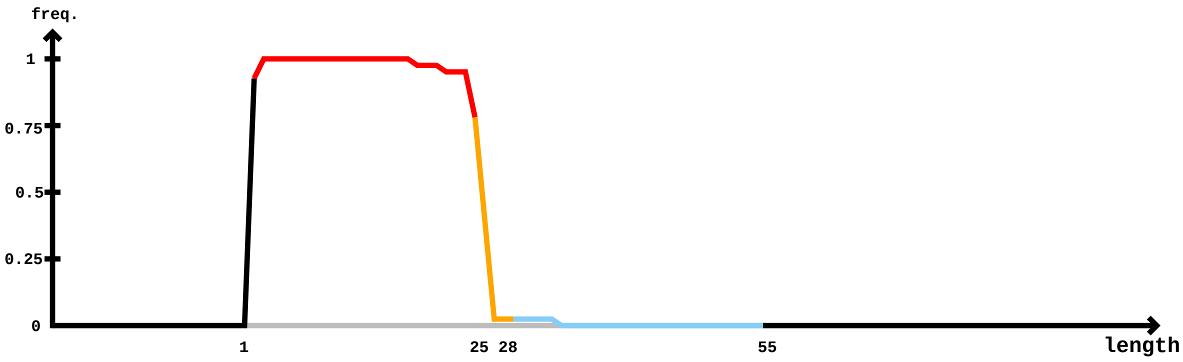


```
                  Mature              Star
5'- acgggugggguuagaccccgcgaucgcaccuuuggccggccggccucugcccggccaccgaggaugcggucaaugggucgaacccgccggcgucaacgucgcagccggccuagc   -3'    exp
    ......(((((.(((((...((((((((((((((((.(((...))).)))))))..)))).)))))))...))))).)))))((((((...........))))))..... reads      mm        sample
    ..................cgaucgcaccuuuggcc.............................................................................. 1          0         seq
    ..................cgaucgcaccuuuggccggc........................................................................... 1          0         seq
    ..................cgaucgcaccuuuggccggccgg........................................................................ 5          0         seq
    ..................cgaucgcaccuuuggccggccggA....................................................................... 2          1         seq
    ..................cgaucgcaccuuuggccggccggU....................................................................... 28         1         seq
    ..................cgaucgcaccuuuggccggccggccucugccc............................................................... 1          0         seq
    ...................gaucgcaccuuuggccggccgg........................................................................ 2          0         seq
    ...................gaucgcaccuuuggccggccggU....................................................................... 1          1         seq
```

Provisional ID : JMRV_226
Score total : 2.5
Score for star read(s) : -1.3
Score for read counts : 0
Score for mfe : 2.2
Score for randfold : 1.6
Score for cons. seed :
Total read count : 1441
Mature read count : 1430
Loop read count : 0
Star read count : 11

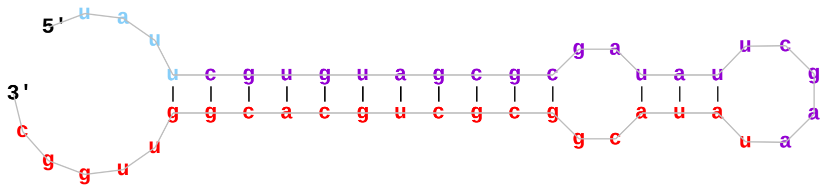

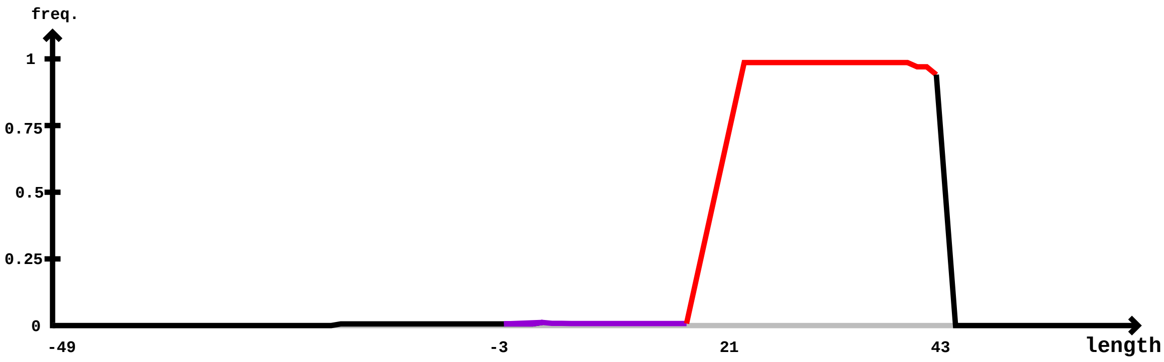

Star Mature

```
5'- uggguauugcgcaaugccaagcucccgcuaccaugccgcgucccguauucguguagcgcgauauucgaauauacggcgcugcacgguuggcgggcgguuggacgcuggugc  -3'  obs
    uggguauugcgcaaugccaagcucccgcuaccaugccgcgucccguauucguguagcgcgauauucgaauauacggcgcugcacgguuggcgggcgguuggacgcuggugc       exp
    ..((((((....))))))..((.((.((..(((.(((((...((((..(((((((((((..(((......)))..)))))))))))...))))))))))))..)).)).))  reads  mm  sample
    ............................uaccaugccgcgucccguauu..............................................................  2     0   seq
    ............................uaccaugccgcgucccguauuc.............................................................  6     0   seq
    ............................uaccaugccgcgucccguauucgu...........................................................  1     0   seq
    .................................................cguguagcgcgauauucgaa..........................................  10    0   seq
    ..................................................guguagcgcgauauucgaa..........................................  1     0   seq
    ...................................................................aauauacggcgcugcacgguugg.....................  1     0   seq
    ...................................................................aauauacggcgcugcacgguugA.....................  1     1   seq
    ...................................................................aauauacggcgcugcacgguuggc....................  1     0   seq
    ....................................................................auauacggcgcugcacgguugg.....................  3     0   seq
    ....................................................................auauacggcgcugcacgguuggU....................  5     1   seq
    ....................................................................auauacggcgcugcacgguuggA....................  1     1   seq
    ....................................................................auauacggcgcugcacgguuggc...................  52    0   seq
    ....................................................................auauacggcgcugcacgguuggcU..................  1     1   seq
    ....................................................................auauacggcgcugcacgguuggcA..................  1     1   seq
    .....................................................................uauacggcgcugcacgguu.......................  22    0   seq
    .....................................................................uauacggcgcugcacgguC.......................  1     1   seq
    .....................................................................uauacggcgcugcacgguugU.....................  2     1   seq
    .....................................................................uauacggcgcugcacgguugA.....................  4     1   seq
    .....................................................................uauacggcgcugcacgguugg.....................  32    0   seq
    .....................................................................uauacggcgcugcacgguuggU....................  65    1   seq
    .....................................................................uauacggcgcugcacgguCggc....................  3     1   seq
    .....................................................................uauacggcgcugcacgguuCgc....................  1     1   seq
    .....................................................................uauacggcgcugcacgguuggA....................  10    1   seq
    .....................................................................uauacggcgcugcacgguGggc....................  1     1   seq
    .....................................................................uauacggcgcugcacgguugCc....................  2     1   seq
    .....................................................................uauacggcgcugcacgguuggc....................  1179  0   seq
    .....................................................................uauacggcgcugcacgguuggG....................  1     1   seq
    .....................................................................uauacggcgcugcacgguugAc....................  1     1   seq
    .....................................................................uauacggcgcugcacgguuggcC...................  2     1   seq
    .....................................................................uauacggcgcugcacgguuggcA...................  15    1   seq
    .....................................................................uauacggcgcugcacgguuggcg...................  3     0   seq
    .....................................................................uauacggcgcugcacgguuggcU...................  19    1   seq
    ......................................................................auacggcgcugcacgguuggc....................  1     0   seq
```

Star **Mature**

uggguauugcgcaaugccaagcucccgcuaccaugccgcgucccguauucguguagcgcgauauucgaauauacggcgcugcacgguuggcgggcgguuggacgcuggugc

Provisional ID	:	JMRV_215
Score total	:	2.4
Score for star read(s)	:	-1.3
Score for read counts	:	0
Score for mfe	:	2.1
Score for randfold	:	1.6
Score for cons. seed	:	
Total read count	:	76
Mature read count	:	59
Loop read count	:	0
Star read count	:	17

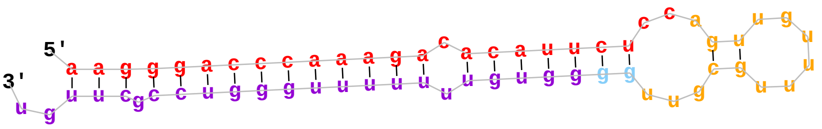

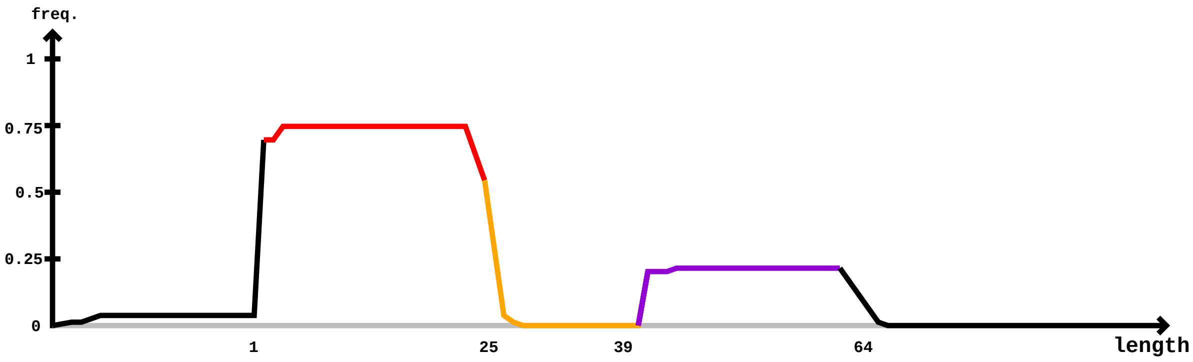

Mature Star

```
5'- ggaauuucauugcgacguggaagggacccaaagacacauucuccaguuguuuugcguuggggguguuuuuuuggguccgcuuguaugucggaauguaacuucggcggcuaauuuu  -3'  obs
    ggaauuucauugcgacguggaagggacccaaagacacauucuccaguuguuuugcguuggggguguuuuuuuggguccgcuuguaugucggaauguaacuucggcggcuaauuuu        exp
    .(((.((((((.(((((((.((((((((((((((.(((((((...((......))...))))))).))))))))))).))).))))))).)))).)).)))............  reads   mm   sample
    ggaauuucauugcgacgugg..............................................................................................  1       0    seq
    ..aauuucauugcgacgugg..............................................................................................  1       0    seq
    ...auuucauugcgacgugg..............................................................................................  1       0    seq
    ....................aagggacccaaagacacauucC........................................................................  1       1    seq
    ....................aagggacccaaagacacauucu........................................................................  6       0    seq
    ....................aagggacccaaagacacauucA........................................................................  1       1    seq
    ....................aagggacccaaagacacauucuc.......................................................................  8       0    seq
    ....................aagggacccaaagacacauucCcc......................................................................  1       1    seq
    ....................aagggacccaaagacacauucucA......................................................................  1       1    seq
    ....................aagggacccaaagacacauucucc......................................................................  35      0    seq
    ....................aagggacccaaagacacauucuccaU....................................................................  1       1    seq
    ....................aagggacccaaagacacauucuccagu...................................................................  1       0    seq
    ......................gggacccaaagacacauucucc......................................................................  1       0    seq
    ......................gggacccaaagacacauucucca.....................................................................  1       0    seq
    ......................gggacccaaagacacauucuccG.....................................................................  1       1    seq
    ......................gggacccaaagacacauucuccaA....................................................................  1       1    seq
    ............................................................gguguuuuuuuggguccgcuug................................  1       0    seq
    ............................................................gguguuuuuuuggguccgcuugC...............................  4       1    seq
    ............................................................gguguuuuuuuggguccgcuugu...............................  10      0    seq
    ............................................................gguguuuuuuuggguccgcuuguaG.............................  1       1    seq
    ...............................................................guuuuuuuggguccgcuuguU..............................  1       1    seq
```

Provisional ID	:	JMRV_213
Score total	:	2.3
Score for star read(s)	:	-1.3
Score for read counts	:	0
Score for mfe	:	2
Score for randfold	:	1.6
Score for cons. seed	:	
Total read count	:	492
Mature read count	:	491
Loop read count	:	0
Star read count	:	1

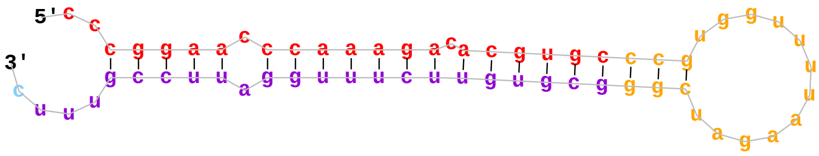

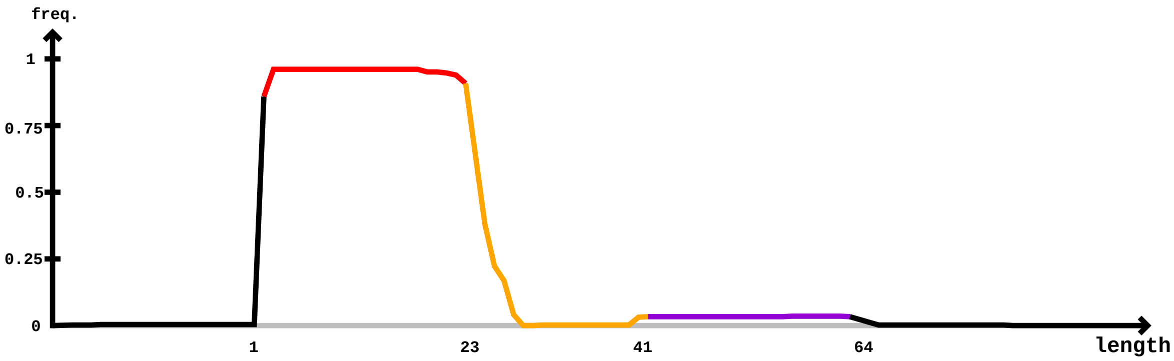

Mature Star

```
5'- cgaacuuccaucguaacgcccccggaacccaaagacacgugcccgugguuuuaagaucgggcguguucuuuggauuccguuucguugcgaugugcauuaaccgcgaggggguu -3'  obs
    cgaacuuccaucguaacgcccccggaacccaaagacacgugcccgugguuuuaagaucgggcguguucuuuggauuccguuucguugcgaugugcauuaaccgcgaggggguu       exp
    ........(((((((((....(((((.(((((((.(((((((((...........))))))))))))))))).)))))...)))))))))......((((......))))   reads  mm  sample
    cgaacuuccaucguaacgcc.............................................................................................   1      0   seq
    ...acuuccaucguaacgcc.............................................................................................   1      0   seq
    ....................cccggaacccaaagaca............................................................................   5      0   seq
    ....................cccggaacccaaagacacg..........................................................................   2      0   seq
    ....................cccggaacccaaagacacgu.........................................................................   3      0   seq
    ....................cccggaacccaaagacacgug........................................................................   14     0   seq
    ....................cccggaacccaaagacacguC........................................................................   1      1   seq
    ....................cccggaacccaaagacacgGg........................................................................   1      1   seq
    ....................cccggaacccaaagacacgugc.......................................................................   124    0   seq
    ....................cccggaacccaaagacacgCgc.......................................................................   1      1   seq
    ....................cccggaacccaaagacacAugc.......................................................................   1      1   seq
    ....................cccggaacccaaagacacgugU.......................................................................   8      1   seq
    ....................cccggaacccaaagacacgugA.......................................................................   3      1   seq
    ....................cccggaacccaaagacacgugcA......................................................................   2      1   seq
    ....................cccggaacccaaagacacgCgcc......................................................................   1      1   seq
    ....................cccggaacccaaagacacgugUc......................................................................   1      1   seq
    ....................cccggaacccaaagacacgugcc......................................................................   108    0   seq
    ....................cccggaacccaaagacacgugcU......................................................................   9      1   seq
    ....................cccggaacccaaagacacgugccc.....................................................................   67     0   seq
    ....................cccggaacccaaagacacgugccA.....................................................................   2      1   seq
    ....................cccggaacccaaagacacgugccU.....................................................................   7      1   seq
    ....................cccggaacccaaagacacgugcccU....................................................................   4      1   seq
    ....................cccggaacccaaagacacgugcccg....................................................................   16     0   seq
    ....................cccggaacccaaagacacgugcccA....................................................................   1      1   seq
    ....................cccggaacccaaagacacgugcccgC...................................................................   4      1   seq
    ....................cccggaacccaaagacacgugcccgG...................................................................   1      1   seq
    ....................cccggaacccaaagacacgugcccAu...................................................................   1      1   seq
    ....................cccggaacccaaagacacgugcccgu...................................................................   35     0   seq
    ....................cccggaacccaaagacacgugcccUu...................................................................   4      1   seq
    ....................cccggaacccaaagacacgugcccguC..................................................................   2      1   seq
    ....................cccggaacccaaagacacgugcccguU..................................................................   11     1   seq
    .....................ccggaacccaaagacacgu.........................................................................   1      0   seq
    .....................ccggaacccaaagacacgugU.......................................................................   1      1   seq
```

```
                                Mature                                  Star
cgaacuuccaucguaacgcccccggaacccaaagacacgugcccgugguuuuaagaucgggcguguucuuuggauuccguuucguugcgaugugcauuaaccgcgagggguu
.....................ccggaacccaaagacacgugc......................................................................	2	0	seq
.....................ccggaacccaaagacacgugcc.....................................................................	6	0	seq
.....................ccggaacccaaagacacgugUc.....................................................................	1	1	seq
.....................ccggaacccaaagacacgugcU.....................................................................	1	1	seq
.....................ccggaacccaaagacacgugccU....................................................................	2	1	seq
.....................ccggaacccaaagacacgugccc....................................................................	3	0	seq
.....................ccggaacccaaagacacgugcccg...................................................................	6	0	seq
.....................ccggaacccaaagacacgugcccU...................................................................	1	1	seq
.....................ccggaacccaaagacacgugcccgA..................................................................	1	1	seq
.....................ccggaacccaaagacacgugcccgu..................................................................	18	0	seq
.....................ccggaacccaaagacacgugcccUu..................................................................	1	1	seq
.....................ccggaacccaaagacacgugcccguU.................................................................	8	1	seq
.................................................uuuaagaucgggcguguucuuuggauuccguuu..............................	1	0	seq
...........................................................ggcguguucuuuggauuccguu...............................	1	0	seq
...........................................................ggcguguucuuuggauuccguuC..............................	1	1	seq
...........................................................ggcguguucuuuggauuccguCu..............................	1	1	seq
...........................................................ggcguguucuuuggauuccguuu..............................	11	0	seq
...........................................................ggcguguucuuuggauuccguuuU.............................	1	1	seq
............................................................gcguguucuuuggauuccguuu..............................	1	0	seq
...........................................................................uccguuucguugcgaugugcauu..............	1	0	seq
```

Provisional ID	:	JMRV_230
Score total	:	1.1
Score for star read(s)	:	-1.3
Score for read counts	:	-1.3
Score for mfe	:	2.2
Score for randfold	:	1.6
Score for cons. seed	:	
Total read count	:	9
Mature read count	:	9
Loop read count	:	0
Star read count	:	0

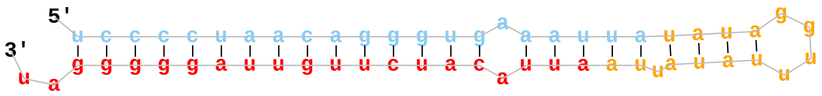

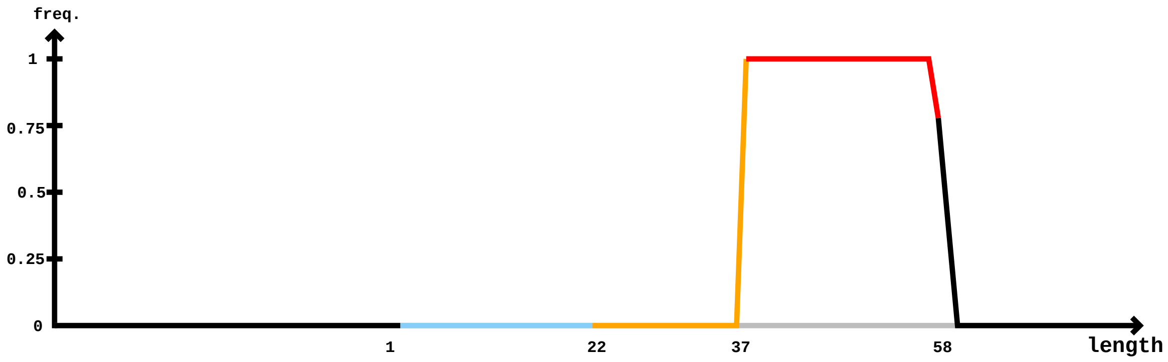


```
                                     Star                                    Mature
5'- guaauccuauauuaauacagguacaaacggggauuccccuaacagggugaaauuauauagguuuauauuaauuacaucuuguuagggggauccccgguaccuaggucucca   -3'    exp
    ..................((((((...((((((((((((((((((((((.(((((((((....)))).))))).))))))))))))))))))))))))))))).........    reads       mm          sample
    ......................................................................auuacaucuuguuaggggga.....................    2           0           seq
    ......................................................................auuacaucuuguuagggggau....................    6           0           seq
    ......................................................................auuacaucuuguuaggggGu....................    1           1           seq
```
