# Supplementary material for: Identification of virus-encoded microRNAs in divergent Papillomaviruses
Source: PLoS Pathog. 2018 Jul 26;14(7):e1007156. doi: 10.1371/journal.ppat.1007156 (PMC6062147; doi:10.1371/journal.ppat.1007156)
Supplement: S4 Dataset — This dataset shows miRDeep2’s primary output (in PDF format) of read coverage along hairpin structures for all predictions made by the pipeline for our PV miDGE data (including the two PyV positive controls). Provisional IDs for the individual pre-miRNAs are given as assigned by the pipeline, see S2 Dataset for matching these IDs to known or novel pre-miRNAs. (PDF) [file ppat.1007156.s011.pdf]

Provisional ID : FcPV1_24
Score total : 3541.5
Score for star read(s) : 3.9
Score for read counts : 3538.3
Score for mfe : 1.5
Score for randfold : -2.2
Score for cons. seed :
Total read count : 6952
Mature read count : 3491
Loop read count : 0
Star read count : 3461

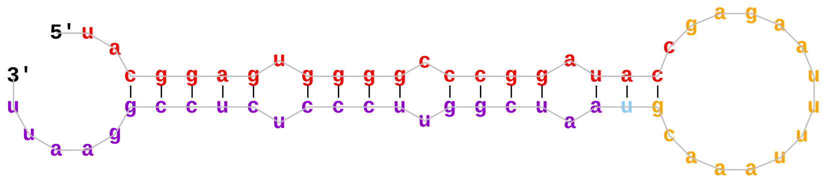

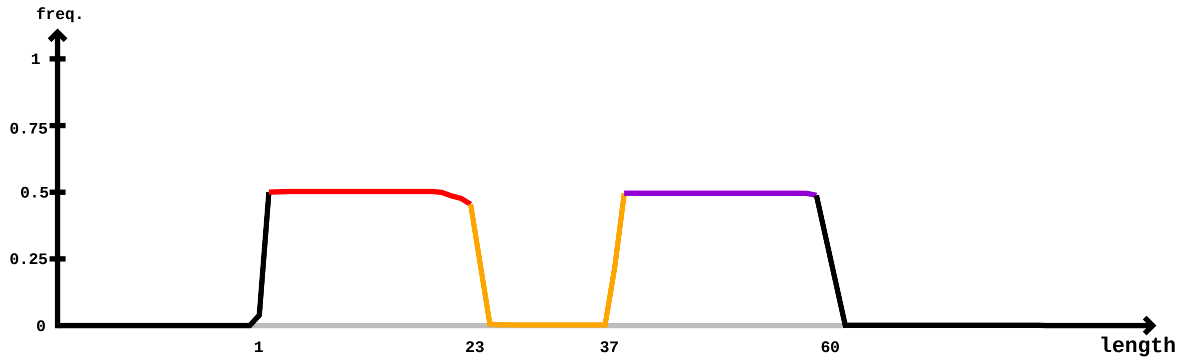

Mature Star

```
5'- ccgugucuugggcaugacguuacggaguggggcccggauaccgagaauuuuaaacguaaucgguucccucuccggaauuucagccuuggaaccuccuccgaucgagcuccga -3'  obs
    ccgugucuugggcaugacguuacggaguggggcccggauaccgagaauuuuaaacguaaucgguucccucuccggaauuucagccuuggaaccuccuccgaucgagcuccga       exp
    .........((((.(((.((..(((((.((((.((((.(((..............))).)))).)))).)))))..)).)))))))((((..(((........))).)))).  reads  mm  sample
    ...................uuacggaguggggcccgga..........................................................................  10     0   seq
    ...................uuacggaguggggcccggau.........................................................................  41     0   seq
    ...................uuacggaguggggcccggaua........................................................................  55     0   seq
    ...................uuacggaguggggcccggauac.......................................................................  27     0   seq
    ...................uuacggaguggggcccggauacc......................................................................  38     0   seq
    ...................uuacggaguggggcccggauacU......................................................................  76     1   seq
    ...................uuacggaguggggcccggauacA......................................................................  11     1   seq
    ...................uuacggaguggggcccggauacG......................................................................  2      1   seq
    ...................uuacggaguggggcccggauaccgU....................................................................  10     1   seq
    ...................uuacggaguggggcccggauaccgagaauuuuaaacguaU.....................................................  1      1   seq
    ....................uacggaguggggcccgga..........................................................................  13     0   seq
    ....................uacggaguggggcccggaA.........................................................................  6      1   seq
    ....................uacggaguggggcccggau.........................................................................  36     0   seq
    ....................uacggaguggggcccggaAa........................................................................  3      1   seq
    ....................uacggaguggggcccggaua........................................................................  7      0   seq
    ....................uacggaguggggcccggauac.......................................................................  128    0   seq
    ....................uacggaguggggcccggaNacc......................................................................  3      1   seq
    ....................uacggaguggggcccggaAacc......................................................................  1      1   seq
    ....................uacggaguggggcccggauaAc......................................................................  1      1   seq
    ....................uacggaguggggcccggauGcc......................................................................  5      1   seq
    ....................uacggaguggggcccggauNcc......................................................................  1      1   seq
    ....................uacggaguggggcccggauacU......................................................................  57     1   seq
    ....................uacggaguggggcccggauacc......................................................................  2744   0   seq
    ....................uacggaguggggcccggauaccg.....................................................................  17     0   seq
    ....................uacggaguggggcccggauaccU.....................................................................  172    1   seq
    ....................uacggaguggggcccggauaccgU....................................................................  2      1   seq
    ....................uacggaguggggcccggauaccga....................................................................  7      0   seq
    ....................uacggaguggggcccggauaccgaAa..................................................................  4      1   seq
    ....................uacggaguggggcccggauaccgagaauuuuaaacg........................................................  4      0   seq
    ....................uacggaguggggcccggauaccgagaauuuuaCacg........................................................  3      1   seq
    ....................uacggaguggggcccggauaccgCgaauuuuaaacg........................................................  1      1   seq
    ....................uacggaguggggcccggauaccgagaauuuuaaacgu.......................................................  2      0   seq
    ....................uacggaguggggcccggauaccgCgaauuuuaaacgu.......................................................  2      1   seq
```

```
                    Mature                             Star
ccgugucuugggcaugacguuacggaguggggcccggauaccgagaauuuuaaacguaaucgguucccucuccggaauuucagccuuggaaccuccuccgaucgagcuccga
.....................acggaguggggcccggauacc......................................................................	5	0	seq
.....................acggaguggggcccggauaccgagaauuuuaaacg........................................................	3	0	seq
......................cggaguggggcccggau.........................................................................	9	0	seq
........................................................uaaucgguucccucuccgga....................................	2	0	seq
........................................................uaaucgguucccucuccggaG...................................	2	1	seq
........................................................uaaucgguucccucuccggGa...................................	1	1	seq
........................................................uaaucgguucccucuccggaa...................................	35	0	seq
........................................................uaaucgguucccucuccggaCu..................................	1	1	seq
........................................................uaaucgguucccucuccgAaau..................................	1	1	seq
........................................................uaaucgguucccucuccggaau..................................	832	0	seq
........................................................uaaucgguucccucuccggaaC..................................	1	1	seq
........................................................uaaucgguucccucuccggGau..................................	2	1	seq
........................................................uaaucgguucccucuccggaauG.................................	2	1	seq
........................................................uaaucgguucccucuccggaauA.................................	28	1	seq
........................................................uaaucgguucccucuccggaauu.................................	607	0	seq
........................................................uaaucgguucccucuccggaUuu.................................	1	1	seq
.........................................................aaucgguucccucuccgga....................................	2	0	seq
.........................................................aaucgguucccucuccggaa...................................	11	0	seq
.........................................................aaucgguucccucuccggaaC..................................	1	1	seq
.........................................................aaucgguucccucuccggaaG..................................	1	1	seq
.........................................................aaucgguucccucuccggaGu..................................	1	1	seq
.........................................................aaucgguucccucuccggGau..................................	1	1	seq
.........................................................aaucgguucccucuccggaau..................................	732	0	seq
.........................................................aaucgguucccucuccggaauG.................................	1	1	seq
.........................................................aaucgguucccucuccggaGuu.................................	1	1	seq
.........................................................aaucgguucccucuccggaauu.................................	1188	0	seq
.........................................................aaucgguucccucuccggaauA.................................	5	1	seq
.........................................................aaucgguucccucuccggaaAu.................................	1	1	seq
.........................................................aaucgguucccucuccggaUuu.................................	1	1	seq
...............................................................................ucagccuuggaaccuccuccga...........	8	0	seq
```

Provisional ID	:	FcPV1_25
Score total	:	676.9
Score for star read(s)	:	3.9
Score for read counts	:	669.5
Score for mfe	:	1.8
Score for randfold	:	1.6
Score for cons. seed	:	
Total read count	:	1325
Mature read count	:	1296
Loop read count	:	0
Star read count	:	29

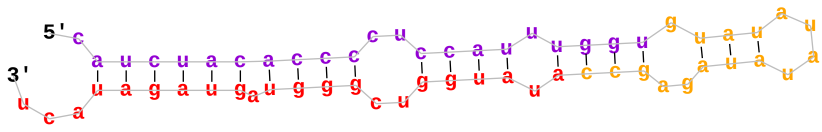

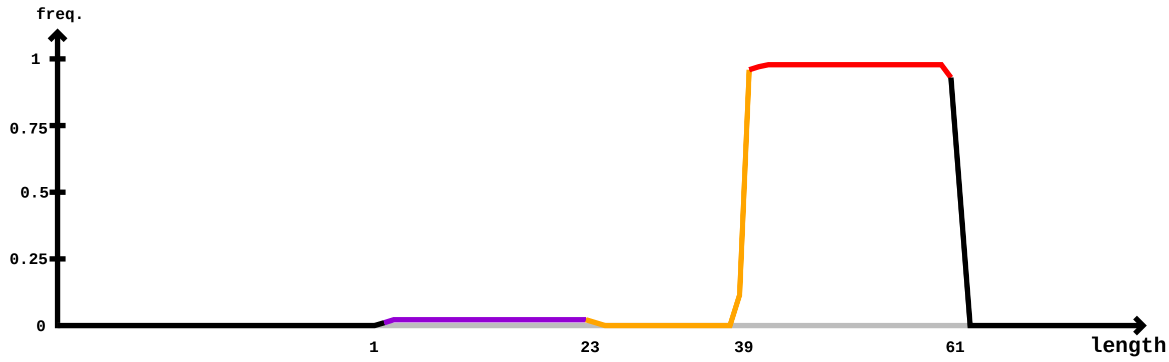

Star **Mature**

```
5'- uagccgcugucuugauuaucugccauuuggaucaucuacaccccuccauuugguguauauauauagagccauauggucggguaguagauacuccaauuggcgguucgcauc   -3'   obs
    uagccgcugucuugauuaucugccauuuggaucaucuacaccccuccauuugguguauauauauagagccauauggucggguaguagauacuccaauuggcgguucgcauc         exp
    ............((.....((((((.(((((..((((((((((..((((.((((.(((....)))..)))).))))..)))).))))))..))))).))))))....))..    reads   mm   sample
    ................................caucuacaccccuccauuuggu.........................................................    14      0    seq
    .................................aucuacaccccuccauuuggu.........................................................    10      0    seq
    .................................aucuacaccccuccauuugguA........................................................    5       1    seq
    .....................................................................cauauggucggguaguagauac....................    5       0    seq
    .....................................................................cauauggucggguaguagauacC...................    11      1    seq
    .....................................................................cauauggucggguaguagauacG...................    1       1    seq
    .....................................................................cauauggucggguaguagauacA...................    11      1    seq
    .....................................................................cauauggucggguaguagauacu...................    123     0    seq
    .....................................................................cauauggucggguaguagauCcu...................    1       1    seq
    ......................................................................auauggucggguaguagauac....................    57      0    seq
    ......................................................................auauggucggguaguagauacA...................    2       1    seq
    ......................................................................auauggucggguaguagauacC...................    202     1    seq
    ......................................................................auauggucggguaguagauacu...................    832     0    seq
    ......................................................................auauggucggguaguagauacG...................    1       1    seq
    ......................................................................auauggucggguaguagauGcu...................    3       1    seq
    ......................................................................auauggucggguaguagaCacu...................    1       1    seq
    ......................................................................auauggucggguaguagauacuU..................    21      1    seq
    .......................................................................uauggucggguaguagauac....................    2       0    seq
    .......................................................................uauggucggguaguagauacu...................    13      0    seq
    ........................................................................auggucggguaguagauacu...................    10      0    seq
```

Provisional ID	:	HPV37_872
Score total	:	106.3
Score for star read(s)	:	3.9
Score for read counts	:	99.5
Score for mfe	:	1.3
Score for randfold	:	1.6
Score for cons. seed	:	
Total read count	:	207
Mature read count	:	165
Loop read count	:	0
Star read count	:	42

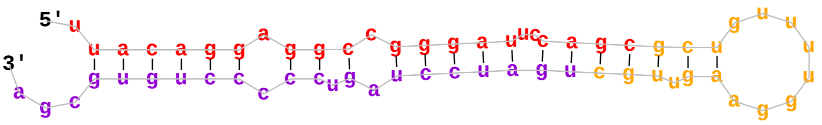

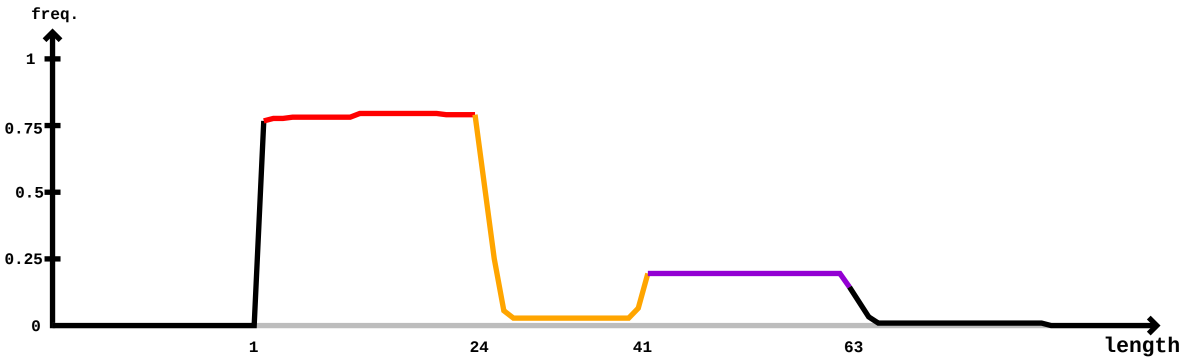

Mature Star

```
5'- ggauauugauacuccuguaguuacaggaggccgggauuccagcgcuguuuuggaaguugcugauccuaguccccugugcgaacaagaguuuccagaacacaauaucauaauc  -3'  obs
    ggauauugauacuccuguaguuacaggaggccgggauuccagcgcuguuuuggaaguugcugauccuaguccccugugcgaacaagaguuuccagaacacaauaucauaauc       exp
    .(((((((..((((.(((.((.(((((.(((.(((((..(((((((........)).)))))))))).).)).)))))))..))).)))).........)))))))......  reads   mm   sample
    ....................uuacaggaggccgggauuc.........................................................................  1       0    seq
    ....................uuacaggaggccgggauuccagU.....................................................................  2       1    seq
    ....................uuacaggaggccgggauuccagc.....................................................................  67      0    seq
    ....................uuacaggaggccgggauuccagcU....................................................................  1       1    seq
    ....................uuacaggaggccgggauuccagcA....................................................................  4       1    seq
    ....................uuacaggaggccgggauuccagcg....................................................................  42      0    seq
    ....................uuacaggaggccgggauuccagcgU...................................................................  26      1    seq
    ....................uuacaggaggccgggauuccagcgA...................................................................  15      1    seq
    ....................uuacaggaggccgggauuccagcgG...................................................................  1       1    seq
    ....................uuacaggaggccgggauuccagcgAu..................................................................  6       1    seq
    .....................uacaggaggccgggauuccagcgcuguuuuggaaguugc....................................................  2       0    seq
    .......................caggaggccgggauuccagcgcuguuuuggaaguugc....................................................  1       0    seq
    .............................ccgggauuccagcgcuguuuuggaaguugc....................................................  3       0    seq
    ...........................................................cugauccuaguccccugugcA................................  1       1    seq
    ...........................................................cugauccuaguccccugugcg................................  4       0    seq
    ...........................................................cugauccuaguccccugugcga...............................  3       0    seq
    ............................................................ugauccuaguccccugugcg................................  6       0    seq
    ............................................................ugauccuaguccccugugcga...............................  22      0    seq
    ............................................................ugauccuaguccccugugcgG...............................  1       1    seq
    ............................................................ugauccuaguccccugugcgaaA.............................  5       1    seq
    .................................................................................acaagaguuuccagaacaca...........  2       0    seq
```

Provisional ID	:	HPV41_974
Score total	:	50.5
Score for star read(s)	:	3.9
Score for read counts	:	44.5
Score for mfe	:	0.5
Score for randfold	:	1.6
Score for cons. seed	:	
Total read count	:	99
Mature read count	:	86
Loop read count	:	0
Star read count	:	13

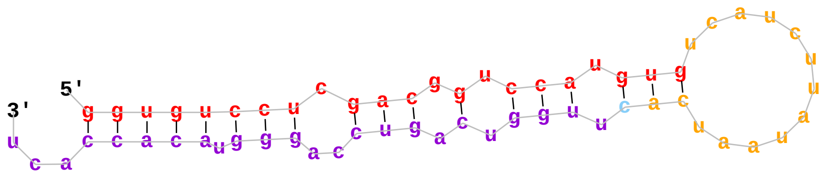

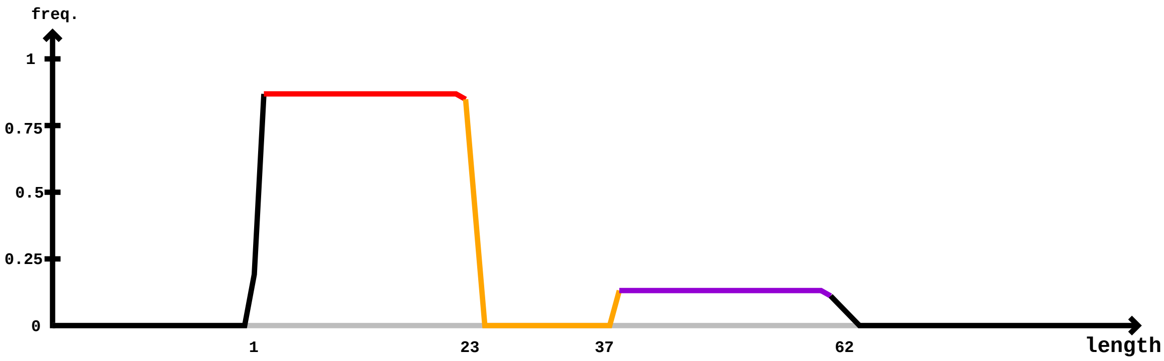

Mature Star

```
5'- aauugcugguauuguggugcgguguccucgacgguccaugugucaucuuauaaucacuuggucaguccaggguacaccacuccauuaucuauuuacuucgcauguauuucu  -3'   obs
    aauugcugguauuguggugcgguguccucgacgguccaugugucaucuuauaaucacuuggucaguccaggguacaccacuccauuaucuauuuacuucgcauguauuucu         exp
    ...(((.((((..((((.(.((((((((.(((.(.(((.(((...........))).))).).)))..))).))))).).)))).......))))..))).........   reads   mm   sample
    ...................cgguguccucgacgguccauguA.....................................................................   9       1    seq
    ...................cgguguccucgacgguccaugug.....................................................................   1       0    seq
    ...................cgguguccucgacgguccaugugA....................................................................   2       1    seq
    ...................cgguguccucgacgguccaugugu....................................................................   6       0    seq
    ...................cgguguccucgacgguccaugugC....................................................................   1       1    seq
    ....................gguguccucgacgguccaugu......................................................................   2       0    seq
    ....................gguguccucgacgguccaugCg.....................................................................   3       1    seq
    ....................gguguccucgacgguccaugug.....................................................................   26      0    seq
    ....................gguguccucgacgguccaugugA....................................................................   8       1    seq
    ....................gguguccucgacgguccaugugu....................................................................   26      0    seq
    ....................gguguccucgacgguccaugugG....................................................................   2       1    seq
    .........................................................uuggucaguccaggguacaccU................................   1       1    seq
    .........................................................uuggucaguccaggguacacca................................   1       0    seq
    .........................................................uuggucaguccaggguacaccaUu..............................   11      1    seq
```

Provisional ID	:	HPV17_567
Score total	:	22.7
Score for star read(s)	:	3.9
Score for read counts	:	15.9
Score for mfe	:	1.2
Score for randfold	:	1.6
Score for cons. seed	:	
Total read count	:	43
Mature read count	:	25
Loop read count	:	0
Star read count	:	18

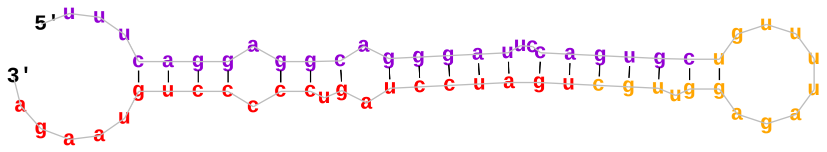

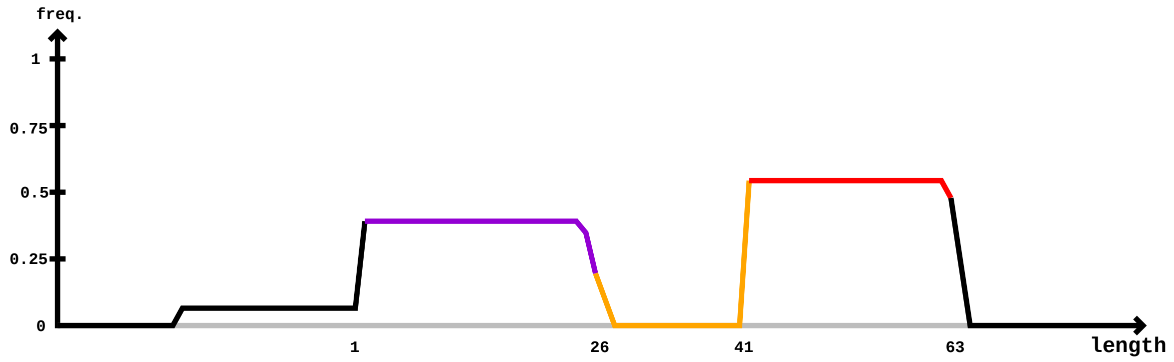


```
                                                      Star                                          Mature
5'- cagauaauuuagauauugacacuccuguuguuucaggaggcagggauuccagugcuguuuuagagguugcugauccuagucccccuguaagaacaagggugucucgaacac   -3'    obs
    cagauaauuuagauauugacacuccuguuguuucaggaggcagggauuccagugcuguuuuagagguugcugauccuagucccccuguaagaacaagggugucucgaacac          exp
    ................(((((((.((((.((.((((.(((.(((((..(((((((........)).)))))))))).).)).)))).)).)))).))))))).........    reads     mm       sample
    ...........gauauugacacuccuguug.................................................................................    2         0        seq
    ...........gauauugacacuccuguuA.................................................................................    1         1        seq
    ..............................uuucaggaggcagggauuccagu..........................................................    1         0        seq
    ..............................uuucaggaggcagggauucccgu..........................................................    1         1        seq
    ..............................uuucaggaggcagggauuccagug.........................................................    7         0        seq
    ..............................uuucaggaggcagggauuccagugU........................................................    6         1        seq
    ..............................uuucaggaggcagggauuccagugA........................................................    2         1        seq
    ..............................uuucaggaggcagggauuccagugG........................................................    1         1        seq
    ......................................................................ugauccuagucccccuguaag....................    3         0        seq
    ......................................................................ugauccuagucccccuguaaga...................    22        0        seq
```

Provisional ID	:	HPV5_237
Score total	:	2.7
Score for star read(s)	:	3.9
Score for read counts	:	-0.8
Score for mfe	:	1.9
Score for randfold	:	-2.2
Score for cons. seed	:	
Total read count	:	10
Mature read count	:	7
Loop read count	:	0
Star read count	:	3

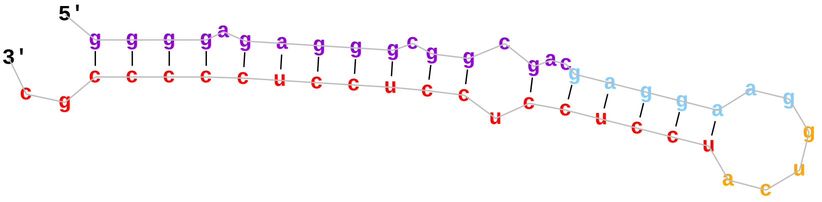

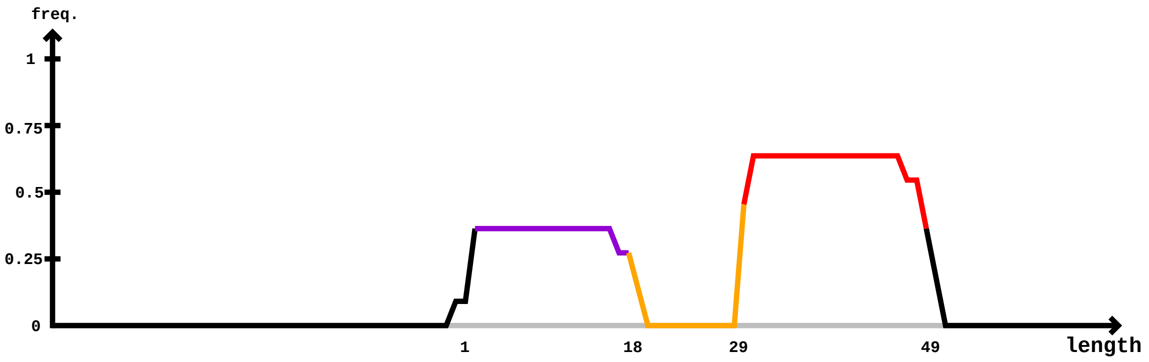

Star Mature

```
5'- aggggcccgagggucgagagggucacgaggagggagccguggggggagagggcggcgacgaggaaggucauccuccuccuccucccccgcccacaaacggucacgaggg  -3'   obs
    aggggcccgagggucgagagggucacgaggagggagccguggggggagagggcggcgacgaggaaggucauccuccuccuccucccccgcccacaaacggucacgaggg         exp
    ...((((....))))....((.((.(......))).))((((((((.(((((.((.(..(((((......)))))).))))))).))).)))))...((....))....   reads   mm   sample
    ........................................gggUggagagggcggcg....................................................   1       1    seq
    ..........................................ggggagagggcggGgac..................................................   3       1    seq
    ......................................................................uccuccuccuccucccc......................   1       0    seq
    ......................................................................uccuccuccuccucccccgU...................   4       1    seq
    .......................................................................ccuccuccuccuccccGg....................   2       1    seq
```

Provisional ID	:	HPV113_1715
Score total	:	2.3
Score for star read(s)	:	-1.3
Score for read counts	:	0
Score for mfe	:	2
Score for randfold	:	1.6
Score for cons. seed	:	
Total read count	:	13
Mature read count	:	13
Loop read count	:	0
Star read count	:	0

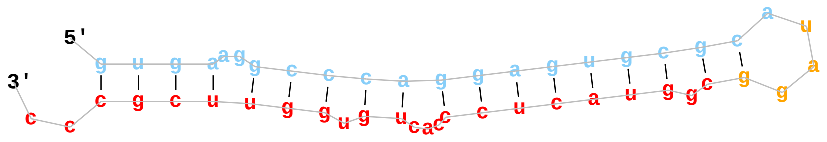

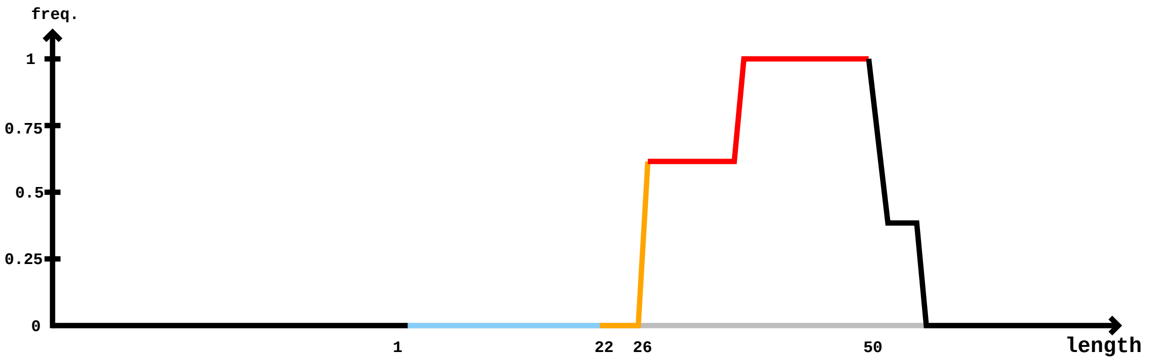

Star Mature

```
5'- cggcacugguggugcuacuggauauguuccuuugggugaaggcccaggagugcgcauaggcgguacucccacugugguucgcccuggggucauaccagagguaaucggc   -3'   exp
    .....(((((..((((.((((.((((..((((.((((((..((((((((((((((....)).)))))))...)).))))))))).)))).)))))))).))))))))).     reads   mm   sample
    ............................................................cgguacucccacugugguucgcAc.........................     8       1    seq
    ......................................................................acugugguucgcccugggg....................     5       0    seq
```

Provisional ID	:	HPV49_1114
Score total	:	2.3
Score for star read(s)	:	-1.3
Score for read counts	:	0
Score for mfe	:	2
Score for randfold	:	1.6
Score for cons. seed	:	
Total read count	:	885
Mature read count	:	885
Loop read count	:	0
Star read count	:	0

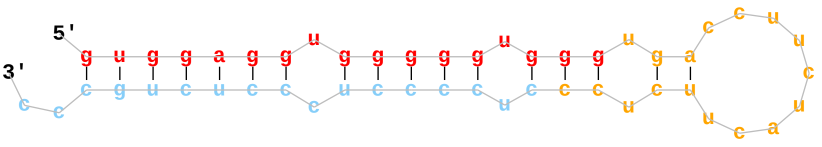

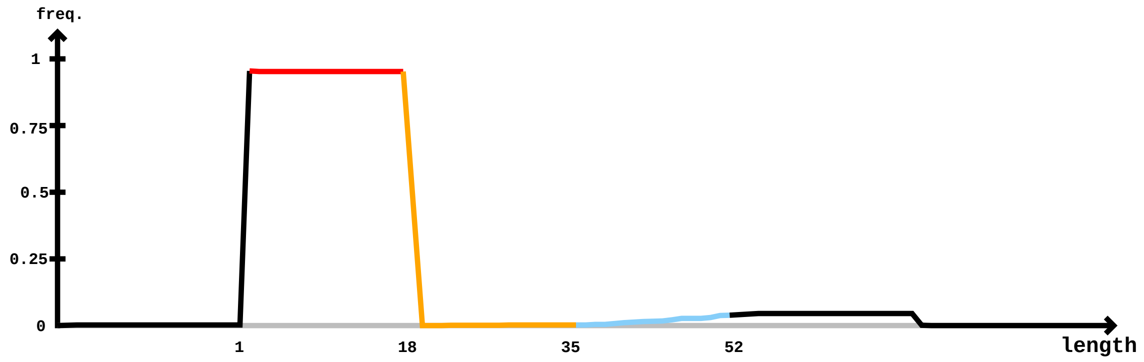

Mature   Star

```
5'- cuucuuucccguuuacugguggagguggggguggguugaccuucuacuucucccuccccucccucugccccugcggguucucuuggggcuggaaucucuggagguugug   -3'    exp
    .......(((((.....((((((((.(((((.(((.((.........)).))).))))).))))))))...))))).(((((.((((......)))).))))).....    reads    mm    sample
    cuucuuucccguuuacugg.........................................................................................    2        0     seq
    ..................guggCgguggggguggg.........................................................................    3        1     seq
    ..................guggUgguggggguggg.........................................................................    882      1     seq
    .......................................cuucuacuucucccuccccucccucugccccugcggguucucuuggggcu...................    1        0     seq
    .............................................cuucucccuccccucccucugccccugcggguucucuuggggc....................    1        0     seq
    ......................................................ccccucccucugccccugcggguucucuuggggc....................    2        0     seq
    ........................................................ccucccucugccccugcggguucucuuggggc....................    3        0     seq
    .........................................................cucccucugccccugcggguucucuuggggc....................    3        0     seq
    ..........................................................ucccucugccccugcggguucucuuggggc....................    2        0     seq
    ...........................................................cccucugccccugcggguucucuuggggc....................    2        0     seq
    ............................................................ccucugccccugcggguucucuuggggc....................    1        0     seq
    .............................................................cucugccccugcggguucucuuggggc....................    1        0     seq
    ..............................................................ucugccccugcggguucucuuggggc....................    4        0     seq
    ...............................................................cugccccugcggguucucuuggggc....................    5        0     seq
    ..................................................................ccccugcggguucucuuggggc....................    3        0     seq
    ...................................................................cccugcggguucucuuggggc....................    7        0     seq
    ....................................................................ccugcggguucucuuggggc....................    1        0     seq
    ......................................................................ugcggguucucuuggggc....................    4        0     seq
    .......................................................................gcggguucucuuggggc....................    2        0     seq
```

Provisional ID	:	HPV3_195
Score total	:	1
Score for star read(s)	:	3.9
Score for read counts	:	-2.3
Score for mfe	:	1.7
Score for randfold	:	-2.2
Score for cons. seed	:	
Total read count	:	7
Mature read count	:	5
Loop read count	:	0
Star read count	:	2

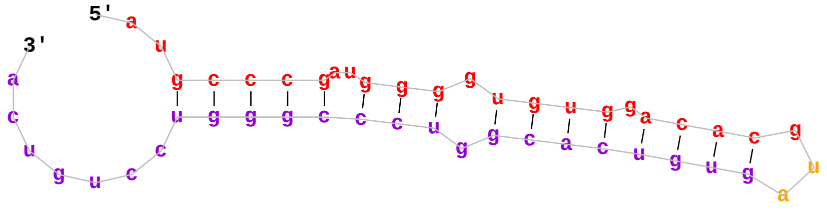

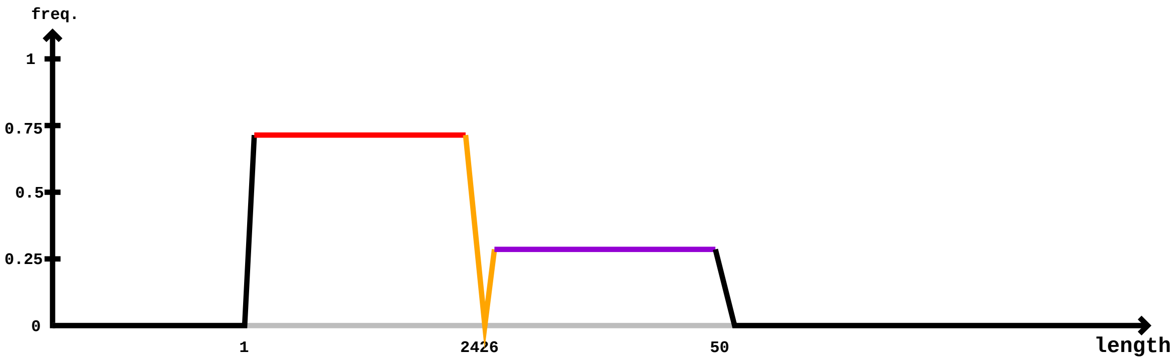

Mature **Star**

```
5'- cacagucagggucacuucgaugcccgauggggugugGacacguagugucacggucccggguccugucaguguccaaugguugacgugcucguucagugguuugcggggcggg   -3'    obs
    cacagucagggucacuucgaugcccgauggggugugGacacguagugucacggucccggguccugucaguguccaaugguugacgugcucguucagugguuugcggggcggg          exp
    (((.(((((((.((((..((((((((..(((.((((.((((...)))))))).))))))))...))))))).)).....))))))))(((((((.((.....)).)))))))   reads   mm    sample
    ...................augcccgauggggugugGacacA......................................................................   5       1     seq
    ............................................gugucacggucccggguccuguca............................................   2       0     seq
```

Provisional ID	:	PePV_1916
Score total	:	0.6
Score for star read(s)	:	-1.3
Score for read counts	:	0
Score for mfe	:	0.3
Score for randfold	:	1.6
Score for cons. seed	:	
Total read count	:	24
Mature read count	:	24
Loop read count	:	0
Star read count	:	0

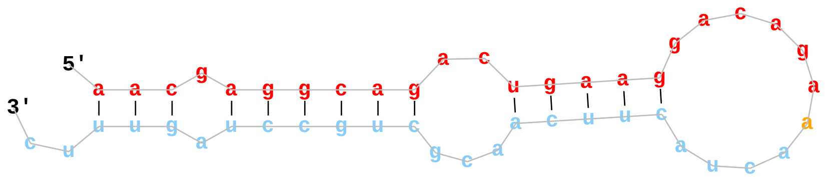

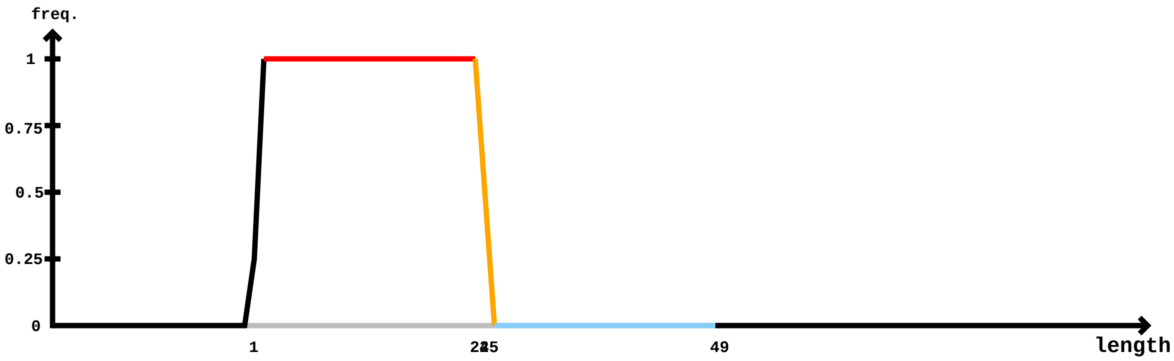

Mature Star

```
5'- ccguaugaaacggcaucagaaacgaggcagacugaaggacagaaacuacuucaacgcugccuaguuucaaaaaauaagacauuaacugcguuagcgguguuuaaagaacuau   -3'    exp
    ((((.....)))).....(((((.((((((..(((((..........)))))...)))))).)))))...............(((((....)))))................     reads       mm          sample
    ..................aaacgaggcagacugaaggacagaa.....................................................................     5           0           seq
    ..................aaacgaggcagacugaaggacagCa.....................................................................     1           1           seq
    ...................aacgaggcagacugaaggacaga......................................................................     10          0           seq
    ...................aacgaggcagacugaaggacagU......................................................................     8           1           seq
```

Provisional ID	:	HPV105_1599
Score total	:	0.6
Score for star read(s)	:	-1.3
Score for read counts	:	-0.3
Score for mfe	:	0.7
Score for randfold	:	1.6
Score for cons. seed	:	
Total read count	:	11
Mature read count	:	10
Loop read count	:	0
Star read count	:	1

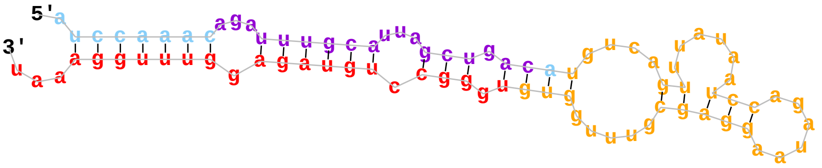

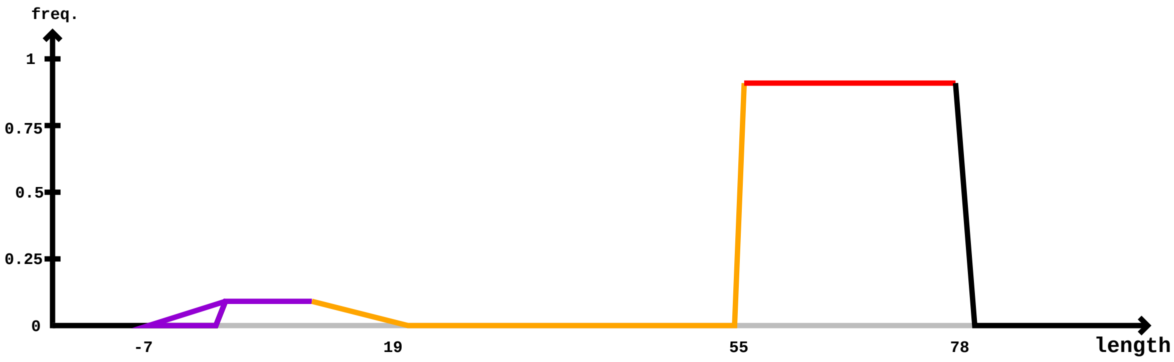

Star **Mature**

```
5'- guugccugauccaaacagauuugcauuagcugacaugucaguuuauaauccagauaaggagcguuuggugugggccuguagagguuuggaaauaaguagagggcaacccuua   -3'   obs
    guugccugauccaaacagauuugcauuagcugacaugucaguuuauaauccagauaaggagcguuuggugugggccuguagagguuuggaaauaaguagagggcaacccuua         exp
    (((((((..(((((((...((((((...(((.((((....((......(((......))))).....)))).))).)))))).)))))))..........)))))))....."   reads   mm   sample
    ................agauuugcauuagcugac..............................................................................   1       0    seq
    ......................................................................ugggccuguagagguuuggaaau...................   9       0    seq
    ......................................................................ugggccuguagagguuuggaaaC...................   1       1    seq
```

Provisional ID	:	FcPV1_22
Score total	:	0
Score for star read(s)	:	-1.3
Score for read counts	:	-2.3
Score for mfe	:	2.1
Score for randfold	:	1.6
Score for cons. seed	:	
Total read count	:	7
Mature read count	:	7
Loop read count	:	0
Star read count	:	0

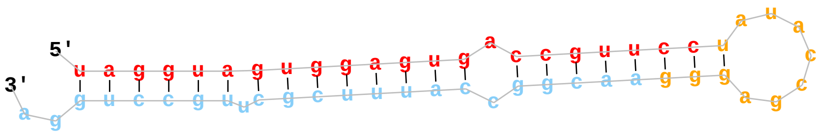

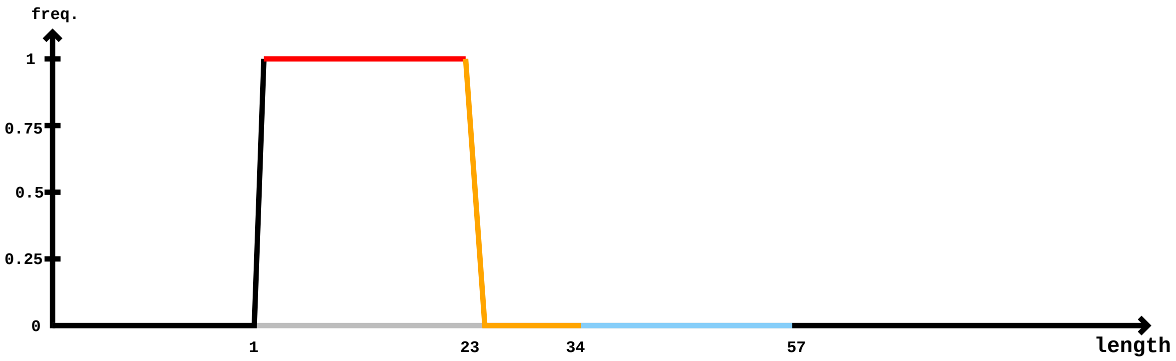

Mature Star

```
5'- agccaauagggguacugguuuagguaguggagugaccguuccuauaccgagggaacggccauuucgcuugccuggaucaagccucccuguaacggucgauguguccgcuaac   -3'    exp
    .....(((((((..((((((((((((((((((((.((((((((.......)))))))).)))))))).))))))))))).))..)))))))..(((.(.....).))).....    reads      mm          sample
    ....................uagguaguggagugaccguucc......................................................................    7          0           seq
```

Provisional ID	:	SV40_2165
Score total	:	79656.8
Score for star read(s)	:	3.9
Score for read counts	:	79650.1
Score for mfe	:	1.2
Score for randfold	:	1.6
Score for cons. seed	:	
Total read count	:	156242
Mature read count	:	154813
Loop read count	:	0
Star read count	:	1429

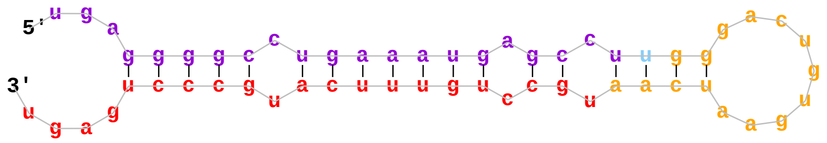

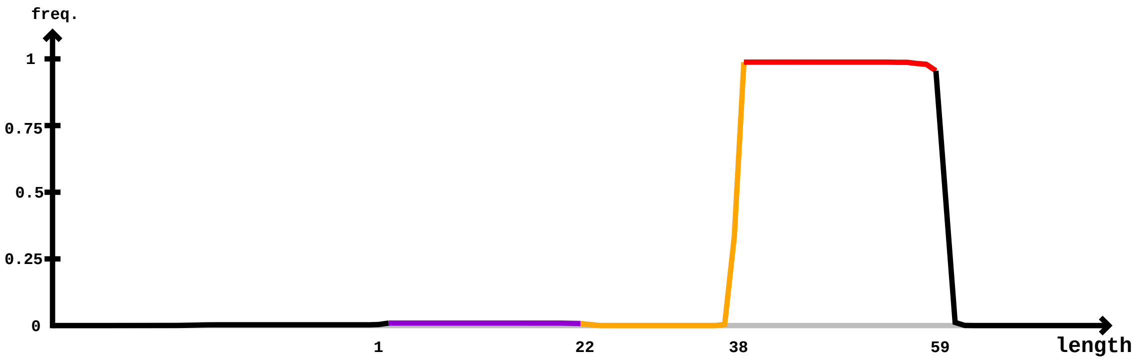

Star **Mature**

```
5'- ggcugauuaugaucaugaacagacugugaggacugaggggccugaaaugagccuugggacugugaaucaaugccuguuucaugcccugagucuuccauguucuucucc  -3'   obs
    ggcugauuaugaucaugaacagacugugaggacugaggggccugaaaugagccuugggacugugaaucaaugccuguuucaugcccugagucuuccauguucuucucc         exp
    ...((((....)))).(((((...((.(((((((..(((((.(((((((.((.((((.........)))).)).))))))).))))).))))))))))))))......    reads     mm     sample
    ...ugauuaugaucaugaacagacugugaggac...........................................................................    6         0      seq
    ...ugauuaugaucaugaacagacugugaggaU...........................................................................    1         1      seq
    .....auuaugaucaugaaUagacu...................................................................................    1         1      seq
    .....auuaugaucaugaacagacugugaggac...........................................................................    4         0      seq
    ......uuaugaucaugaacagacugugagga............................................................................    1         0      seq
    ......uuaugaucaugaacagacugugaggac...........................................................................    3         0      seq
    .......uaugaucaugaacagacugugaggac...........................................................................    8         0      seq
    .......uaugaucaugaacagacugugaggaU...........................................................................    1         1      seq
    ........augaucaugaacagacug..................................................................................    4         0      seq
    ........augaucaugaacagacugugaggaU...........................................................................    1         1      seq
    ........augaucaugaacagacugugaggac...........................................................................    5         0      seq
    .........ugaucaugaacagacugugaggaU...........................................................................    4         1      seq
    .........ugaucaugaacagacugugaggac...........................................................................    3         0      seq
    ..........gaucaugaacagacugugaggac...........................................................................    6         0      seq
    ..........gaucaugaacagacugugaggaU...........................................................................    1         1      seq
    ..........gaucaugaacagacugugaggacugaggggccugaaa.............................................................    1         0      seq
    ...........aucaugaacagacugug................................................................................    3         0      seq
    ...........aucaugaacagacugugagg.............................................................................    1         0      seq
    ...........aucaugaacagacugugaggac...........................................................................    7         0      seq
    ............ucaugaacagacugugaggGc...........................................................................    2         1      seq
    ............ucaugaacagacugugaggac...........................................................................    98        0      seq
    ............ucaugaacagacugugaggaU...........................................................................    1         1      seq
    .............caugaacagacugugaggac...........................................................................    119       0      seq
    .............caugaacagacugugaggaU...........................................................................    2         1      seq
    .............caugaacagacugugaggacu..........................................................................    3         0      seq
    ..............augaacagacugugaggaA...........................................................................    2         1      seq
    ..............augaacagacugugaggac...........................................................................    108       0      seq
    ..............augaacagacugugaggacu..........................................................................    5         0      seq
    ...............ugaacagacugugagga............................................................................    2         0      seq
    ...............ugaacagacugugaggac...........................................................................    20        0      seq
    ...............ugaacagacugugaggacC..........................................................................    1         1      seq
    ...............ugaacagacugugaggacu..........................................................................    5         0      seq
    ...............ugaacagacugugaggacugaggggccugaaa.............................................................    5         0      seq
```

Star **Mature**

```
ggcugauuaugaucaugaacagacugugaggacugaggggccugaaaugagccuugggacugugaaucaaugccuguuucaugcccugagucuuccauguucuucucc
...............ugaacagacugugaggacugaggggccugaaaA............................................................ 2 1 seq
................gaacagacugugaggac........................................................................... 3 0 seq
.................aacagacugugaggacug......................................................................... 1 0 seq
.................aacagacugugaggacugaggggccugaaa............................................................. 3 0 seq
..................acagacugugaggacugaggggccugaaaA............................................................ 1 1 seq
.......................cugugaggacugaggggccugaaaugagccuugggacugugaaucaaugc................................... 1 0 seq
............................aggacugaggggccugaaaugagccuugggacugugaa.......................................... 1 0 seq
................................cugaggggccugaaaug........................................................... 1 0 seq
................................cugaggggccugaaaugag......................................................... 4 0 seq
................................cugaggggccugaaaugagc........................................................ 10 0 seq
................................cugaggggccugaaaugagcc....................................................... 5 0 seq
................................cugaggggccugaaaugagccG...................................................... 2 1 seq
................................cugaggggccugaaaugagAcu...................................................... 1 1 seq
................................cugaggggccugaaaugagccu...................................................... 159 0 seq
................................cugaggggccugaaaugagccA...................................................... 9 1 seq
................................cugaggggccugaaaugagccuA..................................................... 2 1 seq
................................cugaggggccugaaaugagccuG..................................................... 1 1 seq
................................cugaggggccugaaaugagccuu..................................................... 5 0 seq
.................................ugaggggccugaaaugag......................................................... 4 0 seq
.................................ugaggggccugaaaugagU........................................................ 3 1 seq
.................................ugaggggccugaaaugagc........................................................ 106 0 seq
.................................ugaggggccugaaaugagA........................................................ 3 1 seq
.................................ugaggggccugaaaugagcc....................................................... 109 0 seq
.................................ugaggggccugaaaugagcU....................................................... 1 1 seq
.................................ugaggggccugaaaugagccA...................................................... 4 1 seq
.................................ugaggggccugaaaugagccC...................................................... 1 1 seq
.................................ugaggggccugaaaugagUcu...................................................... 1 1 seq
.................................ugaggggccugaaaugagcUu...................................................... 1 1 seq
.................................ugaggggccugaaaugagcGu...................................................... 3 1 seq
.................................ugaggggccugaaaugagcAu...................................................... 1 1 seq
.................................ugaggggccugaaaugagccG...................................................... 2 1 seq
.................................ugaggggccugaaaugagccu...................................................... 853 0 seq
.................................ugaggggccugaaaugagccuu..................................................... 116 0 seq
.................................ugaggggccugaaaugagccuA..................................................... 2 1 seq
.................................ugaggggccugaaaugagccuC..................................................... 3 1 seq
.................................ugaggggccugaaaugagUcuu..................................................... 3 1 seq
.................................ugaggggccugaaaugagccAu..................................................... 1 1 seq
.................................ugaggggccugaaaugagccuuA.................................................... 3 1 seq
.................................ugaggggccugaaaugagccuuU.................................................... 1 1 seq
.................................ugaggggccugaaaugagccuug.................................................... 6 0 seq
.................................ugaggggccugaaaugagccuugC................................................... 1 1 seq
.................................ugaggggccugaaaugagccuuAg................................................... 1 1 seq
.................................ugaggggccugaaaugagccuugA................................................... 1 1 seq
.................................ugaggggccugaaaugagccuugggacugug............................................ 2 0 seq
.................................ugaggggccugaaaugagcUuugggacugug............................................ 1 1 seq
.................................ugaggggccugaaaugagccuugAgacugugaaucaa...................................... 1 1 seq
.................................ugaggggccugaaaugagccuugggacugugaaucaaugcc.................................. 2 0 seq
.................................ugaggggccugaaaugagccuugggacugugaaucaaugccuguuucaug......................... 1 0 seq
.............................................aaugagccuugggacug.............................................. 2 0 seq
.............................................aaugagccuugggacugugaauc........................................ 1 0 seq
...............................................ugagccuugggacugugaauc........................................ 1 0 seq
...............................................ugagccuugggacugugaauca....................................... 1 0 seq
...............................................ugagccuugggacugugaaucaaug.................................... 2 0 seq
...............................................ugagccuugggacugugaaucaaugccuguu.............................. 1 0 seq
...............................................ugagccuugggacugugaaucaaugccuguuu............................. 1 0 seq
...........................................................cugugaaucaaugccug................................ 3 0 seq
.............................................................gugaaucaaugccuguuucaugcccugagu................. 1 0 seq
...................................................................caaugccuguuucaugcccugagu................. 1 0 seq
....................................................................aaugccuguuucaugcc....................... 1 0 seq
....................................................................aaugccuguuucaugcccug.................... 4 0 seq
....................................................................aaugccuguuucaugcccuga................... 15 0 seq
....................................................................aaugccuguuucaugcccugaA.................. 3 1 seq
....................................................................aaugccuguuucaugcccugCg.................. 1 1 seq
....................................................................aaugccuguuucaugcccugag.................. 143 0 seq
....................................................................aaugccuguuucaugcccugaU.................. 19 1 seq
....................................................................aaugccuguuucaugcccugagA................. 87 1 seq
....................................................................aaugccuguuucaugcccugagG................. 1 1 seq
....................................................................aaugccuguuucaugcccugagC................. 7 1 seq
....................................................................aaugccuguuucaugcccugagu................. 361 0 seq
....................................................................aaugccuguuucaugcccugagAc................ 1 1 seq
```

Star **Mature**

```
ggcugauuaugaucaugaacagacugugaggacugaggggccugaaaugagccuugggacugugaaucaaugccuguuucaugcccugagucuuccauguucuucucc
....................................................................aaugccuguuucaugcccugaguAu...............	4	1	seq
....................................................................aaugccuguuucaugcccugaguUu...............	1	1	seq
....................................................................aaugccuguuucaugcccugaguUuu..............	1	1	seq
.....................................................................augccuguuucaugccc......................	114	0	seq
.....................................................................augccuguuucaugcccuU....................	4	1	seq
.....................................................................augccuguuucaugcccuA....................	3	1	seq
.....................................................................augccuguuucaugcccug....................	210	0	seq
.....................................................................augccuguuucaugcccuga...................	239	0	seq
.....................................................................augccuguuucaugcccugU...................	2	1	seq
.....................................................................augccuguuucaugcccuAa...................	1	1	seq
.....................................................................augccuguuucaugcccugG...................	1	1	seq
.....................................................................augccuguuucaugcccugUg..................	1	1	seq
.....................................................................augccuguuucaugcccugaU..................	13	1	seq
.....................................................................augccuguuucaugcccAgag..................	1	1	seq
.....................................................................augccuguuucaugcccugGg..................	7	1	seq
.....................................................................augccuguuucaugcccugaA..................	38	1	seq
.....................................................................augccuguuucaugcccugag..................	1502	0	seq
.....................................................................augccuguuucaugcccugagA.................	1051	1	seq
.....................................................................augccuguuucaugcccuCagu.................	2	1	seq
.....................................................................augccuguuucaugcccugUgu.................	12	1	seq
.....................................................................augccuguuucaugcccugagu.................	44935	0	seq
.....................................................................augccuguuucaugcccugGgu.................	92	1	seq
.....................................................................augccuguuucaugcccugagC.................	730	1	seq
.....................................................................augccuguuucaugcccugaCu.................	10	1	seq
.....................................................................augccuguuucaugcccugaUu.................	12	1	seq
.....................................................................augccuguuucaugcccugNgu.................	1	1	seq
.....................................................................augccuguuucaugcccuAagu.................	8	1	seq
.....................................................................augccuguuucaugcccugCgu.................	7	1	seq
.....................................................................augccuguuucaugcccugaAu.................	15	1	seq
.....................................................................augccuguuucaugcccAgagu.................	22	1	seq
.....................................................................augccuguuucaugcccugagG.................	140	1	seq
.....................................................................augccuguuucaugcccuUagu.................	2	1	seq
.....................................................................augccuguuucaugcccuNagu.................	4	1	seq
.....................................................................augccuguuucaugcccugaguc................	136	0	seq
.....................................................................augccuguuucaugcccugaguG................	28	1	seq
.....................................................................augccuguuucaugcccugagAc................	1	1	seq
.....................................................................augccuguuucaugcccugaguA................	959	1	seq
.....................................................................augccuguuucaugcccugaguU................	598	1	seq
.....................................................................augccuguuucaugcccugagucA...............	19	1	seq
.....................................................................augccuguuucaugcccugagucC...............	5	1	seq
.....................................................................augccuguuucaugcccugaguAu...............	161	1	seq
.....................................................................augccuguuucaugcccugaguGu...............	26	1	seq
.....................................................................augccuguuucaugcccugaguUu...............	282	1	seq
.....................................................................augccuguuucaugcccugagucu...............	74	0	seq
.....................................................................augccuguuucaugcccugagucAu..............	3	1	seq
.....................................................................augccuguuucaugcccugaguUuu..............	18	1	seq
.....................................................................augccuguuucaugcccugaguAuu..............	9	1	seq
.....................................................................augccuguuucaugcccugaguUuuc.............	1	1	seq
......................................................................ugccuguuucaugcccu.....................	3	0	seq
......................................................................ugccuguuucaugcccug....................	453	0	seq
......................................................................Agccuguuucaugcccug....................	2	1	seq
......................................................................ugccuguuucaugcccuga...................	208	0	seq
......................................................................ugccuguuucaugcccugG...................	3	1	seq
......................................................................Agccuguuucaugcccuga...................	1	1	seq
......................................................................ugccuguuucaugcccugU...................	1	1	seq
......................................................................ugccuguuucaugcccugaN..................	1	1	seq
......................................................................ugccuguuucaugcccugGg..................	5	1	seq
......................................................................ugccuguuucaugcccugag..................	2029	0	seq
......................................................................ugccuguuucaugcccugaA..................	70	1	seq
......................................................................ugccuguuucaugcccugaC..................	1	1	seq
......................................................................ugccuguuucaugcccugaU..................	11	1	seq
......................................................................ugccuguuucaugcccugagG.................	293	1	seq
......................................................................ugccuguuucaugcccugCgu.................	8	1	seq
......................................................................ugccuguuucaugcccugagu.................	91503	0	seq
......................................................................ugccuguuucaugcccugNgu.................	2	1	seq
......................................................................ugccuguuucaugcccugaCu.................	30	1	seq
......................................................................ugccuguuucaugcccugaUu.................	21	1	seq
......................................................................ugccuguuucaugcccAgagu.................	27	1	seq
......................................................................ugccuguuucaugcccugagC.................	1978	1	seq
......................................................................ugccuguuucaugcccugaNu.................	1	1	seq
```

Star **Mature**

```
ggcugauuaugaucaugaacagacugugaggacugaggggccugaaaugagccuugggacugugaaucaaugccuguuucaugcccugagucuuccauguucuucucc
......................................................................ugccuguuucaugcccugagA.................   1478   1   seq
......................................................................ugccuguuucaugcccugaAu.................   41     1   seq
......................................................................ugccuguuucaugcccugGgu.................   132    1   seq
......................................................................Agccuguuucaugcccugagu.................   6      1   seq
......................................................................ugccuguuucaugcccugUgu.................   27     1   seq
......................................................................ugccuguuucaugcccugagAc................   7      1   seq
......................................................................ugccuguuucaugcccugGguc................   2      1   seq
......................................................................ugccuguuucaugcccugaguc................   1173   0   seq
......................................................................ugccuguuucaugcccugaguG................   75     1   seq
......................................................................ugccuguuucaugcccugaguA................   917    1   seq
......................................................................ugccuguuucaugcccugCguc................   1      1   seq
......................................................................ugccuguuucaugcccugagCc................   1      1   seq
......................................................................ugccuguuucaugcccAgaguc................   1      1   seq
......................................................................ugccuguuucaugcccugaguU................   893    1   seq
......................................................................ugccuguuucaugcccugagGc................   2      1   seq
......................................................................ugccuguuucaugcccugagucC...............   3      1   seq
......................................................................ugccuguuucaugcccugaguUu...............   275    1   seq
......................................................................ugccuguuucaugcccugaguAu...............   437    1   seq
......................................................................ugccuguuucaugcccugaguGu...............   43     1   seq
......................................................................ugccuguuucaugcccugagucu...............   215    0   seq
......................................................................ugccuguuucaugcccugagucA...............   184    1   seq
......................................................................ugccuguuucaugcccugagucG...............   10     1   seq
......................................................................ugccuguuucaugcccugagucuA..............   3      1   seq
......................................................................ugccuguuucaugcccugagucAu..............   9      1   seq
......................................................................ugccuguuucaugcccugaguAuu..............   13     1   seq
......................................................................ugccuguuucaugcccugaguUuu..............   18     1   seq
......................................................................ugccuguuucaugcccugagucuu..............   4      0   seq
......................................................................ugccuguuucaugcccugaguGuu..............   2      1   seq
......................................................................ugccuguuucaugcccugagucuC..............   1      1   seq
......................................................................ugccuguuucaugcccugagucGu..............   2      1   seq
.......................................................................gccuguuucaugcccugagu.................   21     0   seq
.......................................................................gccuguuucaugcccugaguU................   2      1   seq
.......................................................................gccuguuucaugcccugaguc................   8      0   seq
........................................................................ccuguuucaugcccugagu.................   2      0   seq
........................................................................ccuguuucaugcccugaguA................   2      1   seq
.........................................................................cuguuucaugcccugagu.................   1      0   seq
..........................................................................uguuucaugcccugagu.................   3      0   seq
```

Provisional ID	:	MCPyV_2088
Score total	:	7811.6
Score for star read(s)	:	3.9
Score for read counts	:	7804.5
Score for mfe	:	1.6
Score for randfold	:	1.6
Score for cons. seed	:	
Total read count	:	15320
Mature read count	:	11338
Loop read count	:	0
Star read count	:	3982

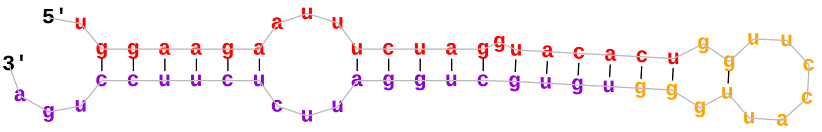

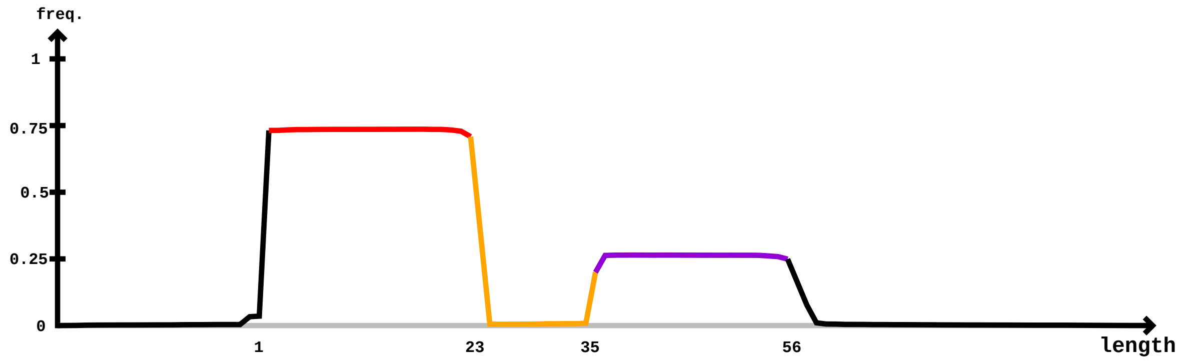

Mature **Star**

5'-	cucccaggugccauacguucuggaagaauuucuagguacacugguuccauugggugugcuggauucucuuccugaauugguggucuccucucugcuacuggauccagaggau	-3'	obs	
	cucccaggugccauacguucuggaagaauuucuagguacacugguuccauugggugugcuggauucucuuccugaauugguggucuccucucugcuacuggauccagaggau		exp	
	.....(((.(((((..((((.((((((...(((((.((((((.(......).)))))))))))...)))))).))))..)))))..)))(((((..........)))))...	reads	mm	sample
	cucccaggugccauacguuc..	8	0	seq
	cucccaggugccauacguucuggaagaauuucuagguacacugguuccauu...	1	0	seq
	.ucccaggugccauacguuc..	15	0	seq
	..cccaggugccauacguucuggaagaauuucuagguacacugguuccauugg...	4	0	seq
	...ccaggugccauacguucuggaagaauuucuagguacacugguuccauuggg..	3	0	seq
	....caggugccauacguucuggaagaauuucuagguacacugguuccauugggu...	1	0	seq
	.....aggugccauacguucuggaagaauuucuagguacacugguuccauugggug..	2	0	seq
	.......gugccauacguucuggaagaauuucuagguacacugguuccauugggugug..	1	0	seq
	........ugccauacguucuggaagaauuucuagguacacugguuccauugggugugc...	3	0	seq
	.........gccauacguucuggaagaauuucuagguacacugguuccauugggugugcu..	3	0	seq
	...........cauacguucuggaagaauuucuagguacac...	8	0	seq
	...........cauacguucuggaagaauuucuagguacacugguuccauugggugugcugg..	1	0	seq
	............auacguucuggaagaauuucuagguacacugguuccauugggugugcugga...	2	0	seq
	.............uacguucuggaagaauuucuagguacacugguuccauugggugugcuggau..	1	0	seq
	..............acguucuggaagaauuucuagg..	1	0	seq
	..............acguucuggaagaauuucuagguacac...	1	0	seq
	..............acguucuggaagaauuucuagguacacugguuccauugggugugcuggauu...	3	0	seq
	...............cguucuggaagaauuucuagguacac...	2	0	seq
	................guucuggaagaauuucuagguacacugguuccauugggugugcuggauucu...	1	0	seq
	..................ucuggaagaauuucuagguac...	6	0	seq
	..................ucuggaagaauuucuagguaca..	9	0	seq
	..................ucuggaagaauuucuagguacac...	101	0	seq
	..................ucuggaagaauuucuagguacaA...	1	1	seq
	..................ucuggaagaauuucuagguacaU...	1	1	seq
	..................ucuggaagaauuucuagguacacA..	10	1	seq
	..................ucuggaagaauuucuagguGcacu..	2	1	seq
	..................ucuggaagaauuucuagguacacC..	5	1	seq
	..................ucuggaagaauuucuagguacacG..	1	1	seq
	..................ucuggaagaauuucuagguacacu..	307	0	seq
	..................ucuggaagaauuucuagguaUacu..	1	1	seq
	..................ucuggaagaauuucuaggNacacu..	1	1	seq
	..................ucuggaagaauuucuagguacacAg...	2	1	seq
	..................ucuggaagaauuucuagguacacugguu..	2	0	seq

Mature   Star

```
cucccaggugccauacguucuggaagaauuucuagguacacugguuccauugggugugcuggauucucuuccugaauugguggucuccucucugcuacuggauccagaggau
..................ucuggaagaauuucuagguacacugguC..................................................................  1     1   seq
..................ucuggaagaauuucuagguacacugguuccauugggu.........................................................  3     0   seq
..................ucuggaagaauuucuagguacacugguuccauugggugugcuggauucucu...........................................  1     0   seq
...................cuggaagaauuucuagguacacu......................................................................  34    0   seq
....................uggaagaauuucuaggu...........................................................................  12    0   seq
....................uggaagaauuucuaggua..........................................................................  5     0   seq
....................uggaagaauuucuagguaU.........................................................................  1     1   seq
....................uggaagaauuucuagguac.........................................................................  30    0   seq
....................uggaagaauuucuagguaca........................................................................  62    0   seq
....................uggaagaauuucuagguacaU.......................................................................  9     1   seq
....................uggaagaauuucuagguacac.......................................................................  190   0   seq
....................uggaagaauuucuagguacacu......................................................................  9513  0   seq
....................uggaagaauuucuagguaUacu......................................................................  8     1   seq
....................uggaagaauuucuagguacGcu......................................................................  37    1   seq
....................uggaagaauuucuagguacUcu......................................................................  2     1   seq
....................uggaagaauuucuagguacacA......................................................................  173   1   seq
....................uggaagaauuucuagguacacC......................................................................  549   1   seq
....................uggaagaauuucuagguacacG......................................................................  54    1   seq
....................uggaagaauuucuagguacCcu......................................................................  1     1   seq
....................uggaagaauuucuagguacaGu......................................................................  1     1   seq
....................uggaagaauuucuagguacaUu......................................................................  6     1   seq
....................uggaagaauuucuagguacacuC.....................................................................  5     1   seq
....................uggaagaauuucuagguacacug.....................................................................  15    0   seq
....................uggaagaauuucuagguacacuU.....................................................................  63    1   seq
....................uggaagaauuucuagguacacuA.....................................................................  63    1   seq
....................uggaagaauuucuagguacacugU....................................................................  1     1   seq
....................uggaagaauuucuagguacacuUg....................................................................  2     1   seq
....................uggaagaauuucuagguacacuAg....................................................................  1     1   seq
....................uggaagaauuucuagguacGcugguuc.................................................................  1     1   seq
....................uggaagaauuucuagguacacugguuc.................................................................  2     0   seq
....................uggaagaauuucuagguacacugguuU.................................................................  1     1   seq
....................uggaagaauuucuagguacacugguuccauugggugugcuggauucucuuc.........................................  1     0   seq
.....................ggaagaauuucuagguacacu......................................................................  3     0   seq
.....................ggaagaauuucuagguacacugguuccauugggugugcuggauucucuucc........................................  1     0   seq
......................gaagaauuucuagguacac.......................................................................  2     0   seq
......................gaagaauuucuagguacacC......................................................................  1     1   seq
......................gaagaauuucuagguacacu......................................................................  27    0   seq
.......................aagaauuucuagguacacG......................................................................  1     1   seq
.......................aagaauuucuagguacacu......................................................................  12    0   seq
.......................aagaauuucuagguacacugguuccauugggugugcuggauucucuuccug......................................  3     0   seq
........................agaauuucuagguacacu......................................................................  1     0   seq
........................agaauuucuagguacacugguuccauugggugugcuggauucucuuccuga.....................................  1     0   seq
.........................gaauuucuagguacacugguuccauugggugugcuggauucucuuccugaa....................................  4     0   seq
.........................gaauuucuagguacacugguuccauugggugugcuggauAcucuuccugaa....................................  1     1   seq
.........................gaauuucuagguacacugguuccauugggugugcuggauuGucuuccugaa....................................  1     1   seq
..........................aauuucuagguacacugguuccauugggugugcuggauucucuuccugaau...................................  3     0   seq
...........................auuucuagguacacugguuccauugggugugcuggauucucuuccugaauu..................................  1     0   seq
............................uuucuagguacacugguuccauugggugugcuggauucucuuccugaauug.................................  1     0   seq
..............................ucuagguacacugguuccauugggugugcuggauucucuuccugaauuggu...............................  1     0   seq
...............................cuagguacacugguuccauugggugugcuggauucucuuccugaauuggug..............................  1     0   seq
................................uagguacacugguuccauugggugugcuggauucucuuccugaauuggugg.............................  2     0   seq
..................................gguacacugguuccauugggugugcuggauucucuuccugaauuggugguc...........................  3     0   seq
...................................guacacugguuccauugggugugcuggauucucuuccugaauugguggucu..........................  1     0   seq
....................................uacacugguuccauugggugugcuggauucucuuccugaauugguggucuc.........................  2     0   seq
.....................................acacugguuccauugggugugcuggauucucuuccugaauugguggucucc........................  1     0   seq
......................................cacugguuccauugggugugcuggauucucuuccugaauugg................................  1     0   seq
......................................cacugguuccauugggugugcuggauucucuuccugaauugguggucuccu.......................  3     0   seq
.......................................acugguuccauugggugugcuggauucucuuccugaauugguggucuccuc......................  2     0   seq
........................................cugguuccauugggugugcuggauucucuuccugaauugguggucuccucu.....................  2     0   seq
.........................................ugguuccauugggugugcuggauucucuuccugaauugguggucuccucuc....................  1     0   seq
..........................................gguuccauugggugugcuggauucucuuccugaauugguggucuccucucu...................  2     0   seq
.............................................uccauugggugugcuggauucucuuccugaauugguggucuccucucugcu................  1     0   seq
..............................................ccauugggugugcuggauucucuuccugaauugg................................  1     0   seq
..............................................ccauugggugugcuggauucucuuccugaauuggu...............................  1     0   seq
..............................................ccauugggugugcuggauucucuuccugaauugguggucuccucucugcua...............  2     0   seq
...............................................cauugggugugcuggauucucuuccugaauugg................................  3     0   seq
...............................................cauugggugugcuggauucucuuccugaauugA................................  1     1   seq
...............................................cauugggugugcuggauucucuuccugaauuggugg.............................  2     0   seq
...............................................cauugggugugcuggauucucuuccugaauugguggucuccucucugcuac..............  2     0   seq
................................................auugggugugcuggauucucuuccugaauugg................................  1     0   seq
```

```
                    Mature                              Star
cucccaggugccauacguucuggaagaauuucuagguacacuggguuccauuggguugugcuggauucucuuccugaauugguggucuccucucugcuacuggauccagaggau
................................................auugggugugcuggauucucuuccugaauuggugg.............................	1	0	seq
................................................auugggugugcuggauucucuuccugaauugguggucucc........................	1	0	seq
................................................auugggugugcuggauucucuuccugaauugguggucuccucucugcuacu.............	3	0	seq
.................................................uugggugugcuggauucucuuc.........................................	1	0	seq
.................................................uugggugugcuggauucucuuccAg......................................	1	1	seq
.................................................uugggugugcuggauucucuuccugUau...................................	1	1	seq
.................................................uugggugugcuggauucucuuccugaau...................................	1	0	seq
.................................................uugggugugcuggauucucuuccugaauugg................................	5	0	seq
.................................................uugggugugcuggauucucuuccugaauugU................................	1	1	seq
.................................................uugggugugcuggauucucuuccugaauuggugg.............................	3	0	seq
.................................................uugggugugcuggauucucuuccugaauugguggucc...........................	1	0	seq
.................................................uugggugugcuggauucucuuccugaauugguggucuccucuc....................	1	0	seq
.................................................uugggugugcuggauucucuuccugaauugguggucuccucucugcuacug............	1	0	seq
..................................................ugggugugcuggauucucuuccugaauugg................................	1	0	seq
..................................................ugggugugcuggauucucuuccugaauugguggucuccucucugcuacugg...........	1	0	seq
...................................................gggugugcuggauucucuuccugaauugg................................	3	0	seq
....................................................ggugugcuggauucucuuccugaauuggCgguc...........................	1	1	seq
.....................................................gugugcuggauucucuuccuU......................................	1	1	seq
.....................................................gugugcuggauucucuuccug......................................	2	0	seq
.....................................................gugugcuggauucucuuccCga.....................................	1	1	seq
.....................................................gugugcuggauucucuuccuga.....................................	18	0	seq
.....................................................gugugcuggauucucuuccugaU....................................	2	1	seq
.....................................................gugugcuggauucucuuccugaaA...................................	2	1	seq
.....................................................gugugcuggauucucuuccugaauugguggucuccucucugcuacuggauc........	2	0	seq
......................................................ugugcuggauucucuuc.........................................	2	0	seq
......................................................ugugcuggauucucuucc........................................	35	0	seq
......................................................ugugcuggauucucuuccu.......................................	35	0	seq
......................................................ugugcuggauucucuuccC.......................................	4	1	seq
......................................................ugugcuggauucucuuccug......................................	127	0	seq
......................................................ugugcuggauucucuuccuC......................................	1	1	seq
......................................................ugugcuggauucucuuccuA......................................	6	1	seq
......................................................ugugcuggauucucuuccGga.....................................	1	1	seq
......................................................ugugcuggauucucuuccCga.....................................	1	1	seq
......................................................ugugcuggauucucuuccugG.....................................	11	1	seq
......................................................ugugcuggauucucuuccugC.....................................	4	1	seq
......................................................ugugcuggauucucuuccuga.....................................	2249	0	seq
......................................................ugugcuggauucucuuccugU.....................................	36	1	seq
......................................................ugugcuggauucucuuccugaC....................................	5	1	seq
......................................................ugugcuggauucucuuccugaa....................................	119	0	seq
......................................................ugugcuggauucucuuccugUa....................................	4	1	seq
......................................................ugugcuggauucucuuccugaG....................................	20	1	seq
......................................................ugugcuggauucucuuccugaU....................................	130	1	seq
......................................................ugugcuggauucucuuccugaaC...................................	5	1	seq
......................................................ugugcuggauucucuuccugaaG...................................	22	1	seq
......................................................ugugcuggauucucuuccugaaA...................................	65	1	seq
......................................................ugugcuggauucucuuccugaau...................................	63	0	seq
......................................................ugugcuggauucucuuccugaUu...................................	3	1	seq
......................................................ugugcuggauucucuuccugaaGu..................................	2	1	seq
......................................................ugugcuggauucucuuccugaauu..................................	3	0	seq
......................................................ugugcuggauucucuuccugaauC..................................	1	1	seq
......................................................ugugcuggauucucuuccugaaAu..................................	1	1	seq
......................................................ugugcuggauucucuuccugaauA..................................	4	1	seq
......................................................ugugcuggauucucuuccugaauugguggucuccuc......................	1	0	seq
......................................................ugugcuggauucucuuccugaauugguggucuccucucugcuacuggaucc.......	4	0	seq
.......................................................gugcuggauucucuucc........................................	2	0	seq
.......................................................gugcuggauucucuuccu.......................................	2	0	seq
.......................................................gugcuggauucucuuccug......................................	3	0	seq
.......................................................gugcuggauucucuuccuga.....................................	26	0	seq
.......................................................gugcuggauucucuuccugaG....................................	1	1	seq
.......................................................gugcuggauucucuuccugaa....................................	30	0	seq
.......................................................gugcuggauucucuuccugaau...................................	815	0	seq
.......................................................gugcuggauucucuuccugaaC...................................	34	1	seq
.......................................................gugcuggauucucuuccugaUu...................................	2	1	seq
.......................................................gugcuggauucucuuccugaaG...................................	1	1	seq
.......................................................gugcuggauucucuuccugGau...................................	2	1	seq
.......................................................gugcuggauucucuuccugaGu...................................	2	1	seq
.......................................................gugcuggauucucuuccugaaA...................................	5	1	seq
.......................................................gugcuggauucucuuccugaGuu..................................	2	1	seq
.......................................................gugcuggauucucuuccugaauC..................................	3	1	seq
.......................................................gugcuggauucucuuccugaauu..................................	34	0	seq
```

```
                    Mature                           Star
cucccaggugccauacguucuggaagaauuucuagguacacugguuccauugggugugcuggauucucuuccugaauugguggucuccucucugcuacuggauccagaggau
.......................................................gugcuggauucucuuccugaauA..................................    14    1    seq
.......................................................gugcuggauucucuuccugaauuU.................................    4     1    seq
.......................................................gugcuggauucucuuccugaauugguggucuccucucugcuacuggaucca......    2     0    seq
........................................................ugcuggauucucuuccuga.....................................    2     0    seq
........................................................ugcuggauucucuuccugaau...................................    9     0    seq
........................................................ugcuggauucucuuccugaauu..................................    4     0    seq
........................................................ugcuggauucucuuccugaauugguggucuccucucugcuacuggauccag.....    3     0    seq
.........................................................gcuggauucucuuccugaauugguggucuccucucugcuacuggauccaga....    2     0    seq
..........................................................cuggauucucuuccugaauugguggucuccucucugcuacuggauccagag...    3     0    seq
...........................................................uggauucucuuccugaauugguggucuccucucugcuacuggauccagagg..    2     0    seq
.............................................................gauucucuuccugaauugguggucuccucucugcuacuggauccagaggau    2     0    seq
.............................................................gauucucuuccugaauugguggucuccucucugcuacuggCuccagaggau    1     1    seq
....................................................................uuccugaauugguggucuccucucu...................    1     0    seq
........................................................................ugaauugguggucuccuc......................    1     0    seq
.........................................................................gaauugguggucuccuc......................    1     0    seq
..........................................................................aauugguggucuccucucugc.................    1     0    seq
```
