# Supplementary material for: Identification of virus-encoded microRNAs in divergent Papillomaviruses
Source: PLoS Pathog. 2018 Jul 26;14(7):e1007156. doi: 10.1371/journal.ppat.1007156 (PMC6062147; doi:10.1371/journal.ppat.1007156)

PV miRNA Northern

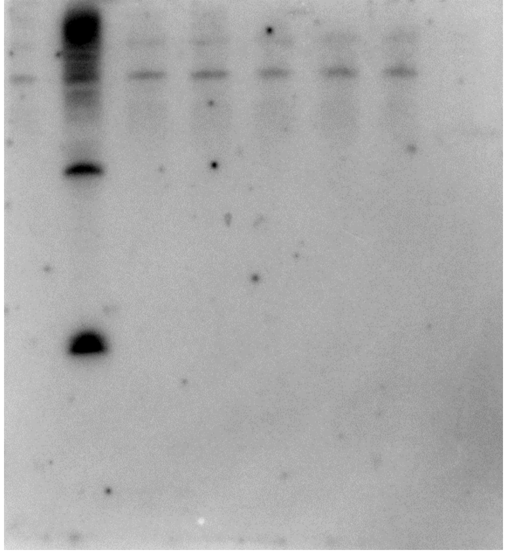

SV40-miR-S1 probe

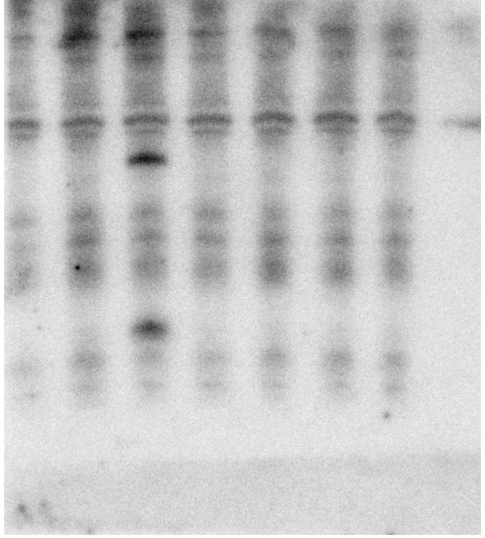

FcPV-miR-F1 3pprobe

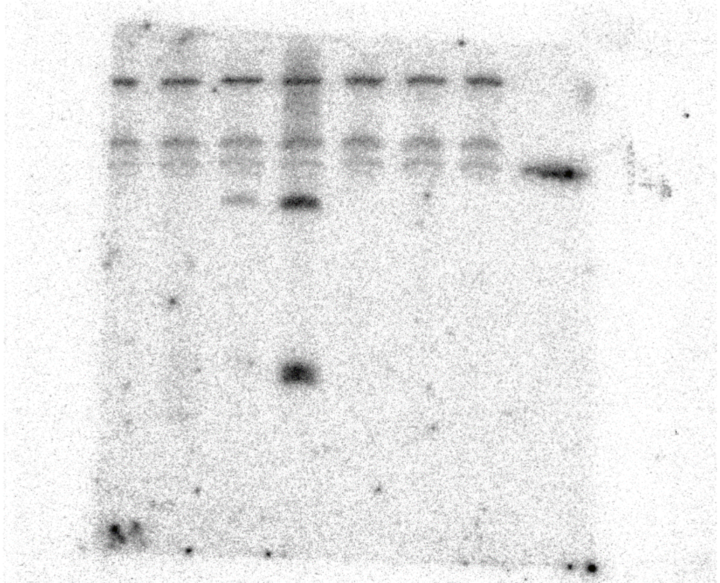

FcPV-miR-F2 3p probe

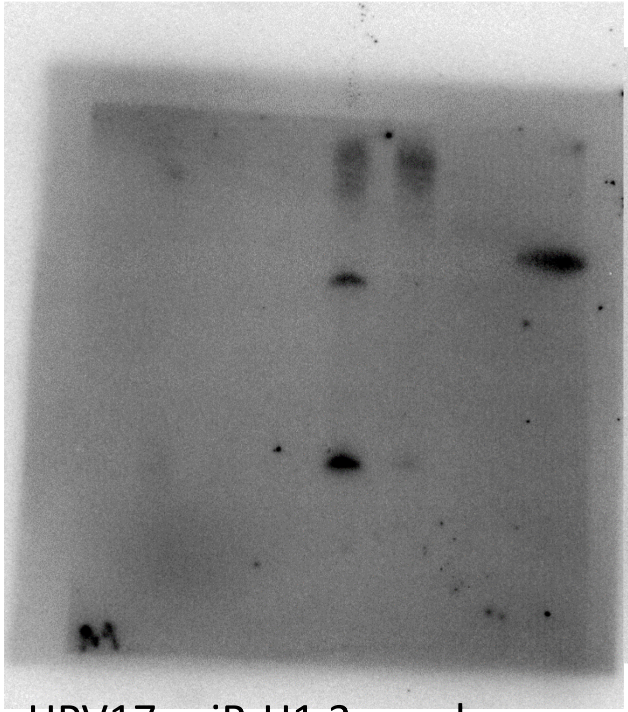

HPV17-miR-H1 3p probe

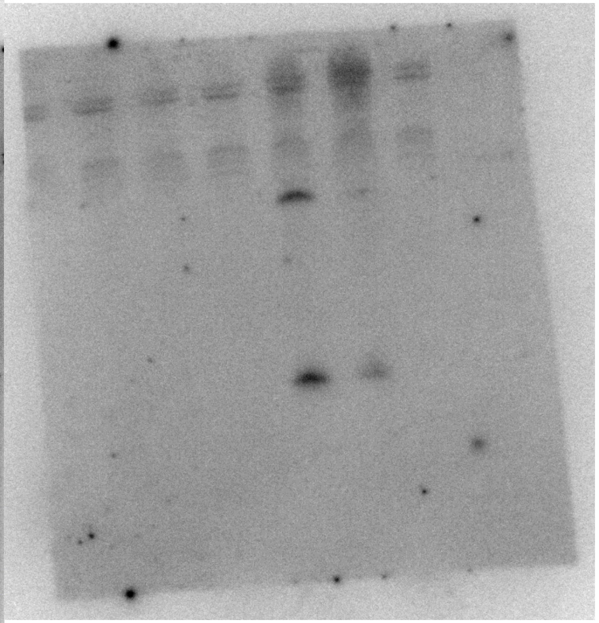

HPV37-miR-H1 3p probe

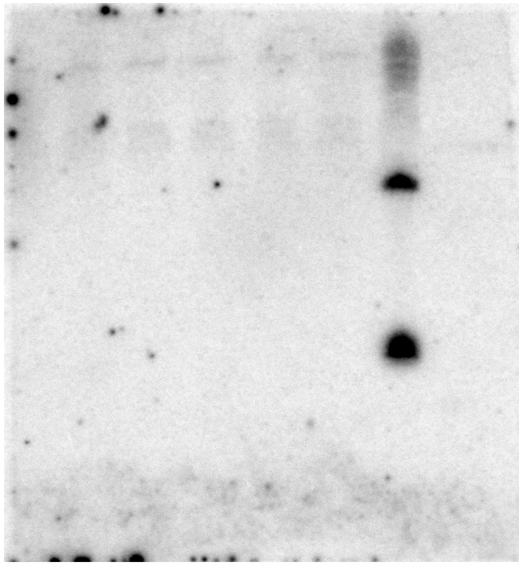

HPV41-miR-H1 5p probe

PV miRNA Drosha Dependence

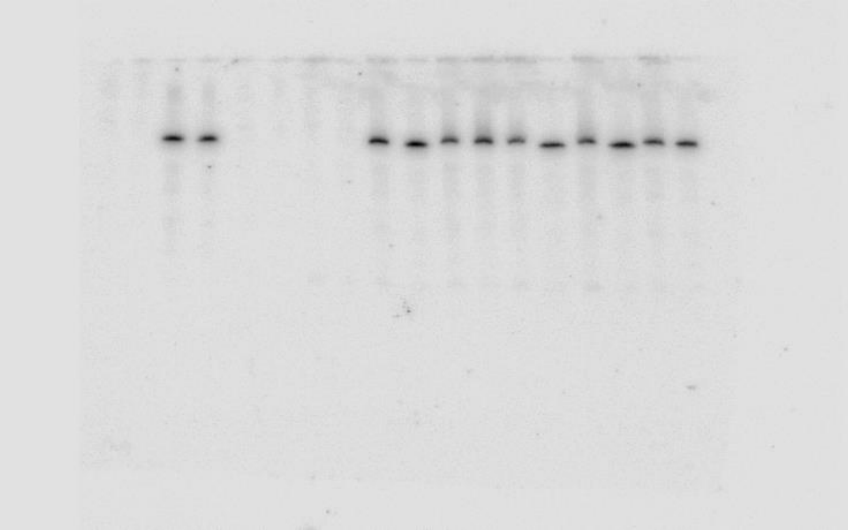

HSUR probe

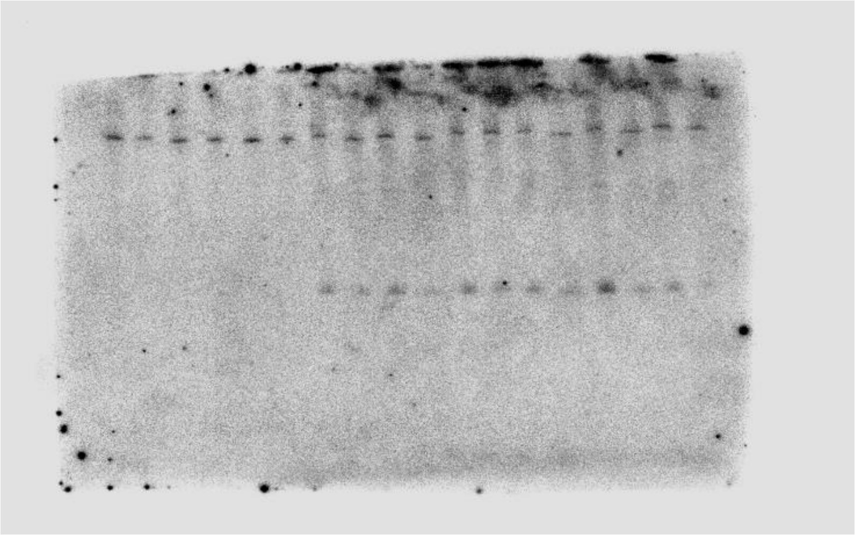

SV40-miR-S1 probe

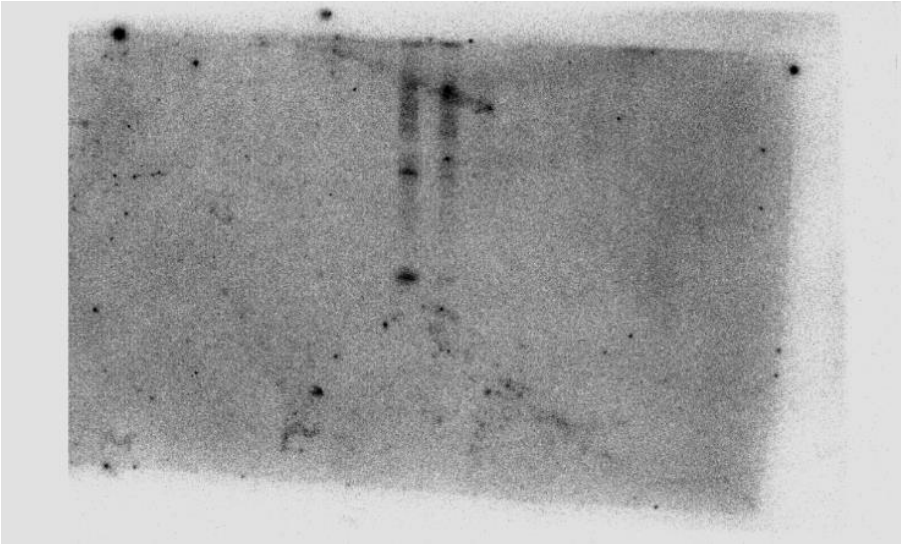

FcPV-miR-F1 3p probe

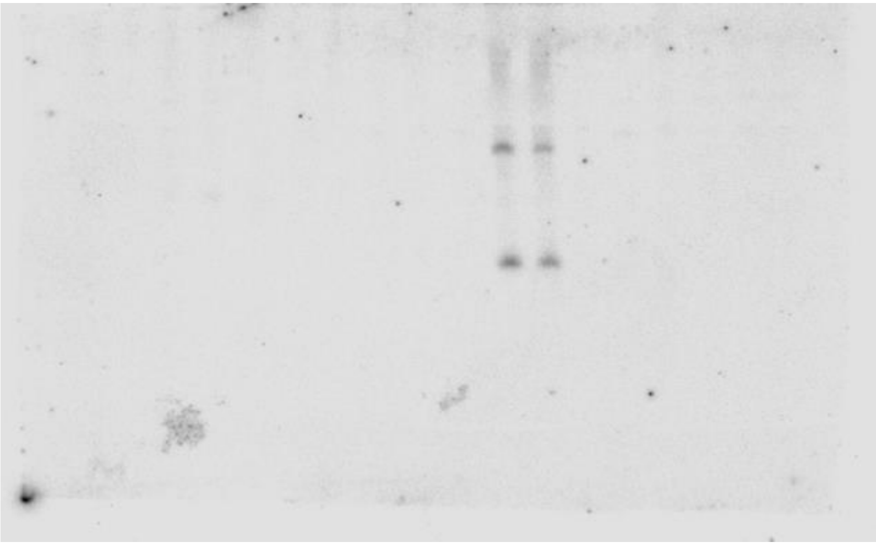

FcPV-miR-F2 3p probe

PV miRNA Drosha Dependence

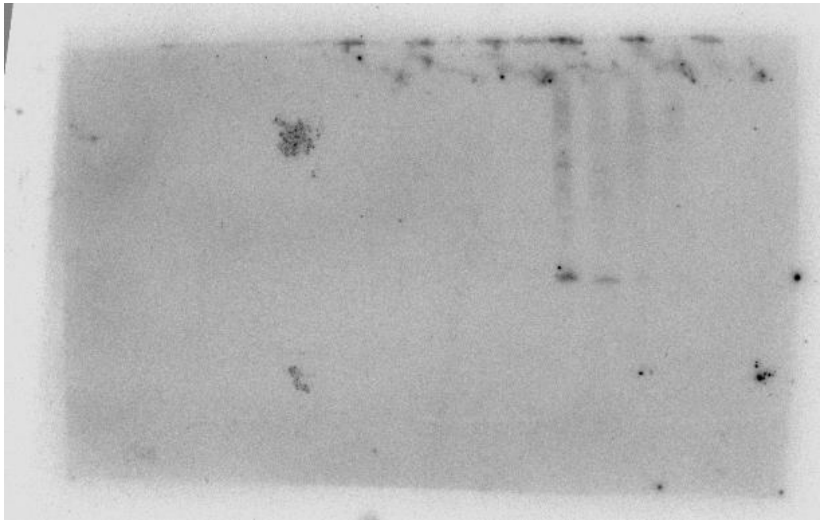

HPV17-miR-H1 3p probe

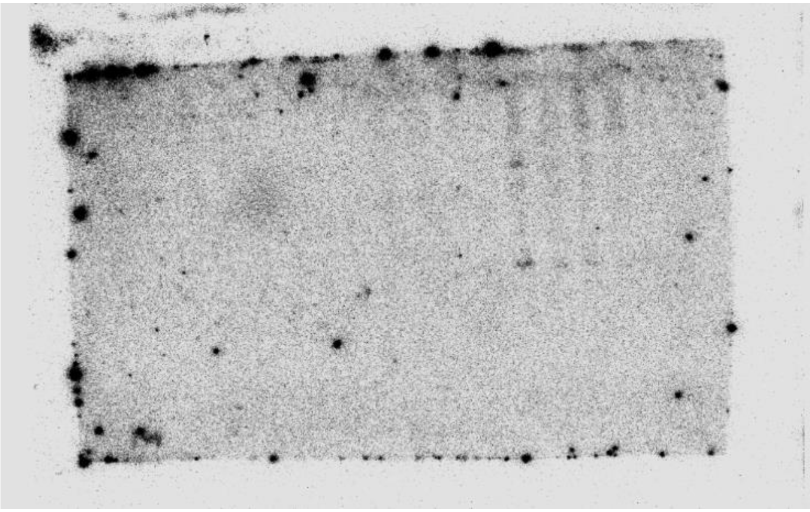

HPV37-miR-H1 3p probe

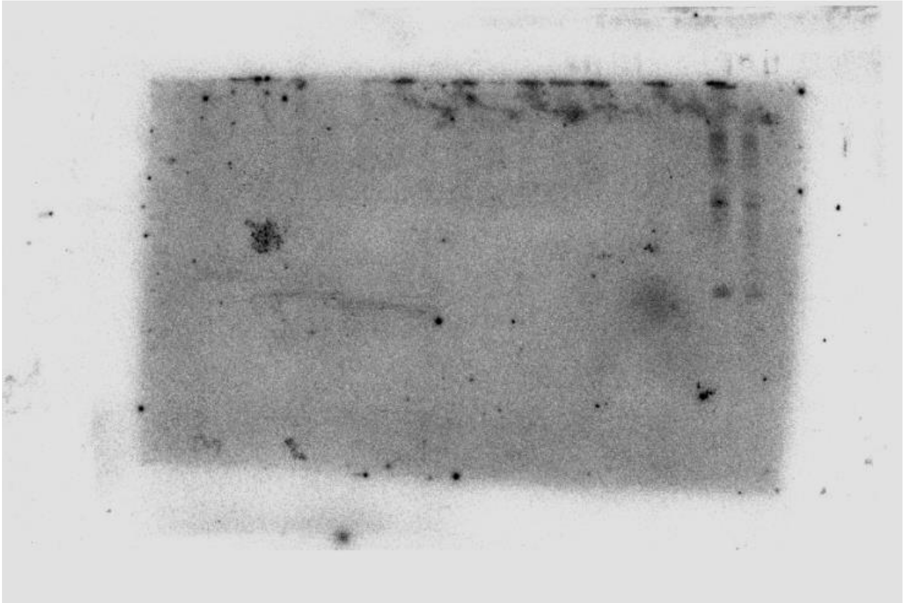

HPV41-miR-H1 5p probe

PV miRNA Dicer Dependence

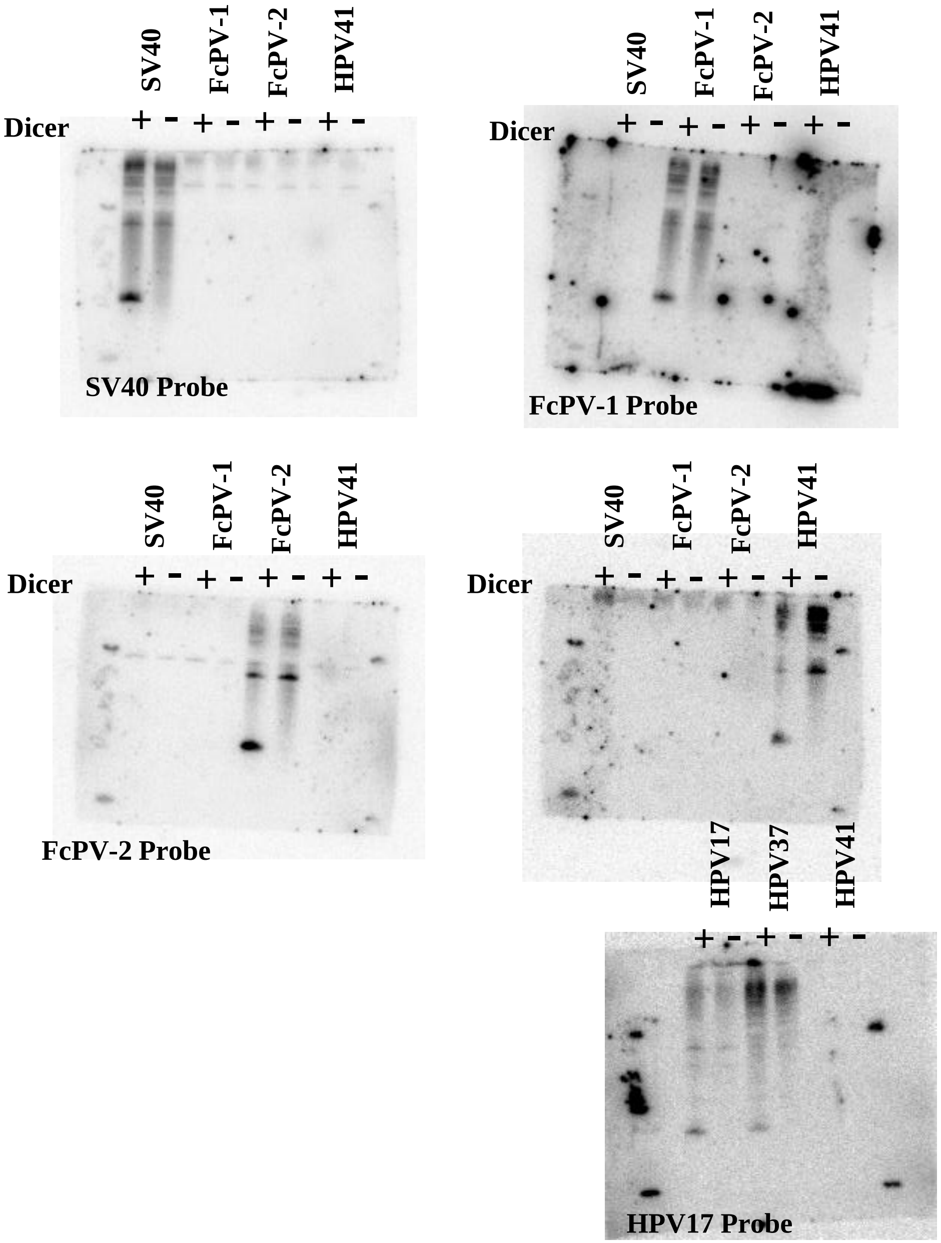

Supplement: S8 Dataset — This dataset includes the full uncropped scans of northern blots used in Fig 5. (PDF) [file ppat.1007156.s015.pdf]
